# Divergent evolutionary trajectories of bryophytes and tracheophytes from a complex common ancestor of land plants

**DOI:** 10.1038/s41559-022-01885-x

**Published:** 2022-09-29

**Authors:** Brogan J. Harris, James W. Clark, Dominik Schrempf, Gergely J. Szöllősi, Philip C. J. Donoghue, Alistair M. Hetherington, Tom A. Williams

**Affiliations:** 1 School of Biological Sciences, University of Bristol, Bristol, UK; 2 Bristol Palaeobiology Group, School of Earth Sciences, University of Bristol, Bristol, UK; 3 Department of Biological Physics, Eötvös Loránd University, Budapest, Hungary; 4 MTA-ELTE ‘Lendület’ Evolutionary Genomics Research Group, Budapest, Hungary; 5 Institute of Evolution, Centre for Ecological Research, Budapest, Hungary

**Keywords:** Phylogenetics, Plant evolution

## Abstract

The origin of plants and their colonization of land fundamentally transformed the terrestrial environment. Here we elucidate the basis of this formative episode in Earth history through patterns of lineage, gene and genome evolution. We use new fossil calibrations, a relative clade age calibration (informed by horizontal gene transfer) and new phylogenomic methods for mapping gene family origins. Distinct rooting strategies resolve tracheophytes (vascular plants) and bryophytes (non-vascular plants) as monophyletic sister groups that diverged during the Cambrian, 515–494 million years ago. The embryophyte stem is characterized by a burst of gene innovation, while bryophytes subsequently experienced an equally dramatic episode of reductive genome evolution in which they lost genes associated with the elaboration of vasculature and the stomatal complex. Overall, our analyses reveal that extant tracheophytes and bryophytes are both highly derived from a more complex ancestral land plant. Understanding the origin of land plants requires tracing character evolution across a diversity of modern lineages.

## Abstract (web summary)

Fossil calibrations, a relative clade age calibration (informed by horizontal gene transfer) and new phylogenomic methods for mapping gene family origins resolve tracheophytes (vascular plants) and bryophytes (non-vascular plants) as monophyletic sister groups that diverged during the Cambrian, 515–494 million years ago. The early evolution of both groups, but particularly that of bryophytes, was characterized by major gene content change.

## Main

The origin and early evolution of land plants (embryophytes) constituted a formative episode in Earth history, transforming the terrestrial landscape, the atmosphere and the carbon cycle^1,2^. Along with bacteria, algae, lichens and fungi^3^, land plants were fundamental to the creation of the earliest terrestrial ecosystems, and their subsequent diversification has resulted in more than 370,000 extant species^4^. Embryophytes form a monophyletic group nested within freshwater streptophyte algae^5^ and their move to land, while providing a new ecological niche, presented new challenges that required adaptation to water loss and growth against gravity^6^. Early innovations that evolved in response to these challenges include a thick waxy cuticle, stomata and a means of transporting water from the roots up vertically growing stems^2,5,7,8^. Modern land plants comprise two main lineages, vascular plants (tracheophytes) and non-vascular plants (bryophytes), that have responded to these evolutionary challenges in different ways.

The evolutionary origins of many gene families, including those of key transcription factors, have been shown to predate the colonization of land^9,10^. However, studies of gene family evolution within land plants have typically been restricted to individual gene families or sets of genes that encode single traits^11–16^. A lack of genome-scale data from non-flowering plants has also hindered efforts to reconstruct patterns of genome and gene content evolution more broadly across land plants^17^, although this challenge has been mitigated by the publication of large transcriptomic datasets^18^. Progress has also been made towards resolving the ambiguous phylogenetic relationships at the root of land plants^15,18–23^. The bryophyte fossil record has also undergone a radical reinterpretation such that there are now many more records with the potential to constrain the timescale of early land plant evolution^24–26^. Finally, new methods have been developed for timetree calibration based on the relative time constraints informed by horizontal gene transfer (HGT) events^27^.

Here we seek to exploit these advances in elucidating early land plant evolution. We first infer a rooted phylogeny of land plants using outgroup-free rooting methods and both concatenation and coalescent approaches. We then estimate an updated timescale of land plant evolution incorporating densely sampled fossil calibrations that reflect a revised interpretation of the fossil record. We extend this analysis using gene transfer events to better calibrate the timescale of hornwort evolution, a poorly constrained region of the land plant tree. By building on this dated phylogeny, we reconstruct the gene content evolution of bryophytes, tracheophytes and the ancestral embryophyte, revealing how key genes, pathways and genomes diverged during early land plant evolution.

## Results

### Complementary rooting approaches support the monophyly of bryophytes

**Extended Data Fig. 1:**
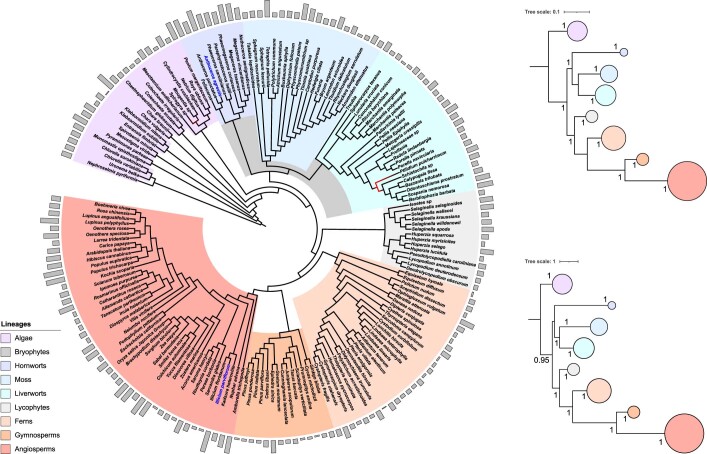
Phylogenetic analysis of land plants provides robust support for the monophyly of bryophytes. **a**, phylogenetic tree inferred from a concatenated alignment of 30919 sites consisting of 160 single copy orthogroups using the CAT-GTR model (Blanquart and Lartillot, 2008). Branch colour is proportional to the posterior probability; black branches received maximum support, and red received less than maximum and greater values than 0.9. The grey bars assigned to each species are proportional to the percentage of gaps in the alignment. Species with more than 50% gaps in the alignment have their labels coloured blue. The branches of the tree are not drawn to scale. **b**, Summarised maximum likelihood tree inferred from the same alignment as above using the LG + C60 + G4 + F model, which accounts for site heterogeneity in the substitution process. All major nodes received maximum boot strap support. **c**, Phylogenetic tree inferred using the ASTRAL; gene trees were inferred from the 160 single copy orthogroups used to construct the concatenate. All branches except the one defining bryophytes received maximum coalescent support, albeit the branch still received strong support (0.95). The size of the circles in both a and b are proportional to sample size of the lineage they represent.

A rooted phylogenetic framework is required to infer the nature of the ancestral embryophyte and to trace changes in gene content during the evolution of land plants. To that end, we compiled a comprehensive dataset of the published genome and transcriptome data from embryophytes and their algal relatives, and we inferred species trees using concatenation (PhyloBayes and IQ-TREE) and coalescent (ASTRAL) approaches (Supplementary Information). When the tree was rooted with an algal outgroup, we recovered bryophyte monophyly and a root between bryophytes and tracheophytes with high support across all analyses (Extended Data Fig. 1), in agreement with recent work^15,18,20,22,23,28^. However, rooting phylogenies with an outgroup can influence the ingroup topology due to long-branch attraction (LBA)^29–31^, where distantly related or fast-evolving taxa artifactually branch with the outgroup. LBA resulting from the large evolutionary distance between land plants and their algal relatives has previously been suggested as a possible cause of the difficulty in resolving the land plant phylogeny^32^. Indeed, outgroup-rooting analyses using different models^20,33^, datasets and molecules (that is, chloroplast, mitochondrial or nuclear sequences^22,28^) have provided support for conflicting hypotheses about the earliest-branching lineages and the nature of the ancestral land plant. LBA is thus a known artefact when recovering the land plant phylogeny.

**Fig. 1:**
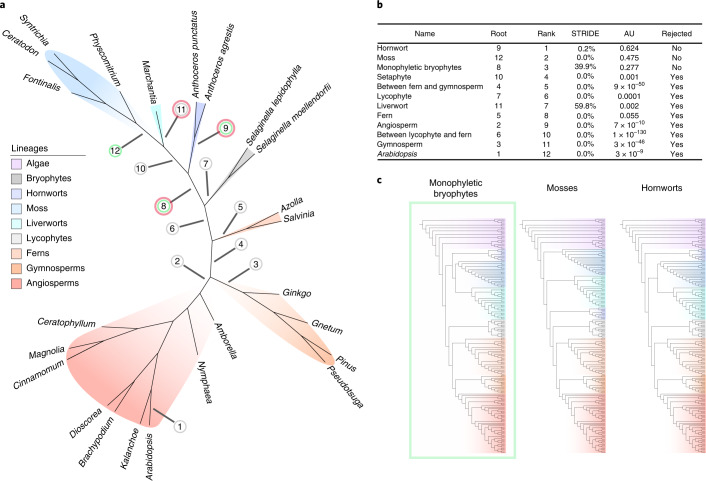
Investigating the root of embryophytes using outgroup-free rooting. **a**, An unrooted maximum likelihood tree was inferred from an alignment of 24 species and 249 single-copy orthogroups under the LG + C60 + G4 + F model^69^. Twelve candidate root positions for embryophytes were investigated using both ALE and STRIDE. For the ALE analysis, the unrooted tree was rooted in each of the 12 positions and scaled to geological time on the basis of the results of the divergence time analysis, and 18,560 gene clusters were reconciled using the ALEml algorithm^88^. The green circles highlight supported roots following the ALE analysis, while the red circles denote supported nodes in the STRIDE analysis. **b**, The likelihood of the 12 embryophyte roots was assessed with an AU test. The AU test significantly rejected 9 of the 12 roots, with roots on hornworts, moss and monophyletic bryophytes (root positions 9, 12 and 8, respectively) comprising the credible set. **c**, Phylogenetic trees constrained to the credible roots were inferred in IQ-TREE^69^ under the LG + C60 + G + F model. An AU test was used to evaluate the likelihood of each of the constrained trees^90^, with the root resulting in monophyletic bryophytes being the only one not to be significantly rejected.

**Fig. 2:**
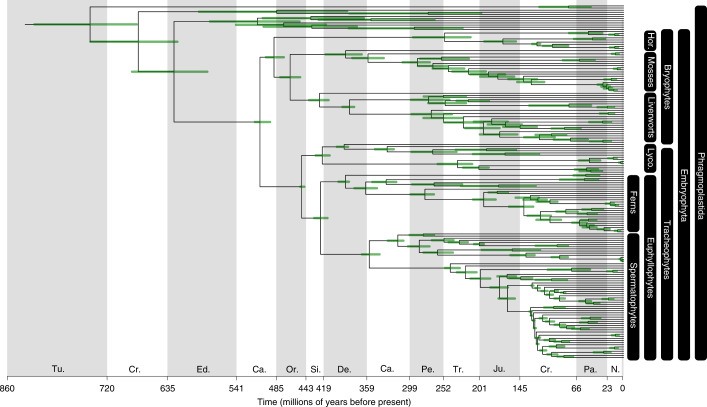
The timescale of land plant evolution. Divergence times in millions of years as inferred using a molecular clock model, 68 fossil calibrations and an HGT. The inference that the common ancestor of embryophytes lived during the Cambrian is robust to the choice of maximum age constraints (Supplementary Methods). The divergence times of hornworts are constrained by an HGT into polypod ferns, with the result that the hornwort crown is inferred to have diverged during the Permian–Triassic. The nodes are positioned on the mean age, and the bars represent the 95% highest posterior density.

**Extended Data Fig. 2:**
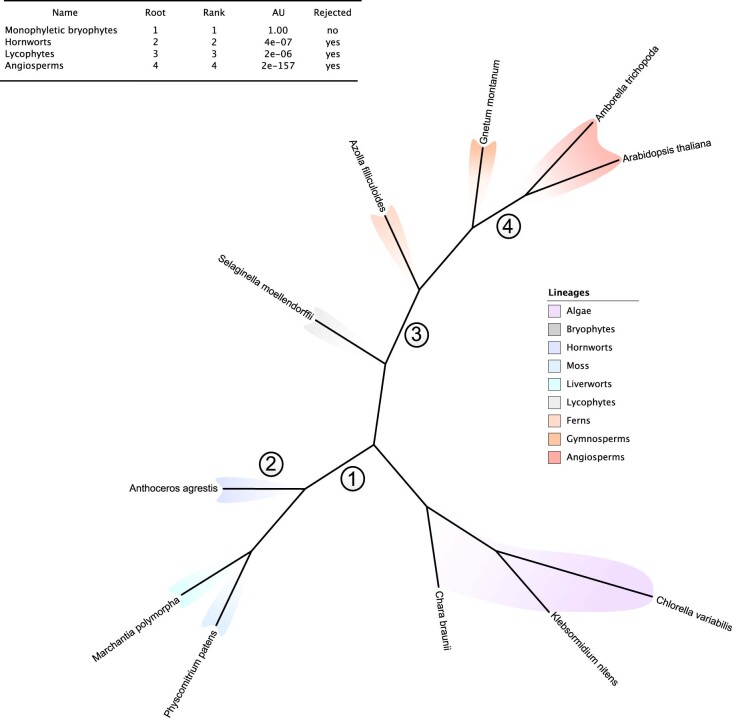
Additional outgroup-free rooting analyses. Unrooted maximum likelihood tree inferred from an alignment of 11 species and single copy orthogroups under the LG + C60 + G4 + F model. Four candidate root positions for embryophytes were investigated using ALE. For the ALE analysis, the unrooted tree was rooted in each of the twelve positions and scaled to geological time based on the results of the divergence time analysis and gene clusters were reconciled using the ALEml algorithm. The likelihood of the four embryophyte roots was assessed with an approximate unbiased (AU) test. The AU test significantly rejected 3 out of the 4 roots, favouring only a root between bryophytes and tracheophytes.

To address the impact of LBA and complement traditional outgroup-rooting analyses, we used two outgroup-free rooting methods—amalgamated likelihood estimation (ALE) and STRIDE^34,35^—to infer root placement on a dataset of 24 high-quality embryophyte genomes without the inclusion of an algal outgroup (Fig. 1). ALE calculates gene family likelihoods for a given root position under a model of gene duplication, transfer and loss (DTL)^34^; support for candidate root positions can then be evaluated by comparing their summed gene family likelihoods. STRIDE first identifies putative gene duplications in unrooted gene trees that can act as synapomorphies for post-duplication clades. The root of the species tree is then estimated using a probabilistic model that accounts for conflict among the inferred duplications^35^. Across 18,560 orthogroups, STRIDE recovered three most parsimonious roots: between bryophytes and tracheophytes, between liverworts and the remaining land plants and between hornworts and the remaining land plants (Fig. 1). Of these, the rooting on hornworts was assigned a 0.2% probability, on liverworts a 59.8% probability and between bryophytes and tracheophytes a 39.9% probability. To estimate root likelihoods using the ALE approach, we first used the divergence time estimates from the molecular clock analysis to convert branch lengths into units of geological time, allowing us to perform time-consistent reconciliations (that is, to prevent reconciliations in which gene transfers occur into the past). We reconciled 18,560 gene families under the 12 rooted and dated embryophyte trees (Fig. 1a) and used an approximately unbiased (AU) test (Fig. 1b) to evaluate support for the tested root positions. The AU test rejected 9 of 12 roots (*P* < 0.05; Fig. 2b and Supplementary Table 3), resulting in a credible set of three roots: the hornwort stem, the moss stem and a root between bryophytes and tracheophytes. These three credible roots are in close proximity on the tree, and root positions further from this region are rejected with increasing confidence (Fig. 1b and Supplementary Table 1). To evaluate the nature of the root signal for these three branches, we performed a family-filtering analysis in which families with high DTL rates were sequentially removed and the likelihood re-evaluated. The rationale for this analysis is that the evolution of these families may be poorly described by the model, and so they may contribute misleading signals^36^. In this case, the root order did not change after the removal of the high-DTL-rate families (Supplementary Fig. 1), suggesting broad support for these root positions from the data and analysis. Note that, in the ALE analysis, the moss and hornwort stems were accorded a higher summed gene family likelihood than was the branch separating bryophytes and tracheophytes, although the difference was not significant (hornwort stem log-likelihood, −824,522.9, *P* = 0.624; moss stem log-likelihood, −824,606.5, *P* = 0.475; bryophyte stem log-likelihood, −824709.1, *P* = 0.277). In a secondary analysis, we also used ALE to compare support for these different root positions in a smaller dataset of 11 genomes that included algal outgroups; in this analysis, all roots were rejected except for a root between tracheophytes and bryophytes (Extended Data Fig. 2, *P* < 0.05).

Finally, we constrained the topology of the tree inferred from the concatenated alignment to be in accordance with the three credible roots and computed the likelihood of sequence data along those trees. Trees with embryophyte roots constrained to hornworts and moss were significantly rejected (*P* < 0.05, AU test; Supplementary Table 2). The agreement between three rooting methods using different sources of information (outgroup placement, gene duplications alone and DTL events more broadly) therefore provides the most compelling support for a root between bryophytes and tracheophytes from our analyses. Taking our analyses together with other recent work^15,20,22,23,28^ suggests that a root between monophyletic tracheophytes and bryophytes is the best-supported hypothesis of land plant phylogeny. Bryophyte monophyly is therefore the default hypothesis with which to interpret land plant evolution.

### Combined fossil and genomic evidence, including an ancient HGT, calibrate the timescale of land plant evolution

**Extended Data Fig. 3:**
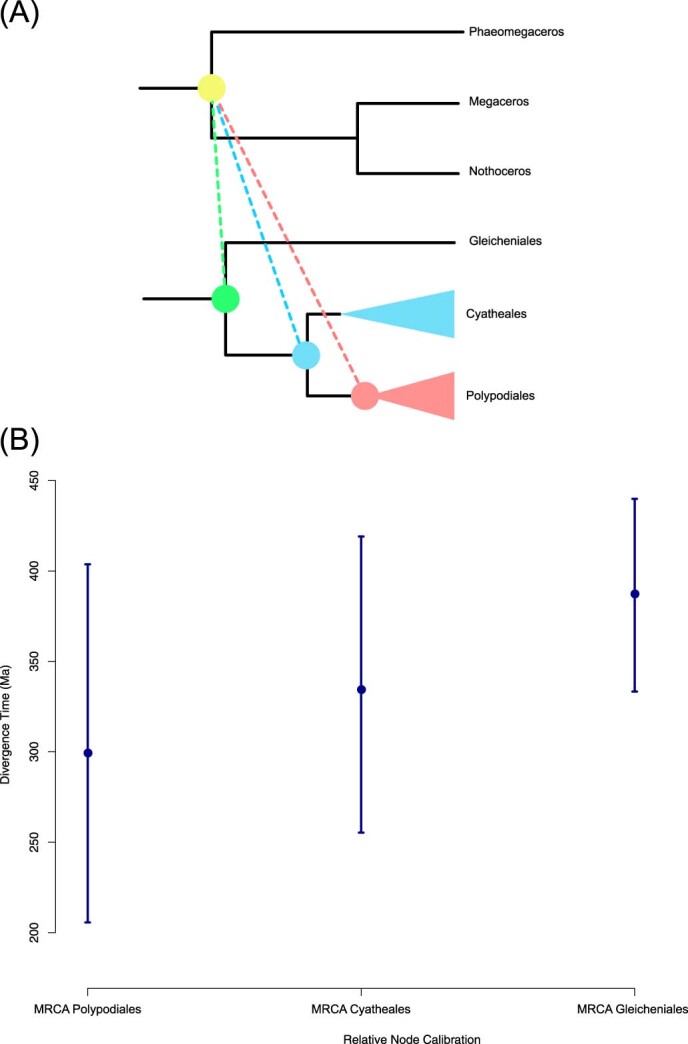
Alternative placements of the NEOCHROME constraint. The NEOCHROME horizontal gene transfer is predicted to have occurred from hornworts into the ancestor of polypod ferns. However, topological uncertainty in the NEOCHROME gene tree allows the possibility that the transfer could have occurred into a more ancient lineage (A). We placed the relative node calibration such that hornworts must be more ancient than (i) Polypodiales (ii) Cyatheales + Polypodiales and (iii) Gleicheniales+Cyatheales+Polypodiales. The 95% highest posterior density (HPD) for the molecular clock analysis under each scenario is shown as a bar in (B), with a dot for the mean age. 95% HPDs were calculated from 2,000 post-burnin samples over 2,000,000 MCMC generations.

We estimated divergence times on the resolved land plant phylogeny (Fig. 2). We assembled a set of 68 fossil calibrations, representing every major lineage of land plant and notably sampling more bryophyte fossils than previous studies (Supplementary Methods). Despite this increased sampling, the fossil record of hornworts remains particularly sparse, and no fossils unambiguously calibrate the deepest branches within the clade. To ameliorate the limitations of the fossil record, we implemented a relative node age constraint based on the horizontal transfer of the chimaeric photoreceptor NEOCHROME from hornworts into ferns^37^. To account for uncertainty in the timing of the gene transfer, we evaluated the impacts of several possible scenarios on our analyses (Extended Data Fig. 3). In the absence of direct fossil calibrations for hornworts, this gene transfer provides a relative constraint that ties the history of hornworts to that of ferns, for which more fossils are available.

Our results are congruent with those of previous studies^38^ but offer greater precision on many nodes and in some cases greater accuracy (Supplementary Fig. 2). This has been leveraged by a denser sampling of fossil calibrations, improved taxonomic sampling (especially among bryophytes), relative calibration of hornworts using the NEOCHROME HGT, and the ability to condition divergence times on a single topology.

**Extended Data Fig. 4:**
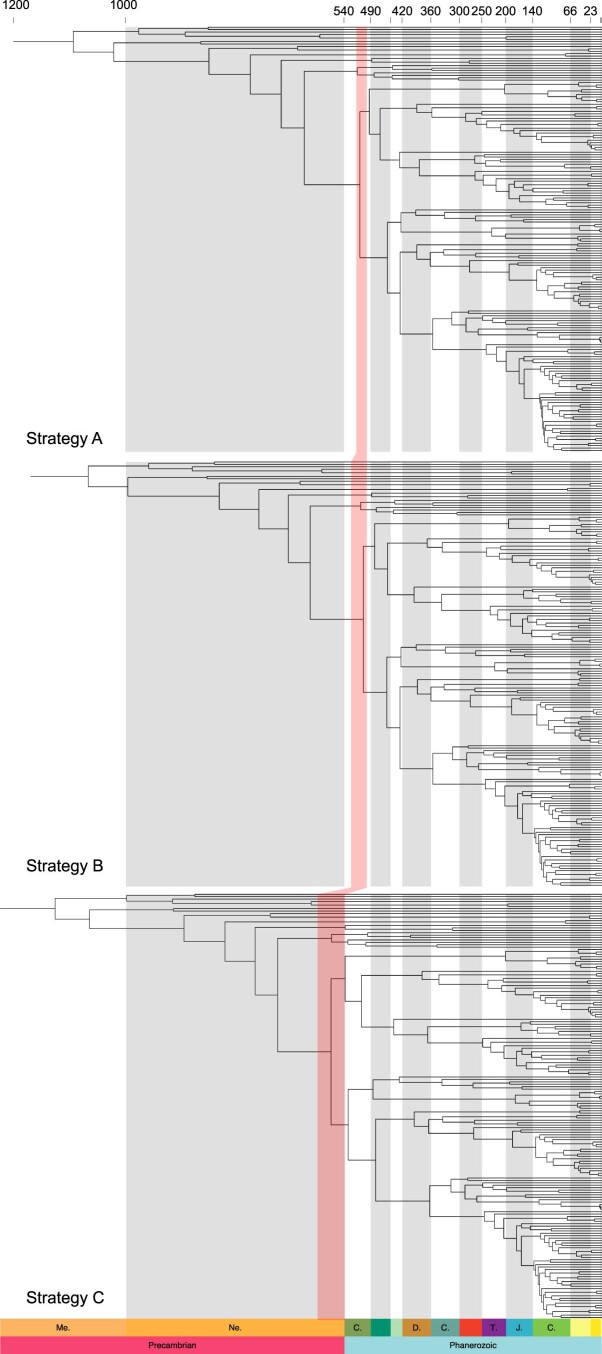
The effect of alternative calibration strategies on the age of crown group embryophytes. Calibrations were altered by variously relaxing maximum age calibrations on the age of embryophytes (Strategy B) and embryophytes and tracheophytes (Strategy C). The width of the red band across the phylogenies represents the 95% highest posterior density (HPD) interval. 95% HPDs were calculated from 2,000 post-burnin samples over 2,000,000 MCMC generations.

The role and influence of fossil calibrations in molecular clock studies, especially maximum age calibrations, remain controversial^23,39,40^. While the fossil record is an incomplete representation of past diversity, our analyses account for this uncertainty in the form of soft minima and maxima. Morris et al.^38^ inferred a relatively young age for the embryophyte crown ancestor (515–470 million years ago (Ma)), making use of a maximum age constraint based on the absence of embryophyte spores in strata for which fossilization conditions were such that spores of non-embryophyte algae have been preserved. Hedges et al.^39^ and Su et al.^23^ argued against the suitability of this maximum age constraint on the basis that calibrations derived from fossil absences are unreliable and that the middle Cambrian maximum age exerts too great an influence on the posterior estimate^8,41^. To assess the sensitivity of our approach to the effect of maximum age calibrations, we repeated the clock analyses with less informative maximum age calibrations (Supplementary Methods). Removing the maximum age constraint on the embryophyte node produced highly similar estimates to when the maximum is employed (Extended Data Fig. 4). Relaxing all maxima did result in more ancient estimates for the origin of embryophytes, although still considerably younger than recent studies^23^, extending the possible origin for land plants back to the Ediacaran (540–597 Ma; Extended Data Fig. 4). The older ages estimated in Su et al.^23^ seem to reflect, in part, differences in the phylogenetic assignment of certain fossils (Supplementary Methods), such as the putative algae *Proterocladus antiquus* and the liverwort *Ricardiothallus devonicus*, rather than a dependence on the maximum age calibration. Our results reject the possibility that land plants originated during the Neoproterozoic, instead supporting an origin of the land plant crown group during the mid-late Cambrian, 515–493 Ma, with crown tracheophytes and crown bryophytes originating 452–447 Ma (Late Ordovician) and 500–473 Ma (late Cambrian to Early Ordovician), respectively. Within bryophytes, the divergence between Setaphyta (mosses + liverworts) and hornworts occurred by 479–450 Ma (Ordovician), with the radiation of crown mosses by 420–364 Ma (latest Silurian to Late Devonian) and crown liverworts 440–412 Ma (early Silurian to Early Devonian). Among tracheophytes, the crown ancestor of lycophytes is dated to the middle Silurian to Early Devonian, 431–411 Ma, coincident with that of euphyllophytes 432–414 Ma.

The calibration of hornwort diversification using the NEOCHROME HGT had a substantial impact on inferences of stem and crown group age. In the absence of fossil calibrations on deep nodes, hornworts are characterized by an ancient stem lineage and the youngest crown lineage among land plants^38,42^. The effect of the relative age constraint is to make the crown group older (294–214 Ma; Fig. 2) and thus shorten the length of the stem, with divergence times within the crown group all moving older. We repeated the analysis with alternative placements for the relative time constraint, with the age of crown hornworts becoming increasingly ancient when the transfer was placed into the ancestor of more inclusive clades, Cyatheales + Polypodiales (258–419 Ma) or before the divergence of Gleicheniales from the Cyatheales + Polypodiales clade (331–445 Ma), respectively (these scenarios are illustrated in Extended Data Fig. 3). All of these estimates considerably predate the earliest unequivocal fossils assigned to hornworts. However, given the scarcity of hornwort fossils, it seems likely that this clade is older than a literal reading of the fossil record might suggest.

### Gene content of the embryophyte common ancestor

We used gene-tree/species-tree reconciliation to estimate the gene content of the embryophyte common ancestor (Supplementary Tables 3–5). We used the genome dataset from the ALE rooting analysis with the addition of five algal genomes, to better place the origin of families that predate the origin of embryophytes (Supplementary Fig. 3). The tree was dated following the same methodology as the larger dating analysis while using an applicable subset of calibrations, allowing the use of a dated reconciliation algorithm (ALEml) to improve the estimation of DTL events (Supplementary Fig. 4).

**Fig. 3:**
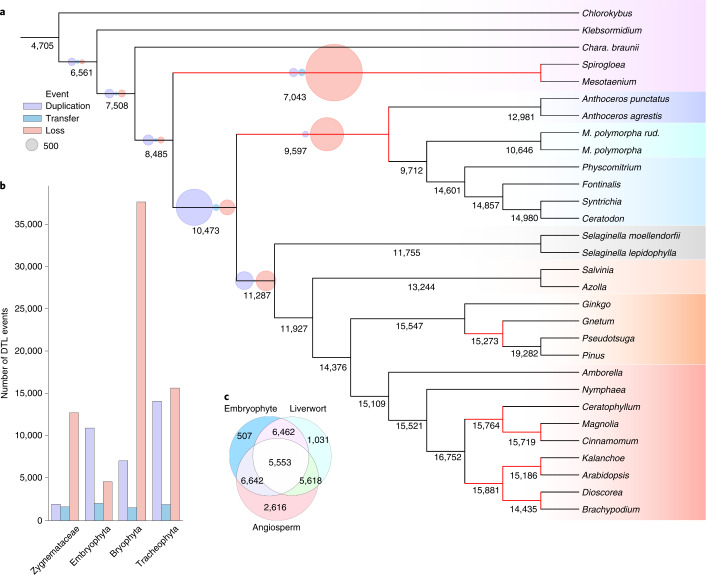
Gene content reconstruction of the ancestral embryophyte. **a**, Ancestral gene content was inferred for the internal branches of the embryophyte tree. A maximum likelihood tree was inferred from an alignment of 30 species of plants and algae, comprising 185 single-copy orthologues and 71,855 sites, under the LG + C60 + G4 + F model in IQ-TREE^69^, and rooted in accordance with our previous phylogenetic analysis. A timescale for the tree was then calculated using a subset of 18 applicable fossil calibrations in MCMCtree. We reconciled 20,822 gene family clusters, inferred using Markov clustering^87^, against the rooted dated species tree using the ALEml algorithm^88^. The summed copy number of each gene family (under each branch) was determined using custom Python code (branchwise_number_of_events.py). Branches with reduced copies from the ancestral node are coloured in red. The numbers of DTL events are represented by purple, blue and red circles, respectively. The sizes of the circles are proportional to the summed number of events (the scale is indicated by the grey circle). **b**, The number of DTL events scaled by time for four clade-defining branches in the embryophyte tree. **c**, The number of shared gene families between the ancestral embryophyte, liverwort and angiosperm. The ancestral embryophyte shares more gene families with the ancestral angiosperm than with the ancestral liverwort.

The analysis of ancestral gene content highlighted considerable gene gain along the ancestral embryophyte branch (Fig. 3a and Supplementary Table 3). A substantial number of duplications defined this transition, with fewer transfers and losses observed. Our analysis suggests that the common ancestor of embryophytes and Zygnematales had more of the building blocks of plant complexity than extant Zygnematales, which have undergone a loss of 1,442 gene families since their divergence, the largest loss observed on the tree (Fig. 3a). Functional characterization of the genes lost in the Zygnematales using the KEGG database identified gene families involved in the production of cytoskeletons, exosomes and phenylpropanoid synthesis (Supplementary Table 6). Exosomes and complex cytoskeletons are essential for multicellular organisms to function^43,44^, and the inferred loss of these gene families is consistent with the hypothesis that the body plan of the algal ancestor of embryophytes was multicellular^5^, rather than possessing the single-cell or filamentous architecture observed in extant Zygnematales. The more complex cytoskeleton could be associated with increased rigidity, helping overcome the gravitational and evaporative pressures associated with the transition to land^6^. Interestingly, phenylpropanoids are associated with protection against UV irradiance^45^ and homiohydry^5^, suggesting that the common ancestor may have been better adapted to a terrestrial environment than extant Zygnematales.

**Extended Data Fig. 5:**
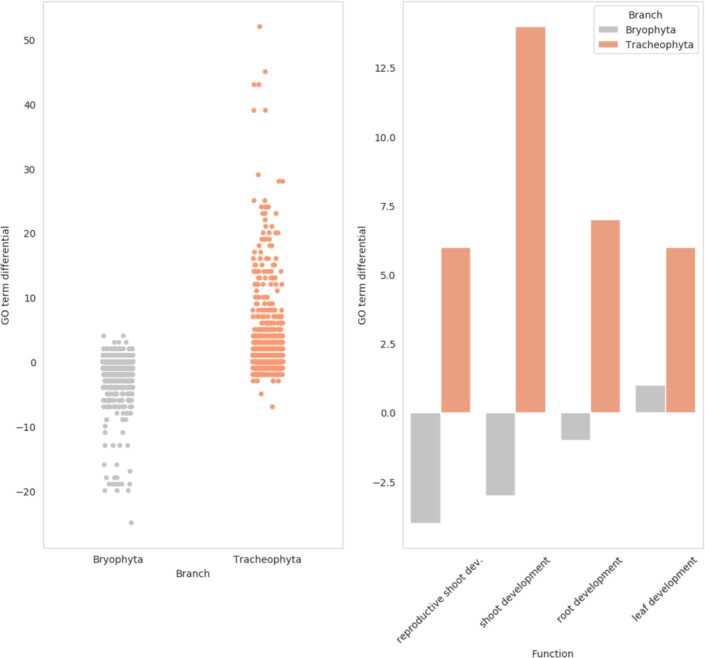
Functional annotation of gene family changes between the ancestral embryophyte, bryophytes and tracheophytes. Left, overall change in GO term frequency between the ancestral embryophyte and the ancestral bryophyte/tracheophyte. GO terms on average become less frequent in bryophytes. Right, change in the frequency of specific GO terms between the ancestral embryophyte and the ancestral bryophyte/tracheophyte. Bryophytes have a reduction in gene families associated with shoot and root development, while we see an increase in gene families associated with these GO terms in the tracheophyte ancestor.

**Extended Data Fig. 6:**
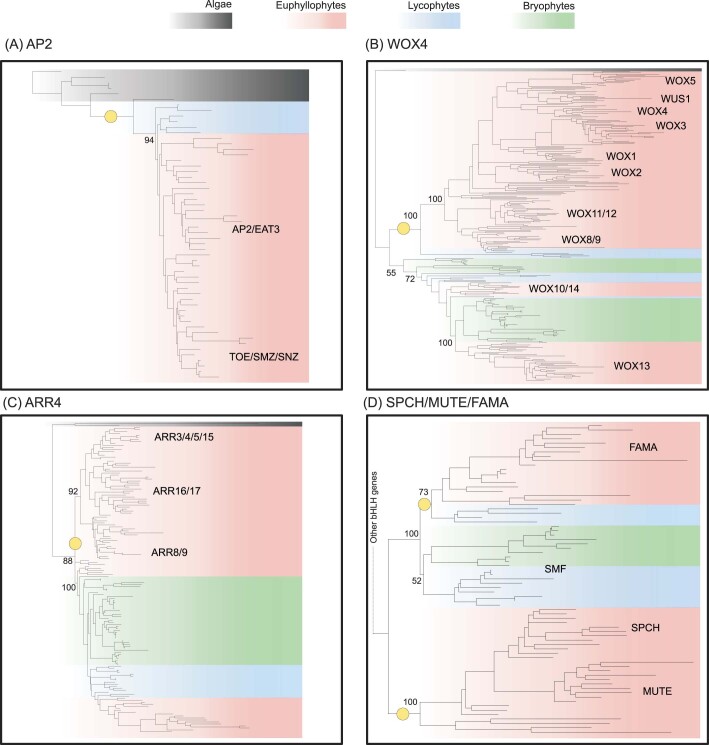
Phylogenetic trees of key losses on the bryophyte stem. Gene trees were constructed from BLAST searches of an expanded taxon set. Each gene tree was inferred under the best-fitting model in IQ-TREE determined via the Bayesian Information Criterion. The trees were rooted using algal outgroups. In each case, the branches where bryophytes appear to have undergone loss are marked by a yellow dot.

We also observed greater gene loss along the bryophyte stem lineage (Fig. 3a and Supplementary Tables 3, 7 and 8), with the rate of gene loss (in terms of gene families per year) substantially greater than in all other major clades (Fig. 3b). It is important to note that inferences of gene loss from large-scale analyses are sensitive to the approach used to cluster sequences and define gene families; current approaches are not consummate. We therefore sought to evaluate the robustness of our conclusions using a range of sensitivity analyses (Supplementary Figs. 5–8). These suggested that, while the number of inferred gene losses on the bryophyte stem varies, it remains an event of major gene loss under all conditions tested. We also observed considerable losses along the tracheophyte stem, countered by a greater number of duplications (Supplementary Table 9). This suggests a period of genomic upheaval on both sides of the embryophyte phylogeny. Gene Ontology (GO) term functional annotation of the gene families lost in bryophytes reveals reductions in shoot and root development from the ancestral embryophyte (Supplementary Table 7 and Extended Data Fig. 5). To investigate the evolution of genes underlying morphological differences between tracheophytes and bryophytes, we evaluated the evolutionary history of gene families containing key *Arabidopsis* genes for vasculature and stomata (Supplementary Table 10). Gene families associated with both vasculature and stomatal function exhibited lineage-specific loss in bryophytes (Supplementary Figs. 9 and 10). Specifically, four orthologous gene families that are involved in the determination of the *Arabidopsis* body plan, containing WOX4, SPCH/MUTE/FAMA, AP2 and ARR, were inferred to be lost on the bryophyte stem (Supplementary Table 10). To investigate these inferred losses in more detail, we manually curated sequence sets and inferred phylogenetic trees for these families (Supplementary Methods and Extended Data Fig. 6). These analyses of individual gene families corroborated the pattern of loss along the branch leading to bryophytes. The loss of these orthologous gene families strengthens the hypothesis that ancestral embryophytes had a more complex vasculature system than that of extant bryophytes^8^. Overall, the loss of gene families (Fig. 3) and the change in GO term frequencies (Extended Data Fig. 5) suggest a widespread reduction in complexity in bryophytes, and the ancestral embryophyte being more complex than previously envisaged. Indeed, gene loss defines the bryophytes early in their evolutionary history, but large numbers of duplication and transfer events are observed following the divergence of the setaphytes and hornworts (Supplementary Table 3), with (for example) extant mosses boasting a similar gene copy number to tracheophytes (Fig. 3).

## Discussion

We have presented a time-scaled phylogeny for embryophytes, which confirms the growing body of evidence that bryophytes form a monophyletic group (Fig. 1), and our precise estimates of absolute divergence times provide a robust framework to reconstruct genome evolution across early land plant lineages (Fig. 2). Our results confirm that many well-characterized gene families predate the origin of land plants^9,10,15,46,47^. However, our analyses also show that extensive gene loss has characterized the evolution of major embryophyte groups. Reductive evolution in bryophytes has been demonstrated previously, where the loss of several genes has resulted in the lack of stomata^15,48^.

Our results suggest that these patterns of gene loss are not confined to stomata but are instead pervasive across bryophyte (and tracheophyte) genomes, and that much of the genome reduction occurred during a relatively brief period of ~20 million years following their divergence from tracheophytes during the Cambrian. While the balance of evidence favours bryophyte monophyly, it is interesting to note that the inference of high levels of gene loss in bryophytes is not contingent on this hypothesis: extensive within-bryophyte gene loss was inferred under all three of the roots within the credible region identified in the ALE analysis (Supplementary Table 11). These findings point to contrasting dynamics of genome evolution between the two major land plant lineages, with bryophytes demonstrating a net loss of genes, whereas gene loss is balanced by duplication in tracheophytes. The evolutionary pressures that underlay this ‘Cambrian implosion’ and the ways in which gene loss contributed to the evolution of the bryophyte body plan (such as the loss of genes associated with vasculature) remain unclear. It has been proposed that the radiation of vascular plants, heralded by the increased diversity of trilete spores in the palynological record, relegated bryophytes to a more marginal niche^49^. However, it seems possible that bryophytes independently evolved to exploit this niche, shedding the molecular and phenotypic innovations of embryophytes where they were no longer necessary. A large body of research has focused on the importance of gene and whole-genome duplication in generating evolutionary novelty in land plant evolution^50–53^. However, gene loss is an important driver of phenotypic evolution in other systems^54–56^, notably in flying and aquatic mammals^57^ and yeast^58^. It has also been shown that rates of genome evolution, rather than absolute genome size, correlate with diversification across plants^59^. Extant bryophytes remain highly diverse, and it is possible that bryophytes represent another example of specialization and evolutionary success via gene loss.

**Fig. 4:**
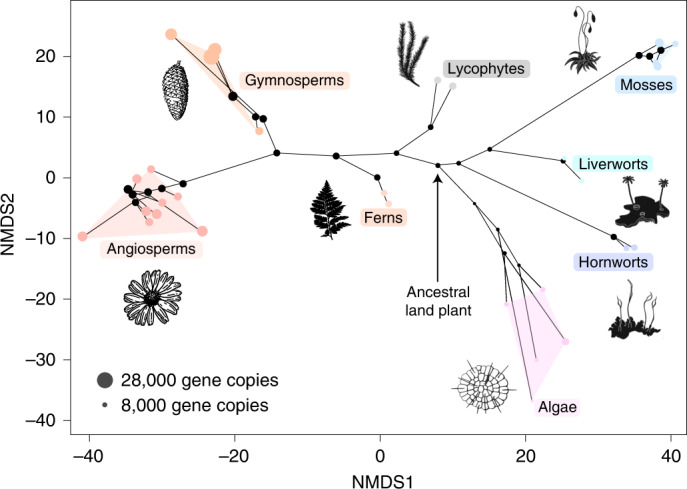
Genome disparity analysis demonstrates that the gene content of both tracheophytes and bryophytes is highly derived. Non-metric multidimensional scaling (NMDS) analysis of the presence and absence of gene families. The presence or absence of each gene family was determined from the ALE analysis for each tip and internal node in the phylogeny. The presence/absence data were used to calculate the Euclidean distances between species and nodes, which were then ordinated using NMDS. Branches were drawn between the nodes of the tree, with convex hulls fitting around members of each major lineage of land plants.

Bryophytes have sometimes been used as models in physiological and genetic experiments to infer the nature of the ancestral land plant. Our analysis suggests that modern bryophytes are highly derived: in terms of gene content, our analysis suggests that the ancestral angiosperm may have shared more genes with the ancestral land plant than did the ancestral liverwort (Fig. 3c). Such differences in gene content between species can be visualized as an ordination, where the two-dimensional distances between species represent dissimilarity in gene content. Reconstructed gene content at ancestral nodes can be projected into this space, showing the evolution of gene content along the phylogeny (Fig. 4). These genome disparity analyses reveal that the genomes of bryophytes and tracheophytes are both highly derived. Neither lineage occupies an ancestral position, with lineage-specific gene gain and loss events driving high disparity in both bryophytes and tracheophytes, reinforcing the view that there are no extant embryophytes that uniquely preserve the ancestral state^20,21,60^. Despite the paucity of data for some groups, these analyses reveal that the diversity among bryophyte genomes is comparable to that among tracheophyte genomes. These results are perhaps unsurprising given that bryophytes have been evolving independently of tracheophytes since the Cambrian and the similarly ancient divergence of each of the major bryophyte lineages, but they emphasize the point that, in general terms, bryophytes serve as no better a proxy for the ancestral land plant than do tracheophytes. Our results therefore agree that a view of bryophytes as primitive plants may mislead inferences of ancestral gene content or character evolution^20,61^. Instead, the best model organism(s) for investigating the nature of early plants will depend on the trait being investigated, alongside a careful appraisal of the phylogenetic diversity, including algal outgroups. Likewise, interpretations of the early land plant fossil record have been contingent on the first land plants appearing more like extant bryophytes than tracheophytes. That the ancestral embryophyte may have been more complex than living bryophytes is in keeping with many early macrofossils being more complex than bryophytes and possessing a mosaic of tracheophyte and bryophyte traits^8,62^.

## Methods

### Sequence data

An amino acid sequence dataset was assembled for the outgroup rooting analysis composed of 177 species, with 23 algae and 154 land plants (Supplementary Table 12). The sequence data were obtained from published transcriptomes^18,63^ or whole-genome sequences from the NCBI repository^64^. For the outgroup-free rooting, a second dataset of 24 whole genomes consisting solely of land plants was constructed (Supplementary Table 13). A further 6 genomes, comprising 1 land plant and 5 algae, were used to infer the ancestral gene content across land plants (Supplementary Table 13). The completeness of each genome or transcriptome was assessed using the BUSCO algorithm and the Viridiplantae library^65^, with completeness measured as the percentage of present BUSCO genes (Supplementary Tables 12 and 13 and Supplementary Figs. 11–14).

### Software

All custom Python scripts used in the current study are available at https://github.com/ak-andromeda/ALE_methods/. Software usage is described in the PDF document ALE_methods_summary.pdf in the GitHub folder along with a demonstration dataset.

### Orthologue inference

Orthologous gene families were inferred with OrthoFinder^66^; no universally present single-copy orthologous gene families were recovered. Instead, we used a custom Python program (prem3.py) to systematically compute low-copy-number orthologous gene families and from these identify suitable gene families for phylogenomic analyses (Supplementary Methods and Supplementary Fig. 15). This approach yielded 160 single-copy gene families from 114,016 orthogroups.

### Phylogenetics

#### Supermatrices

We aligned 160 single-copy gene families using MAFFT^67^, and poorly aligning sites were identified and removed with BMGE using the BLOSUM30 matrix^68^. For the maximum likelihood analyses, we used the best-fitting substitution model as selected by the Bayesian information criterion (LG + C60 + G4 + F) in IQ-TREE (version 1.6.12)^69,70^; the Bayesian analyses were performed under the CAT + GTR + G4 model in PhyloBayes version 2.3 (ref. ^71,72^). These models accommodate site-specific amino acid compositions via a fixed number of empirical profiles (C60) or an infinite mixture of profiles (CAT)^73,74^.

#### Supertrees

Individual maximum likelihood gene trees were inferred for each of the 160 single-copy gene families in IQ-TREE^69^, using the best-fitting model, selected individually for each gene using the Bayesian information criterion. A supertree was then inferred using ASTRAL version 5.7.6 (ref. ^75^).

### Divergence time estimation

Molecular clock methods represent one of the only credible means of obtaining an evolutionary timescale, integrating molecular and palaeontological evidence bearing on the phylogenetic and temporal relationships of living clades. Molecular clock methods see through the gaps in the fossil record to the timing of divergence of molecular loci. One feature of any molecular clock analysis is that, in the absence of admixture or gene transfer, the divergence of gene lineages must logically occur prior to the divergence of the organismal lineages that contain them^76^. Molecular clock branch lengths inferred from concatenates represent an average across loci, and the distinction between gene and lineage divergences is not modelled. The discrepancy between the two ages is unclear, but it is probably small and encompassed by the uncertainties associated with molecular clock estimates.

Estimates of the origins of major lineages of land plants have proven robust to different phylogenetic hypotheses^38,39^, but not to different interpretations of the fossil record^23,38,39^. Some recent studies of the timing of land plant evolution have argued that fossil calibrations should not exert undue influence over divergence time estimates^23,40^. However, in the absence of fossil calibrations, relaxed molecular clocks fail to distinguish rate and time, and fossil calibrations are therefore important across the tree to inform rate variation and in turn increase the accuracy of age estimates^77^. Our approach thus sought to maximize the information in the fossil record and increase the sampling of fossil calibrations over previous studies^23,38^.

Minimum age calibrations were defined on the basis of the oldest unequivocal evidence of a lineage. Specifying a maximum age calibration is considered controversial by some^23,39^, yet maximum ages are always present, either as justified user-specified priors or incidentally as part of the joint time prior^78,79^. On this basis, we defined our maxima following the principles defined in Parham et al.^80^, and fossil calibrations were defined as minimum and maximum age constraints, in each case modelled as uniform distributions between minima and maxima, with a 1% probability of either bound being exceeded (Supplementary Methods). We fixed the tree topology to that recovered by the Bayesian analysis and used the normal approximation method in MCMCtree (v. 4.9i) [81], with branch lengths first estimated under the LG + G4 model in codeml (v 4.9i) ^81^. We divided the gene families into four partitions according to their rate, determined on the basis of the maximum likelihood distance between *Arabidopsis thaliana* and *Ginkgo biloba*. We implemented a relaxed clock model (uncorrelated; independent gamma rates), where the rates for each branch are treated as independent samples drawn from a lognormal distribution. The shape of the distribution is assigned a prior for the mean rate (*μ*) and for the variation among branches (*σ*), each modelled as a gamma-distributed hyperprior. The gamma distribution for the mean rate was assigned a diffuse shape parameter of 2 and a scale parameter of 10, on the basis of the pairwise distance between *Arabidopsis thaliana* and *Ginkgo biloba*, assuming a divergence time of 350 Ma^38^. The rate variation parameter was assigned a shape parameter of 1 and a scale parameter of 10. The birth and death parameters were each set to 1, specifying a uniform kernel^82^. Four independent Markov chain Monte Carlo runs were performed, each running for four million generations to achieve convergence. Convergence was assessed in Tracer(v 1.7.1) ^83^ by comparing posterior parameter estimates across all four runs and by ensuring that the effective sample sizes exceeded 200.

### Temporal constraint from a hornwort-to-fern HGT

**Extended Data Fig. 7:**
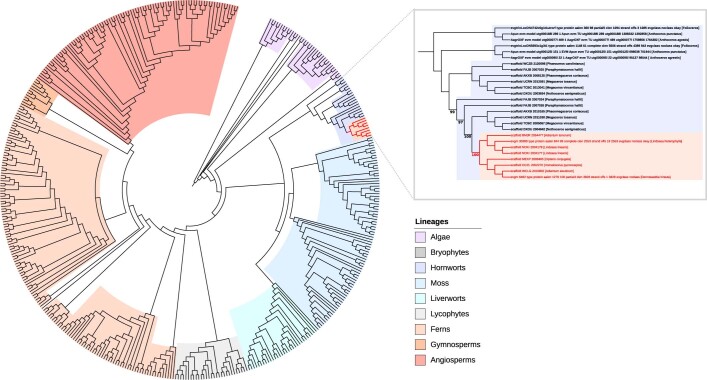
Phylogenetic tree highlighting the horizontal transfer of the chimeric neochrome photoreceptor (NEO). The *Arabidopsis thaliana* protein sequence for PHOT1 was used to BLAST a database of 177 species of plant and transcriptomes. The homologous sequences were aligned with MAFFT and trimmed with BMGE. A maximum likelihood tree was inferred in IQ-TREE under the best fitting substitution model inferred with Bayesian Inference Criterion. 8 fern genes were resolved within the hornworts and were inferred to have undergone horizontal gene transfer (coloured red). This transfer was previously characterised (Li *et al*., 2014), and we corroborate this finding with maximum bootstrap support.

HGT events provide information about the order of nodes on a species phylogeny in time over and above the ancestor–descendent relationships imposed by a strictly bifurcating phylogenetic species tree. Consequently, inferred HGT events can be used as relative node order constraints between divergent scions^27^; this is especially useful when fossil calibrations are not uniformly distributed across a tree. We used the horizontal transfer of the chimaeric neochrome photoreceptor (NEO) from hornworts to a derived fern lineage (Polypodiales)^84^ as an additional source of data about divergence times in hornworts, a lineage that diverged early in plant evolution but is poorly represented in the fossil record. We inferred a new gene tree for NEO using the expanded sampling of lineages now available, which confirmed the donor and recipient lineages originally reported^84^ (Extended Data Fig. 7). The gene tree topology for the NEOCHROME family reveals discordance between the species and gene trees for some relationships within the ferns, with copies present in some earlier-diverging lineages, including gleichenioid and tree ferns (Extended Data Fig. 7). This suggests that some duplication and loss, or perhaps within-fern transfer, may have occurred in this family. As a result, while the gene was most likely acquired in the common ancestor of Polypodiales, transfers into Gleicheniales or Cyatheales cannot be excluded entirely. We repeated the analysis with the relative time constraint reflecting each of these possibilities.

This relative node order constraint was used together with the 66 fossil calibrations in a Bayesian inference program (mcmc-date, https://github.com/dschrempf/mcmc-date) to infer a species tree with branch lengths measured in absolute time. In contrast to MCMCtree, mcmc-date uses the posterior distribution of branch lengths estimated by PhyloBayes, as described above, together with a multivariate normal distribution accounting for correlations between branches, to approximate the phylogenetic likelihood. Furthermore, an exponential hyperprior with mean 1.0 was used for the birth and death rates, as well as for the mean and variance of the gamma prior of the branch rates. A tailored set of random-walk proposals executed in random order per iteration, and the Metropolis-coupled Markov chain Monte Carlo algorithm^85^ with four parallel chains, resulted in near independence of consecutive samples. After a burn-in of approximately 5,000 iterations, 15,000 iterations were performed. All inferred parameters and node ages have effective sample sizes above 8,000 as calculated by Tracer. Subsequently, the relative node dating analysis and the partitioned molecular clock analysis were combined by using the posterior distributions for the divergence times within hornworts from the relative node dating as a prior for the partitioned analysis in MCMCtree.

### Gene-tree/species-tree reconciliation

Modelling of gene DTL with ALE was used to assess the most likely root of embryophytes. We constructed a dataset comprising 24 genomes with the highest BUSCO completion for each lineage sampled (Supplementary Figs. 13 and 14 and Supplementary Table 13). An unrooted species tree was constructed using IQ-TREE under the LG + C60 + G4 + F model, as described in the ‘Phylogenetics’ section. The unrooted species tree was then manually rooted on 12 candidate branches, with each alternatively rooted tree scaled to geological time using the mean node ages from the dating analysis. Gene family clusters were inferred by an all-versus-all DIAMOND BLAST^86^ with an *e*-value threshold of 10^−5^, in combination with Markov clustering with an inflation parameter of 2.0 (ref. ^87^). All gene family clusters were aligned (MAFFT) and trimmed (BMGE), and bootstrap tree distributions were inferred using IQ-TREE as described above. Gene family clusters were reconciled under the 12 candidate root position trees using the ALEml algorithm^88^. The likelihood of each gene family under each root was calculated; the credible roots were determined using an AU test^89,90^. A detailed description of the ALE implementation can be found at https://github.com/ak-andromeda/ALE_methods/.

### Ancestral gene content reconstruction

Gene family clusters for the genomic dataset were inferred using the same methods as described above, but the dataset was expanded to contain the genomes of five algal outgroups to allow inference of gene content evolution prior to the embryophyte root (Supplementary Figs. 3 and 4). Ancestral gene content and instances of gene duplication, loss and transfer were determined by reconciling the gene family clusters with the rooted species tree under the ALEml model. We repeated the analyses using different approaches to filter the data for low-quality gene families (Supplementary Methods). A custom Python script called Ancestral_reconstruction_copy_number.py was used to identify the presence and absence of gene families on each branch of the tree from the ALE output (Supplementary Methods). To functionally annotate the gene families, we inferred the consensus sequence of each gene family alignment using hidden Markov modelling^91^. Consensus sequences were functionally annotated using eggNOG-mapper^92^, and GO terms were summarized using the custom Python script make_go_term_dictionary.py. For deeper nodes of the tree where GO terms were infrequent, genes were annotated with the KEGG database using BlastKOALA^93^. KEGG annotations were summarized using the Python script kegg_analysis.py. Additionally, the numbers of DTL events per branch were calculated using the custom Python script branchwise_number_of_events.py.

### Reporting summary

Further information on research design is available in the Nature Research Reporting Summary linked to this article.

## Supplementary information

Supplementary Information Supplementary Figs. 1–15 and Methods.

Reporting Summary

Supplementary Tables Supplementary Tables 1–14.

## Data Availability

All data are available on FigShare at 10.6084/m9.figshare.c.5682706.v1.

## Code availability

The scripts and code are available at https://github.com/ak-andromeda/ALE_methods/ and https://github.com/dschrempf/mcmc-date.
