# Supplementary material for: Divergent evolutionary trajectories of bryophytes and tracheophytes from a complex common ancestor of land plants
Source: Nat Ecol Evol. 2022 Sep 29;6(11):1634–43. doi: 10.1038/s41559-022-01885-x (PMC9630106; doi:10.1038/s41559-022-01885-x)
Supplement: Supplementary file 1 — Supplementary Figs. 1–15 and Methods. [file 41559_2022_1885_MOESM1_ESM.pdf]

nature ecology & evolution

Article https://doi.org/10.1038/s41559-022-01885-x

Divergent evolutionary trajectories of bryophytes and tracheophytes from a complex common ancestor of land plants

In the format provided by the authors and unedited

Supplementary Methods

1. Selecting gene families for phylogenetic analyses

Whole genome duplications are prevalent in embryophytes[52], leading to multiple gene copies and paralog-dominated gene families. As a result, single copy orthologous gene families used to construct supermatrices are difficult to identify – especially when sampling taxon-rich datasets. We employed OrthoFinder 2.0[67] to infer single copy orthologs from our dataset of 177 species of plants and algae. No single copy orthologous gene families were inferred. In previous studies single copy orthologous gene families have been manually curated[20]. We chose to develop a systematic algorithm identifying the gene families closest to single copy (Supplementary Fig. 21). The filtering method employed ensured only high-quality gene families comprised the final alignment – with only 2/177 species being represented by more than 50% gaps. The algorithm is split into two scripts *prem3.py* and *super_matrix_2.py* - prior to calling *super_matrix_2.py* the gene families need to be aligned and trimmed. This can be done prior to use, however, aligning all orthogroups prior to filtering would require considerable time and computational resources.

2. Transcriptome Assembly

Raw sequencing reads were downloaded from the Sequence Reads Archive (SRA) and trimmed using trimmomatic[95] under default settings to remove adapter sequences and trim low-quality bases. Transcriptomes were de novo assembled in Trinity[96]. Transcriptomes were filtered using the EvidentialGene pipeline[97] to remove isoforms and translate transcripts into amino acid sequences.

3. Root inference with Amalgamated Likelihood Estimation (ALE)

ALE models gene duplication, transfer and loss (DTL) to assess the likelihood of gene families under differing roots. Due to our phylogenetic dataset (Supplementary Table 12) containing both genomes and transcriptomes, the incomplete nature of the transcriptomes might be difficult to model accurately. In Supplementary Fig. 11, the fraction missing of each lineage is displayed (detailed in Supplementary Table 12); for many lineages, more than half of the BUSCOs are absent. As a result, we decided to curate a new dataset comprising only high-quality and more complete genomes. The dataset was comprised of 24 species, with each of the seven major lineages of embryophytes being represented by at least one genome (Supplementary Table 13). Where possible, we used "selected" proteome sets which have already been filtered to remove isoforms and splice variants. Otherwise, proteomes were filtered using CD-HIT with a cut-off of 0.99. 23 out of 24 species had more than 70% BUSCO completion, with most of the data being more than 80% complete. Moreover, the gene family clusters inferred from the high-quality dataset had greater species representation and thus might be more informative for inferring the root (Supplementary Fig. 13-14). There were also fewer large clusters with poor species representation in the high-quality dataset i.e., fewer gene families that have either undergone myriad duplications or had multiple isoforms of genes from sequencing anomalies. As a result, we only took forward the high-quality dataset for further analysis.

The high-quality gene families were analysed by ALEml – ALEml is time-consistent with the fossil-calibrated species tree and avoids time-inconsistent gene transfers (that is, gene transfers travelling back in time). We ran ALEml on 12 rooted and dated embryophyte trees, with an AU test suggesting three credible roots: hornworts, moss, and monophyletic bryophytes

(Supplementary Table 1). The credible roots were all in the same region of the tree, and the likelihood of the roots decreased with increasing distance from the root region. Moreover, analysis of the DTL signal (Supplementary Fig. 1), revealed that the signal was stable for this set of credible roots.

We also employed two additional techniques to test the root of embryophytes to ensure confidence in our topology for the reconstruction of ancestral states. Firstly, we inferred constrained maximum likelihood trees from the concatenation to be in accordance with the three credible roots selected by ALE i.e., hornworts were constrained to group with algae at the base of the tree, and thus branch from the root of embryophytes. The constrained trees were inferred in IQ-Tree[70], under the LG+C60+G4+F model. The likelihood of the three constrained trees was then assessed with an AU test[91] – rejecting both moss and hornworts as earliest diverging lineages of embryophytes (Supplementary Table 2). Secondly, we ran STRIDE[36], an alternative rooting software which employs duplication events to determine the root. STRIDE in combination with OrthoFinder 2.0[67], reconciled 18560 orthogroups, and suggested three most-parsimonious roots: between bryophytes and tracheophytes, on the hornwort stem and on the liverwort stem (Fig. 1). These results agree in part with the ALE analysis and the outgroup-rooting analyses, but propose an additional potential root on the liverwort stem. The support from the STRIDE analysis for the hornwort-stem root was weak (0.2% probability).

4. Ancestral gene content reconstruction

In addition to reconstructing the gene content of the ancestral embryophyte, we included several algal species that had highly complete genomes; the inclusion of algae allowed us to investigate gene content evolution in the closest algal relatives of embryophytes. The inclusion of algae also enabled us to investigate further the gene loss proposed to underpin the reduced body plan of the embryophyte sister lineage, Zygnematophyceae. In addition to algae, a subspecies of *Marchantia polymorpha (ruderalis*), was added to the dataset, allowing us to calculate values for DTL and copy number for the internal node defining liverworts. Single copy orthologs were determined using the same method as the main analysis (Supplementary Fig. 15) – 185 single copy gene families were inferred, leading to an alignment of 71855 sites. A maximum likelihood tree (Supplementary Fig. 3) was inferred from the dataset under the LG+C60+G+F model in IQ-Tree[70]. The tree was rooted in accordance with the previous analysis. Again, even with this reduced dataset, we recover monophyletic bryophytes with high support (97% Bootstrap support). Due to ALEml requiring a dated tree for reconstruction of ancestral gene content, a time scale was inferred using a subset of the calibrations from the transcriptomic analysis (Supplementary Fig. 4). Divergence time analyses were performed as described for the transcriptomic analysis.

Gene family clusters were inferred using the same methods as the rooting analysis. The pipeline yielded 20822 gene family clusters with 1000 bootstrap samples for each family. The gene families were reconciled with the rooted species tree, using the ALEml algorithm with the fraction missing of each genome also incorporated. The presence and absence of each gene family across the tree was inferred using the *Ancestral_reconstruction_copy_number.py* python script – we determined that a gene family was present if it had more than 0.5 copies at the specified branch i.e., was present at the branch in more than 50% of the reconciliations. The summed number of DTL evolutionary events was determined with *branchwise_number_of_events.py*. In addition to this, we scaled the number of events by the branch length of the time scale analysis, to determine the rate of DTL for important sections of the tree. The number of DTL events and the summed gene copy number for each branch of the tree were calculated (Supplementary Table 3). The presence and absence of each gene family

cluster across the embryophyte tree, and whether an *Arabidopsis* gene is present in said cluster is displayed in Supplementary Table 4.

Clustering proteomes into gene families remains a high-throughput and potentially inaccurate process. To assess the robustness of our results to poorly-clustered or erroneous gene families, we undertook several filtering analyses. Gene families where the total trimmed alignment length was short may represent poorly designated gene families and so we progressively removed families with short alignments (>70, 60, 50 and 30 amino acids). and repeated the analysis of duplications, losses and transfers. We found that filtering by alignment length did result in fewer observed losses, but this was consistent across clades and so the losses observed in bryophytes are not the result of poorly aligned gene families. Very large gene families may also represent gene families where the clustering algorithm has failed to distinguish individual gene families, and so we progressively removed the top 1, 2.5, 5 and 10% gene families by size. We found that removing the largest gene families had a large effect on the number of inferred losses, especially among bryophytes, and that removing the largest 10% of gene families results in more inferred losses in tracheophytes than in bryophytes. This suggests that the losses in bryophytes are primarily occurring in the largest gene families. In a parsimony approach, loss in bryophytes would typically require a gene to also be present in the algal outgroups. This is not the case in a probabilistic framework with a single loss rate applied across the gene family, where high rates of loss in one part of the tree can drive losses in other regions of the tree. We repeated our analyses with a subset of gene families that contain at least one copy in one of the algal outgroups. We found that while this did decrease the number of losses along the bryophyte stem, it did so proportionally across both bryophytes and tracheophytes. Finally, high rates of duplication, transfer and loss may suggest that a gene family is poorly clustered or that its evolutionary history is poorly resolved. We repeated our analyses while progressively removing the top 1, 2.5, 5 and 10% of gene families by duplication, loss and transfer rate. We found that filtering by duplication and transfer rate had only a very small effect on the inferred number of losses, while filtering by loss rate unsurprisingly had a large effect. The number of losses decreased most in bryophytes, suggesting that the gene families with a high loss rate have disproportionately more losses in bryophytes.

To functionally annotate the gene families, the consensus sequence of each gene family was determined using HMMER3[92]. This process summarises the gene family into a single sequence most indicative of the ancestral state. The consensus sequences were functionally annotated using eggNOG-mapper 2[93]. The gene families present at each branch were determined as described above, and the functional annotation for their corresponding consensus sequence was retrieved. The frequency of GO terms associated with the consensus sequences present at each branch was calculated using the custom python script *make_go_term_dictionary.py.* In doing so, the change in the frequency of GO terms could be tracked from node to node, allowing us to identify changes in function across the embryophyte tree. Changes in the frequency of GO terms from the embryophyte ancestor to bryophytes, and to tracheophytes were calculated – this is termed the GO term differential i.e., if there was a frequency of 10 for the GO term "Shoot Development" at the ancestral embryophyte branch, but only a frequency of 6 for the bryophyte branch, the GO term differential would be -4. The GO term differentials are presented in Extended Data Fig. 5: on the left-hand side, all GO term differentials are plotted, i.e., the change in every GO term differential between the ancestral branch and the subsequent tracheophyte and bryophyte branch is plotted. Overall, there has been a reduction in the frequency of GO terms in bryophytes in comparison to the ancestral embryophyte, suggesting there has been a wide-scale genomic reduction in this lineage. This was also suggested by the analysis of DTL rates which suggested a vast amount of loss on this branch. On the right, changes in select GO terms/functional processes are highlighted.

Reduction in the frequency of GO terms associated with reproductive shoot and vegetative shoot development are observed between the ancestral embryophyte and the bryophytes, whilst the inverse is true for tracheophytes (Supplementary Tables 5, 7 and 9).

One limitation of GO term analysis is that differences on the bryophyte and tracheophyte stems might reflect, in part, differences in the completeness of functional annotation for the extant members of the two clades. To investigate the broad-scale patterns identified in the GO term analysis in more detail, we investigated the evolutionary histories of 50 key genes involved in *Arabidopsis* stomatal function/development (Supplementary Table 10), vasculature development and symbiosis/defence (Supplementary Fig. 9-10). We were able to determine where the gene families that these genes belong to originated, were present, and were lost across the tree. Many of the orthologous gene clusters that harbour critical constituents of the stomatal development pathway such as SPCH/MUTE/FAMA, TMM, and ERECTA originated on the ancestral embryophyte branch, suggesting that stomata evolved at this point. Moreover, orthologous gene families of master-regulators of stomatal function such as HT1 and OST1 were also present. In addition to this, gene families which contain key *Arabidopsis* vasculature genes such as WOX4 and WER (MYB66) originated on the branch defining Embryophytes – highlighting a possible increase in vasculature complexity. Interestingly, our analysis suggests that these gene families were lost on the branch defining bryophytes (Supplementary Fig. 9-10), suggesting a reduction in vasculature complexity in this lineage.

To further assess the losses of important developmental genes on the branch defining bryophytes, manually curated phylogenetic trees were constructed for their respective gene families (Extended Data Fig. 6). The gene trees were inferred by first undertaking an OrthoFinder analysis of an expanded dataset, comprising further representatives of each land plant lineage. The homologues were identified within orthogroups and then aligned with MAFFT (--globalpair) and trimmed with BMGE. For each alignment, a phylogenetic tree was inferred in IQ-Tree under the best fitting model determined by BIC. The trees were then rooted in accordance with the previous analysis (Fig. 1). For the representative genes, a pattern of loss in bryophytes and concurrent expansion via duplication in tracheophytes is observed. Additionally, we see wide scale loss of gene families which harbour critical stomata and vasculature associated genes on the branches that define hornworts and liverworts (Supplementary Fig. 9-10). The loss of stomatal-associated gene families in these lineages is congruent with the findings of Harris *et al.,* (2020). Presently, the number of available genomes for non-flowering plants is limited and is a potential source of bias for estimating ancestral genome content in deeper nodes of the tree. We expect, with the increasing volume of sequence data and projects specifically targeting non-flowering plants (e.g. 10000 genome project, Sphagnum genome project), that it will be possible to develop a still more detailed reconstruction of the ancestral embryophyte.

5. Molecular clock estimates of the timing of land plant evolution

The age of land plants has become a recent controversy, with some studies favouring a far older (pre-Cambrian) origin of crown embryophytes [23,40,41], while others limit the origin of land plants to the late Cambrian. These differences reflect diverging approaches to the implementation of maximum age constraints derived from the fossil record. Previous studies have employed a maximum age derived from the earliest record of cryptospores. Cryptospores are spores that possess certain characteristics of embryophyte spores, but are sufficiently different to make their assignment to any extant clade difficult. However, the possibility of an embryophyte affinity cannot be disregarded. Cryptospores possess a similarly high preservation potential to the spores of extant land plants, and indeed from the Ordovician

onwards there is a good fossil record of spores that are assigned to extant land plant lineages. This provides the basis of the maximum age calibration, following the reasoning that if land plant spores did exist prior to the Cambrian, they would likely be preserved.

To address recent discussions about the value of these maxima, we implemented two additional calibration strategies. In the first (Strategy B), we relaxed the late Cambrian maximum age calibration on crown embryophytes (and all internal nodes that share the same calibration). This was achieved by setting an arbitrary ancient maxima of 1 Ga. The maximum age on tracheophytes (derived from similar reasoning regarding the presence of trilete spores), was retained. In the second strategy, we relaxed the maximum calibration on tracheophytes also to 1 Ga, providing no effective maxima on the major lineages of land plants. Analyses were performed as described in the methods of the main text and the impact on the age of crown embryophytes across calibration strategies is compared in Extended Data Fig. 4.

The absence of deep fossil calibrations in hornworts has led to greater uncertainty in the estimates of their origin. We employed a novel relative constraint based on the horizontal gene transfer of the NEOCHROME gene from hornworts into ferns to force hornworts to be older than certain fern lineages. Topological uncertainty in the NEOCHROME gene tree suggests multiple potential placements of the gene transfer, with the most probable being into the ancestor of Polypodiales. We accounted for this uncertainty by repeating the analyses with multiple relative constraints, forcing hornworts to be older than Polypodiales, Cyatheales and Gleicheniales, all of which variously contain copies of the NEOCHROME gene. Forcing hornworts to be older than the more ancient lineages (Cyatheales and Gleicheniales) resulted in more ancient estimates of the age of crown-group hornworts, including the possibility an Ordovician origin (Extended Data Fig. 3).

6. Fossil Calibrations for Molecular Clock Analyses

C_1: MRCA Viridiplantae/Chloroplastida

Fossil Taxon and Specimen: *Proterocladus antiquus* [VPIGM-4762, deposited at Virginia Polytechnic Institute Geoscience Museum], from the lower Nanfen Formation in North China [1].
Phylogenetic justification: Macrofossils from the Nanfen Formation, Northern China, have reconstructed an erect, epibenthic, multicellular alga. Tang *et al.* [1] argue that the overall morphology of *P. antiquus* is comparable to extant members of Ulvophyceae, yet no diagnostic features are described. As such, *P. antiquus* can conservatively be used to provide a minimum calibration on the origin of Viridiplantae.
Minimum age: 940.4 Ma
Maximum age: 1891 Ma
Age justification: The Nanfen Formation is positioned within the Xihe group in southern Liaoning Province, North China [2]. The Nanfeng Formation has not been dated directly. The age of the underlying Diaoyutai Formation can only be constrained by detrital zircons, the youngest population of which has been dated to 1056 Ma ± 22 Ma [3]. A sill in the overlying Qiaotou Formation, which overlies the Nanfeng Formation, has a zircon secondary-ion mass spectrometry U-Pb age of 947.8 Ma ± 7.4 Myr [4]. Thus, the minimum age of the Nanfeng Formation can be established on the minimum age interpretation of this sill viz. 940.4 Ma, although it is presumably significantly older. The soft maximum age is established following Morris *et al.* [5] and is based on the earliest record of eukaryotes, where despite the presence of simple eukaryotes, there is no evidence of complex or multicellular eukaryotes such as green algae.

Discussion: *Proterocladus* is determined as a crown group member of green algae based on the cellular differentiation, presence of a holdfast, inferred siphonocladous nature, and lack of an outer sheath. The absence of an outer sheath and siphonocladous nature precludes an affinity with stigonematalean cyanobacteria. A fungal affinity was also rejected based on the structure of the septae, lack of mycelium-like branching and the structure of the reproductive organs. The septation in *Proterocladus* also suggests that it is not similar to the xanthophycean alga *Vaucheria* or the rhodophyte *Griffithsia*. Nie *et al.* consider a Mesoproterozoic specimen identified as *Pterospermella* as a member of Prasinophyceae [6, 7], however the lack of clearly described structures prevents their unequivocal assignment to the green algae [5].

C_2: Chlorophyceae – Prasinophyaceae

Fossil taxon and specimen: *Palaeocymopolia silurica*
Phylogenetic justification: Following Morris *et al.* [5]
Minimum age: 438.3 Ma
Maximum age: 1891 Ma
Age justification: Following Morris *et al.* [5]

C_3: Steptrophyta: "Streptophytes" - Embryophytes

Fossil taxon and specimen: *Tetrahedraletes cf. medinensis*
Phylogenetic justification: Following Morris *et al.*, [5]
Minimum age: 469 Ma
Maximum age: 1891 Ma
Age justification: Following Morris *et al.* [5]

C_4: Embryophyta: Bryophyta – Tracheophyta

Fossil taxon and specimen: *Cooksonia cf. pertoni*
Phylogenetic justification: Following Morris *et al.* [5]
Minimum age: 426.9 Ma
Maximum age: 515.5 Ma
Age justification: Following Morris *et al.* [5]

C_5: Marchantiophyta: Jungermanniopsida - Marchantiopsida

Fossil taxon and specimen: *Metzergiothallus sharonae* [NYSM 17656, Paleontology Collection of the New York State Museum, Albany, USA], from the Sample CHD-4.6, Plattekill Formation of the eastern Hamilton Group, Givetian (late Middle Devonian) at Cairo Highway Department quarry, just south of New York State Route 145, 3.22 km NW of Cairo, Greene County, New York (42.32° N and 74.04°W, NAD 83).
Phylogenetic justification: Morris *et al.* [5] identified *Riccardiothallus devonicus* as the oldest liverwort but its compression-preservation precludes the identification of anatomical characters that might corroborate a liverwort affinity beyond its general form [8]. *Metzergiothallus sharonae,* preserves anatomical detail including putative oil body cells [9, 10], bifurcating thalloid habit with an axial costa, dorso-ventral cellular differentiation, ventral ribbon-like rhizomes, and associated sporophyte that has a multivalve structure [11]. These characteristics support the interpretation of *Metzergiothallus sharonae* as a crown group liverwort, as supported by a cladistic analysis of morphological characters [12]. Flores *et al.* placed *M. sharonae* as sister to the extant genus *Metzgeria*, albeit with very low bootstrap support (50%).

As such, we conservatively consider *M. sharonae* as a member of the Jungermanniopsida and thus capable of calibrating the divergence between Jungermanniopsida and Marchantiopsida.
Minimum age: 377.7 Ma
Maximum age: 515.5 Ma
Age justification*:* Attempts to resolve the age of the Plattekill Formation at Cairo Quarry through palynostratigraphy have proven unsuccessful due to poor preservation [13], however, the age of the Plattekill Formation has been established elsewhere. Willis and Bridge [14] placed the Plattekill and overlying Manorkill Formations in the mid *lemurata magnificus*–mid *optivus triangulatus* zones of Richardson and McGregor [15], indicating a latest Eifelian-early Givetian age [16]. In the absence of better stratigraphic constraint, this allows us to establish a minimum constraint on the age of the Plattekill Formation based on the Givetian-Frasnian boundary, dated to 378.9 Ma ± 1.2 Myr [16], thus 381.7 Ma.

C_6: Anthocerotophyta: Anthocerotaceae

Fossil taxon and specimen: Anthoceros sp. (spore type A) [BA Pb Pm 5034, Museo Argentino de Ciencias Naturales] from the Albian Anfiteatro de Ticó Formation, Baqueró Group, Santa Cruz Province, Argentina
Phylogenetic justification: This spore was assigned to the extant genus *Anthoceros* based on the similar morphology [17, 18].
Minimum age: 116.59 Ma
Maximum age: 515 Ma
Age justification: The spores were recovered from the Anfiteatro de Ticó Formation [18], which is overlain by the Bajo Tigre Formation, constrained based on U/Pb dating to 116.85 ± 0.26 Ma [19]. Thus, a minimum age of 116.59 Ma is proposed.

C_7: Anthocerotophyta: Notothyladaceae

Fossil taxon and specimen: Notothylites nirulai from the Deccan Intertrappean beds of Mohgaon-Kalan, Madhya Pradesh, India [20].
Phylogenetic justification: The overall morphology has a similar thallus size, sporophyte size and elater shape to extant *Notothylas*, as well as lacking stomata on the sporophyte [17].
Minimum age: 65.81 Ma
Maximum age: 515 Ma
Age justification: The age of the Intertrappean beds at Mohgaon-Kalan was determined to be Maastrichtian on the palynological assemblage being characterised as Maastrichtian and the magnetostratigraphy indicating the flows to be of C30N (Maastrichtian) [21]. Thus, a minimum age based on the base of the C30N polarity chron of 66.31 ± 0.5 is proposed [22].

C_8: Anthocerotophyta: *Phaeomegaceros*
Fossil taxon and specimen: Phaeoceros sp. from the Uscari Formation, Costa Rica
Phylogenetic Justification: Though originally assigned to the extant genus *Phaeoceros [23]*, the spore is highly similar to the extant genus *Phaeomegaceros* in size and morphology (Villareal and Renner)
Minimum age: 13.82
Maximum age: 515 Ma
Age justification: Material was deposited in the Uscari Formation in the Limon Basin of Costa Rica. Analysis of planktonic formanifera identified an overlap of *Orbulina universa* and *Globorotalia foshi peripheroronda*, placing the the Formation in the early Middle Miocene (formaniferal zones N9 to N10) [24]. Following Hilgen et al. [25], the base of the N10

formaniferal zone correlates with the M7 zone and the base of the Langhian and thus a minimum age of 13.82 Ma is derived.

C_9: Marchantiophyta: Pelliidae - [Jungermannidae + Metzgeriidae]

Fossil taxon and specimen: *Pellites hamiensis* [SDL-98-4-91, Institute of Paleontology and Stratigraphy of Lanzhou University, Gansu Province, China] from the Sandaoling coal mine, Hami City, Xinjiang Uygur Autonomous Region, China.
Phylogenetic justification: The gross morphology and anatomy of the thalli support a placement within Pelliaceae [26], including ribbon-like segments, thinning wings, a unistratose margin, a conspicuous costa with elongate cells and thick rectangular cells near the margin. The absence of reproductive structures led the authors to assign the samples to the fossil genus *Pellites* rather than *Pellia* [27].
Minimum age: 167.0 Ma
Maximum age: 515.5 Ma
Age justification: Material was collected from the Xishanyao Formation of the Sandaoling coal mine, which overlies the lower Jurassic Sangonghe Formation and underlies the Palaeogene Shansan Group strata [28]. Based on biostratigraphy and plant assemblages, an Aalenian to Bajocian age is proposed for the Xishanyao formation [29]. Thus, we propose a minimum age according to the upper boundary of the Bajocian, 168.2 ± 1.2 Ma [30].

C_10: Marchantiophyta: Sphaerocarpales – Marchantiales

Fossil taxon and specimen: *Marchantites cyathodoides*
Phylogenetic justification: Following Morris *et al.* [5]
Minimum age: 227.6 Ma
Maximum age: 515.5 Ma
Age justification*:* Following Morris *et al.* [5]

C_11: Marchantiophyta: Ricciaceae – Conocephalaceae

Fossil taxon and specimen: *Ricciopsis ferganica* [FG 596/X/721 in the Palaeontological collection of the Geologisches Institut, Technische Universität Bergakademie Freiberg, Germany] from the village of Madygen, southwest Kyrgyzstan, Central Asia
Phylogenetic justification: *R. ferganica* was assigned to the fossil genus *Ricciopsis* (Ricciaceae) [31] based on resemblance to the thalloid floating forms of the extant genus *Riccia.* No diagnostic features are available for the genus, however a flattened, small branching thallus and the presence of a longitudinal median furrow or groove on the dorsal side of the thallus is characteristic of Ricciaceae [32]. Further, a cladistic analysis of morphological characters supported the placement of *Ricciopsis* as sister to the extant genus *Riccia* [12].
Minimum age: 227.6
Maximum age: 515.5 Ma
Age justification*:* Material was collected from the lower and middle parts of the uppermost lacustrine unit at Urochishche Madygen. The Madygen Lagerstätten is deemed to be Ladinian-Carnian (Middle to Late Triassic) in age based on comparative studies of the flora [33] and insect assemblages [34]. Thus, we propose a minimum age based on the top of the Carnian of 227.3 ± 0.1 Ma [30].

C_12: Marchantiophyta: Jungermanniidae – Metzgeriidae

Fossil taxon and specimen: *Cheirorhiza brittae* [515-38a, Far East Geological Institute, Far East Branch of the Russian Academy of Science] from the Bureja basin, Russia
Phylogenetic justification: This genus has complicate-bilobed, subtransversely inserted leaves, massive stems with a small ventral merophyte and narrow, elongated cortical cells, as well as scattered, unbranched rhizoids
Phylogenetic justification: *C. brittae* has been compared to extant members of both Jungermanniales and Porelliales on the basis of morphology [35, 36], and so we follow Heinrichs et al. [37] and treat it as a stem lineage member of Jungermanniidae.
Minimum age: 143.1
Maximum age: 515.5
Age justification: Material was collected from the Talyndzhan formation of the Bureja basin. The Talyndzhan formation was initially determined to be of Callovian or Callovian-Oxfordian age based on floral assemblage. This has been revised in light of palynological evidence and the top of the Talyndzhan is believed to correspond to the Jurassic-Cretaceous boundary [38, 39]. Thus, a minimum age based on the upper boundary of the Jurassic 143.1 [30], and so 144.9 Ma, is proposed.

C_13: Marchantiophyta: Jungermanniales – Porellales

Fossil taxon and specimen: *Diettertia montanensis* [no. 281 of the University of Montana Paleontological Collection (UMPC)] from the Missouri river, Montana, USA.
Phylogenetic justification: This genus has complicate-bilobed, subtransversely inserted leaves, massive stems with a small ventral merophyte and narrow, elongated cortical cells, as well as scattered, unbranched rhizoids
Phylogenetic justification: *Dietteria montanensis* has been assigned as a member of Jungermanniales stem lineage based on its overall morphology and anatomy, including complicate-bilobed, subtransverely inserted leaves, stems with a small merophyte with narrow elongated cortical cells [37, 40].
Minimum age: 112.8 Ma
Maximum age: 515.5 Ma
***Age justification*:** Material was collected from the Kootenai Formation, Great Falls, Montana. Fossil assemblages, particularly molluscs, ostracods and charophyte algae indicate an Aptian age for the formation and an Albian age for the overlying Blackleaf formation [41, 42]. Thus, a minimum age is derived from the Aptian-Albian boundary, estimated at 113.10 ± 0.3 Ma, of 112.8 Ma.

C_14: Marchantiophyta: Porellineae – [Radulineae + Jubulineae

Fossil taxon and specimen: *Gackstroemia cretacea* [AMNHBU ASJH-1, American Museum of Natural History, New York], from Myanmar [43].
Phylogenetic justification: Heinrichs *et al.* [43] placed *G. cretacea* within the Lepidolaenaceae based on a sterile gametophyte, including incubous foliation, complicate bilobed leaves, splitting to a dorsal lobe and a ventral *Frullania*-type water sac. Based on the presence of bifurcate underleaves, the fossil was assigned to the extant genus *Gackstroemia.* Lepidolaenaceae are placed within the Porellineae, and so *G. cretacea* can calibrate the divergence of *Porella* (Porellineae) from *Radula* (Radulineae) and Frullania (Jubulineae).
Minimum age: 98.17

Maximum age: 515.5 Ma
Age justification: The age of Burmese amber has long been contested, with estimates ranging between the Miocene and Cretaceous. Claims of an upper Albian age based on ammonite biostratigraphy [44] have not been verified [45]. However, a U-Pb age of 98.79 Ma ± 0.62 Myr was obtained from zircons in volcanoclastics deposited contemporaenously with the amber [46]. Shi et al. (2012) and Yu et al. (2019) interpret this as a minimum age for the amber since it is bioeroded. Nevertheless, in the absence of other evidence, this U-Pb zircon age can be used to established a minimum constraint of 98.17 Ma [44].

C_15: Marchantiophyta: Radulaceae – [Frullaniaceae – Lejeuneaceae]

Fossil taxon and specimen: *Radula cretacea* [PB22484, Nanjing Institute of Geology and Palaeontology, Chinese Academy of Sciences, Nanjing], from amber localities near the village of Tanai, Kachin State, Myanmar [47].
Phylogenetic justification: The specimen was assigned to the genus *Radula* (Radulaceae, Porellales) based acute to acuminate leaf apices, gemmae exclusively produced from leaf-lobe marginal cells and female bracts present in two pairs [47].
Minimum age: 98.17
Maximum age: 515.5 Ma
Age justification: The age of Burmese amber has long been contested, with estimates ranging between the Miocene and Cretaceous. Claims of an upper Albian age based on ammonite biostratigraphy [44] have not been verified [45]. However, a U-Pb age of 98.79 Ma ± 0.62 Myr was obtained from zircons in volcanoclastics deposited contemporaenously with the amber [46]. Shi et al. (2012) and Yu et al. (2019) interpret this as a minimum age for the amber since it is bioeroded. Nevertheless, in the absence of other evidence, this U-Pb zircon age can be used to established a minimum constraint of 98.17 Ma [44].

C_16: Marchantiophyta: Frullaniaceae – Lejeuneaceae

Fossil taxon and specimen: *Frullania cretacea* [AMNH B-011, American Museum of Natural History, New York], from Myanmar [48]
Phylogenetic justification: *F. cretacea* was initially placed in the extant genus *Frullania* based on the reddish leaf colour, leaf cells with a single mammilla, lateral branching and helmet-shaped water sacs [49]. More recent findings support this placement, with the identification of a stylus shape similar to extant *Frullania,* bilobed underleaves and more elongate and toothed subgynoecial underleaves [48].
Minimum age: 98.17
Maximum age: 515.5 Ma
Age justification: The age of Burmese amber has long been contested, with estimates ranging between the Miocene and Cretaceous. Claims of an upper Albian age based on ammonite biostratigraphy [44] have not been verified [45]. However, a U-Pb age of 98.79 Ma ± 0.62 Myr was obtained from zircons in volcanoclastics deposited contemporaenously with the amber [46]. Shi et al. (2012) and Yu et al. (2019) interpret this as a minimum age for the amber since it is bioeroded. Nevertheless, in the absence of other evidence, this U-Pb zircon age can be used to established a minimum constraint of 98.17 Ma [44].

C_17: Bryophyta: Sphagnopsida – [Polytrichopsida – Bryopsida]

Fossil taxon and specimen: Sphagnales
Phylogenetic justification: Following Morris *et al.* [5]

Minimum age: 330.7 Ma
Maximum age: 515.5 Ma
Age justification*:* Following Morris *et al.* [5]

C_18: Bryophyta: Polytrichopsida – Bryopsida

Fossil taxon and specimen: *Palaeocampylopus buragoase*
Phylogenetic justification: Following Morris *et al.* [5]
Minimum age: 271.8 Ma
Maximum age: 515.5 Ma
Age justification*:* Following Morris *et al.* [5]

C_19: Bryophyta: Polytrichaceae – Tetraphidaceae

Fossil Taxon and Specimen: *Meantoinea alophosioides* [Gemmiferous gametophyte shoot in rock slab UAPC-ALTA P15393 B (slides B bot series a), University of Alberta Paleobotanical Collections (UAPC-ALTA), Edmonton, Alberta, Canada] from the Lower Cretaceous deposits at Apple Bay (Vancouver Island, British Columbia, Canada) [50].
Phylogenetic Justification: Bippus *et al.* [50] placed *M. alophosioides* within the extant family Polytrichaceae based on the presence of leaves with diagnostic anatomy, differentiated into sheathing base and free lamina, bearing photosynthetic lamellae, and a conducting strand present in the stem. A phylogenetic analysis of 91 morphological characters confirmed the position of *M. alophosioides* as a stem group member of Polytrichaceae [51].
Minimum age: 132 Ma
Maximum age: 515.5 Ma
Age justification: Sediments of the Apple Bay locality are regarded as Longarm Formation equivalent and originally proposed as Barremian based on the presence of monosulcate reticulate pollen grains [52]. Klymiuck and Stockey [53] argued that these grains are more likely associated with extinct gymnosperms, and instead propose a Valanginian age, supported by oxygen isotope analysis (D. Gröcke, personal communication, 2005, 2013). Thus, we propose the upper boundary of the Valanginian 132.6 ± 0.6 Ma and thus, 132 Ma as a minimum age constraint.

C_20: Bryophyta: *Atrichum – Polytrichum*

Fossil Taxon and Specimen: *Eopolytrichum antiquum* [PP44714, Department of Geology, Field Museum, Chicago] from Buffalo Creek member of the Gaillard Formation (Upper Cretaceous), Crawford County, Georgia, USA [54].
Phylogenetic Justification: Konopka et al. [54] determined that *Eopolytrichum* was a member of the extant family Polytrichaceae based on several features including the epiphragm, broadened columella apex, peristomial membrane structure, and echinulate spore sculpture, as well as the overall lamellae and leaf anatomy associated with Polytrichaceae. Based on a cladistic analysis of 99 characters Bippus et al. [51] recovered *Eopolytrichum* nested within the extant genus *Polytrichum.*
Minimum age*:* 82.9 Ma
Maximum age: 515.5 Ma
Age justification: *Eopolytrichum* was recovered from the sediments of the Allon quarry, assigned to the Buffalo Creek Member of the Gaillard Formation. Based on palynological analyses, a Campanian age was initially inferred [55]. However, this was later revised to a late Santonian age based on the correlation of the Allon locality palynological assemblage and that

of the Coastal Plain Province [56], which was assigned to Burnett's calcareous nannofossil zone CC17 [57], the base of which correlates with the Santonian/Campanian boundary. Thus, a minimum age constraint of 83.6 ± 0.7 is proposed.

C_21: Bryophyta: Funariidae – [Dicraniidae + Bryidae]

Fossil taxon and specimen: *Capimirinus riopretensis*
Phylogenetic justification: Following Morris *et al.* [5]
Minimum age: 268.3 Ma
Maximum age: 515.5 Ma
Age justification: Following Morris *et al.* [5]

C_22: Bryophyta: Dicraniidae – Bryiidae

Fossil Taxon and Specimen: *Tricarinella crassiphylla* [Gametophyte shoot in rock slab UAPC-ALTA P13311, University of Alberta Paleobotanical Collections, Edmonton, Alberta, Canada], from the Apple Bay locality, Longarm Formation equivalent, Quatsino Sound, northern Vancouver Island, British Columbia, Canada (latitude 50°36′21″N, 127°39′25″W; UTM 9U WG 951068) [58].
Phylogenetic justification: *Tricarinella crassiphylla* is distinguished predominantly based on 3 characters: tristichous phyllotaxis, bistratose leaf lamina and a homogeneous costa. This set of characters, along with the clear implication that *T. crassiphylla* was an acrocarpous species, leads to its placement within Grimmiales [58]. Grimmiales + Dicranales form part of the Dicraniidae, which is in turn sister to Bryiidae, and so was used to establish a minimum age constraint on the divergence of *Ceratodon* (Dicranales) and Bryiidae.
Minimum age: 133.3 Ma
Maximum age: 515.5 Ma
Age justification: Sediments of the Apple Bay locality are regarded as Longarm Formation equivalent and orginially proposed as Barremian based on the presence of monosulcate reticulate pollen grains [52]. Klymiuck and Stockey [53] argued that these grains are more likely associated with extinct gymnosperms, and instead propose a Valanginian age, supported by oxygen isotope analysis (D. Gröcke, personal communication, 2005, 2013). Thus, we propose the upper boundary of the Valanginian 133.9 ± 0.6 Ma and thus, 133.3 Ma as a minimum age constraint.

C_23: Bryophyta: Hypnanae - Brynanae

Fossil taxon and specimen: *Krassiloviella limbelloides* [Gametophyte shoot in rock slabs UAPC-ALTA P17596 C bot and D, University of Alberta Paleobotanical Collections (UAPC-ALTA), Edmonton, Alberta, Canada] from the northern shore of Apple Bay, Quatsino Sound, on the west side of Vancouver Island, British Columbia, Canada (lat. 507360 2100N, long. 1277390 2500W; UTM 9U WG 951068) [59].
Phylogenetic justification: *K. limbelloides* was described as a pleurocarpous moss based on the presence of pinnate branching, homocostate and pluricostate leaves [59]. This was further supported by features including a monopodial main stem, second-order branching, the lack of conducting strands in the stems, and thin laminal cell walls. This combination of features indicates membership within the superorder Hypnanae, and the extinct family Tricostaceae.
Minimum age: 132 Ma
Maximum age: 515.5 Ma

Age justification: Sediments of the Apple Bay locality are regarded as Longarm Formation equivalent and orginially proposed as Barremian based on the presence of monosulcate reticulate pollen grains [52]. Klymiuck and Stockey [53] argued that these grains are more likely associated with extinct gymnosperms, and instead propose a Valanginian age, supported by oxygen isotope analysis (D. Gröcke, personal communication, 2005, 2013). Thus, we propose the upper boundary of the Valanginian 132.6 ± 0.6 Ma and thus, 132 Ma as a minimum age constraint.

C_24: Tracheophyta: Lycopodiophyta – Euphyllophyta
Fossil taxon and specimen: *Zosterophyllum sp.* and *Chelinospora? sp.* from the Ordovician successions of central Sweden.
Phylogenetic justification: The minimum age is derived following Morris *et al.* [5].
Minimum age: 420.7 Ma
Maximum age: 458.88 Ma
Age justification*:* The minimum age is derived following following Morris *et al*. [5]. The soft maximum is based on a probably trilete spore (*Chelinospora? sp.*) from the Ordovician successions of central Sweden [60]. Spores were sampled from the Borenshult-1 drillcore, comprising a well-dated succession of the Middle to Upper Ordovician. The successions are dated based on conodont biostratigraphy [61] and occur below the Kinnekulle K-bentonite, itself dated using U/Pb zircons to 454.41 ± 0.17 Ma [62, 63], confirming a Sandbian age. The spore was sampled from 48.2m, corresponding to the *Baltoniodus variabilis* biozone, with a maximum age corresponding to the top of the Sandbian, 458.18 ± 0.7 Ma [61, 64].

C_25: Lycopodiophyta: Isoetales – Lycopodiales

Fossil taxon and specimen: *Leclercqia complexa*
Phylogenetic justification: Following Morris *et al.* [5]
Minimum age: 392.1 Ma
Maximum age: 451 Ma
Age justification*:* Following Morris *et al*. [5]

C_26: Lycopodiophyta: Isoetales – Selaginellaes

Fossil taxon and specimen: *Yuguangia ordinata* [Specimen BUP.H-y07, Department of Geology of Peking University, PRC], from the Haikou Formation of Yuguang village, Zhanyi District, Yunnan Province, southwestern China [65].
Phylogenetic justification: *Yuguangia* is placed within Lycophytina based on its overall morphology, including external and internal anatomy and reproductive structures. A cladistic analysis by Hao *et al.* [65] based on 19 morphological characters placed *Yuguangia* as more closely related to the extant order Isoetales than Selaginellales, and thus it allows a minimum age constraint on the divergence of *Isoetes* and *Selaginella.*
Minimum age: 377.7 Ma
Maximum age: 451 Ma
Age justification: A Givetian age for the Haikou Formation was determined based on the presence of a miospore assemblage of *Cirratriradites monogrammos, Archaeozonotriletes variabilis*, *Cymbosporites magnificus* rm **Geminospora lemurata* [66]. Thus, we propose a minimum age constraint coinciding with the upper boundary of the Givetian (388.9 ± 1.2 Ma)

C_27: Lycopodiophyta: *Selaginella selaginoides* – [rhizophoric *Selaginella*]

Fossil Taxon and Specimen: *Selaginella resimus* [BMNH V.62303-V.62330, Palaeontology Department, Natural History Museum, London] from the Puddlebrook assemblage (Lower Carboniferous), Drybrook Sandstone of the Forest of Dean, Gloucestershire [67].
Phylogenetic Justification: Rowe [67] described small lycophyte leafy shoots with consistently narrow stems and terminal strobili, placing it within *Selaginella.* The leaves are isophyllous and decussately arranged, supporting a placement within the subgenus *Boreoselaginella.* Thus, *S. resimus* provides a minimum age for the divergence of *S. selaginoides* and the rhizophoric clade, following Westrand and Korall [68, 69].
Minimum age: 322.8 Ma
Maximum age: 451 Ma
Age Justification: The plant-bearing outcrop of the Puddlebrook assembly is part of the Drybrook Sandstone at Plump Hill. The correlation between Puddlebrook and Plump Hill is supported by macrofossil remains of the lycophyte *Lepidostrobophyllum fimbriatum* and the pteridosperm *Diplopteridium*, both commonly occurring at Puddlebrook [70, 71]. Plump Hill is characterised by the miospores *Perotriletes tesselatus, Schultzospora* and *Carbaneuletes circularis,* indicative of an Asbian/Holkerian age. An Upper Visean age for the Puddlebrook assembly is further supported by the presence *Tetrapterites visensis,* which is known from the Upper Visean of Carboniferous limestome of the Menai region of Wales, and the presence of *Cribosporites cribellatus* [72],which supports and Asbian level. Following Poty *et al.* [73], the Asbian correlates with the Chesterian and the Warnantian, which both share an upper boundary with the Serpukhovian (323.2 Ma ± 0.4) and thus a minimum age of 322.8 Ma is proposed.

C_28: Lycopodiophyta: *Selaginella subg. Stachygynandrum – Selaginella kraussiana*

Fossil taxon and specimen: *Selaginella sp.* **[**GZG.BST.21966-2002, Geoscience Centre, University of Göttingen] from the mid-Cretaceous Kachin Burmese amber.
Phylogenetic justification: Based on the presence of bilateral strobili, Schmidt et al [74] were able to confidently assign six fossil species to the subgenus *Stachygynandrum.*
Minimum age: 98.17 Ma
Maximum age: 451 Ma
Age justification: The age of Burmese amber has long been contested, with estimates ranging between the Miocene and Cretaceous. Claims of an upper Albian age based on ammonite biostratigraphy [44] have not been verified [45]. However, a U-Pb age of 98.79 Ma ± 0.62 Myr was obtained from zircons in volcanoclastics deposited contemporaenously with the amber [46]. Shi et al. (2012) and Yu et al. (2019) interpret this as a minimum age for the amber since it is bioeroded. Nevertheless, in the absence of other evidence, this U-Pb zircon age can be used to established a minimum constraint of 98.17 Ma [44].

C_29: Lycopodiophyta: Lycopodioideae – Lycopodielloideae

Fossil Taxon and Specimen: *Retitriletes austroclavatidites* [GIX-SHA08-5-$2; R32/1, orschungsstelle für Paläobotanik at the Institut für Geologie und Paläontologie, Westfälische Wilhelms-Universität Münster, Germany], from the Section Peak Formation
Phylogenetic Justification: *Retitriletes* is a spore genus which bears strong resemblance with the spores of extant *Lycopodium* on the basis of their isotetrahedral structure and distinctive reticulate ornamentation on the distal surface [75-77]. They are the first spores which can reliably be assigned to the Lycopodoioideae, and thus we follow Bauret *et al.* [76] in providing a minimum age for the divergence of the Lycopodioideae and Lycopodielloideae.
Minimum age: 199.2 Ma
Maximum age: 451 Ma

Age justification: The earliest record of *Retitriletes* is recorded by Bomfleur *et al.* [78] in the upper part of the Section Peak at Priestly Glacier cliff. The assemblage is assigned to Price's APJ1 assemblage based on the presence of intrastriate *Classopollis ssp.* and the absence of *Ichyosporites crateris-punctatus [79]*, advocating and early-middle Sinemurian age. Radiometric ages of detrital zircons from c. 10m below the sample indicated ages of 214 ± 3 Ma and from c. 15m above the sample of 198 ± 2 Ma [80] which is congruent with a Sinemurian assignment. Thus, a minimum age constraint of 199.5 ± 0.3 Ma was proposed [81].

C_30: Euphyllophyta

Fossil taxon and specimen: *Kenrickia bivena* [Axis in slab 557820-5, Smithsonian Institution, NMNH] from the Battery Point Formation (Early Devonian), Quebec, Canada
Phylogenetic justification: A cladistic analysis by Toledo et al. [82] of 50 morphological characters supported *Kenrickia* as sister to all other radiatopsids, a lineage within euphyllophytes and outgroup to remaining progymnosperms and seed plants.
Minimum age: 393.2 Ma
Maximum age: 451 Ma
Age justification*:* Material was collected from the south shore of Gaspé Bay, near Douglastown, QC, Canada. The fossils form part of an allochthonous assemblage preserved in fluvial to coastal deposits [83, 84]. Palynological assemblages indicate the deposits near Douglastown belong to the late Emsian [85] and so our minimum age is based on the boundary of the Emsian, 394.3 ± 1.1 Ma [86].

C_31: Monilophyta: *Equisetum* - Pteridophyta

Fossil taxon and specimen: *Ibyka amphikoma*
Phylogenetic justification: Following Morris *et al.* [5]
Minimum age: 384.7 Ma
Maximum age: 451 Ma
Age justification*:* Following Morris *et al.* [5]

C_32: Monilophyta: *Equisetum subg. Equisetum – Equisetum subg. Hippochaete*

Fossil taxon and specimen: *Equisetum fluviatoides* [US1-103] from 34 metres above the base of the Ravenscrag Formation at Ravenscrag Butte, 17 Km southwest of Eastend, Saskatchewan, Canada [87].
Phylogenetic justification: *Equisetum fluviatoides* is identified as belonging to the crown group *Equisetum* based on vegetative and reproductive similarities to the extant species *E. fluviatile* [87]. A phylogenetic analysis by Elgorriaga et al. [88] placed *E. fluviatoides* as sister to *E. fluviatile* or *E. diffusum* within the subgenus *Equisetum*. The present study analysed the same phenotype and molecular data in a Bayesian framework and was not able to resolve the position of *E. fluviatoides* within the subgenus *Equisetum* but in a more basal position as sister to clade containing *E. fluviatile, E. diffusum* and *E. arvense*. Following our results, *E. fluviatoides* was used to calibrate the divergence of subgenus *Equisetum* from subgenus *Hippochaete*, here representing the crown group of *Equisetum*.
Minimum age: 64.86 Ma
Maximum age: 451.0 Ma
Age justification**:** The minimum age constraint is based on the holotype of *Equisetum fluviatoides,* described from 34 metres above the base of the Ravenscrag Formation at Ravenscrag Butte, Saskatchewan, Canada by McIver and Basinger [87] who estimate an

early Palaeocene age on the basis that the Ravenscrag Formation conformably overlies the Ferris/No.1 Coal Zone which at this site is established to approximate the Cretaceous-Palaeocene Boundary based on palynostratigraphy, about 3 metres below the top of magnetostratigraphic zone 29r [89]. Thus, the horizon from which the holotype of *Equisetum fluviatoides* was derived falls within the 29n magnetozone, the minimum age of which can be constrained by the 29n-28r boundary, which is dated to 64.86 Ma [22].

C_33: Monilophyta: Marratiopsida – Psilotopsida

Fossil taxon and specimen: *Psaronius simplicaulis*
Phylogenetic justification: Following Morris *et al.* [5]
Minimum age: 318.71 Ma
Maximum age: 451.0 Ma
Age justification*:* Following Morris *et al.* [5]

C_34: Monilophyta: *Ophioglossum – Sceptridium*

Fossil taxon and specimen: *Botrychium wightonii* [S1333 in the Palaeobotanical Herbarium, Department of Botany, University of Alberta] from the Palaeocene Genesee locality, North Saskatchewan River, Canada.
Phylogenetic justification: Based on the morphology of sporophores and trophophores, Rothwell & Stockey [90] concluded that *B. wightonii* could be assigned to the extant genus *Botrychium* with high confidence.
Minimum age*:* 65.5
Maximum age: 451.0 Ma
Age justification: Material was collected from the Genesee locality, along the bank of the North Saskatchewan River 45 miles southwest of Edmonton, Canada. The megafloral assemblage at the Genesee locality indicates a Tiffanian age [91]. More recently, Stockey *et al.* [92] indicated that the Genesee flora belonged to the upper Scollard Formation, pollen zone P1, which correlates with the *Wodehouseia fimbriata* biozone [93], indicating an earliest Palaeocene age. U-Pb dating of the Scollard Formation and the overlying Battle Formation indicates an lower bound of 65.5 Ma for the *Wodehouseia fimbriata* biozone [94].

C_35: Monilophyta: Gleicheniales – [Cyatheales – Polypodiales]

Fossil taxon and specimen*:* *Oligocarpia gothanii* [S138027–S138989, S147989 and KC 1983–1, Department of Palaeobotany of the Swedish Natural History Museum, Stockholm] from the Mid Permian of the Shihhotse valley near Ch'en-chia-yu near Taiyuan City in Shanxi Province, China [95].
Phylogenetic justification: *Oligocarpia* has been associated with gleicheniaceous ferns on the basis of both spore and leaf morphology [95, 96].
Minimum age: 268.3 Ma
Maximum age: 451.0 Ma
Age justification: *Oligocarpia gothanii* was collected from the beds of the south side of the Shihhotse valley near Ch'en-chia-yu near Taiyuan City in Shanxi Province, China [95]. These beds belong to the Lower Shihhotse Formation, which has been correlated with the Kungarian-Roadian stages of the Mid Permian [97, 98]. Thus, our minimum age is based on the upper boundary of the Roadian stage, estimated at 268.8 ± 0.5 Ma.

C_36: Monilophyta: Cyatheaceae – Thyrsopteridaceae

Fossil Taxon and Specimen: *Oguracaulis banksii* [Z2286, Tasmania Museum and Art Gallery, Hobart, Tasmania] from the Lune River Fossil Site (Early to Middle Jurassic), Ida Bay, Tasmania [99].
Phylogenetic Justification: Phylogenetic analysis based on characters derived from fossilized stems placed *Oguracaulis banksii* as sister to *Thyrsopteris* [100].
Minimum age: 178 Ma
Maximum age: 451 Ma
Age justification: The flora of the Lune River area, Tasmania was initially estimated as Tertiary based on the similarity of the assemblage to others across Tasmania [101]. However, a revision of the taxonomic affinities of the flora suggested a Late Jurassic to Early Cretaceous age [102]. A basalt outcrop upslope of the fossil localities was shown to be comagmatic with Jurassic dolerite [103] and volcanolithic sandstone containing the fossils was recently dated at 182 ± 4 Ma (U-Pb zircon) [104], indicating a Pliensbachian-Toarcian age. Thus, we propose a minimum age constraint of 178 Ma.

C_37: Monilophyta: Lindseaceae – Lonchitidaceae

Fossil Taxon and Specimen: Lindsaceae gen. sp. indet. [GZG.BST.21952 in the Geoscientific Collections of the Georg August University Göttingen, Germany] from the Burmese Amber, Myanmar.
Phylogenetic Justification: The fusion of multiple veins below elongate terminal sori allows unambiguous assignment to Lindsaceae [105].
Minimum age: 98.17
Maximum age: 451
Age Justification: The age of Burmese amber has long been contested, with estimates ranging between the Miocene and Cretaceous. Claims of an upper Albian age based on ammonite biostratigraphy [44] have not been verified [45]. However, a U-Pb age of 98.79 Ma ± 0.62 Myr was obtained from zircons in volcanoclastics deposited contemporaenously with the amber [46]. Shi et al. (2012) and Yu et al. (2019) interpret this as a minimum age for the amber since it is bioeroded. Nevertheless, in the absence of other evidence, this U-Pb zircon age can be used to established a minimum constraint of 98.17 Ma [44].

C_38: Monilophyta: Pteridiinae + Polypodiinae

Fossil Taxon and Specimen: *Pteris sp.* [Palaeontological Institute, Moscow, Catalogue no. 4799-10] from the mid-Cenomanian of the Nammoura locality, Mount-Liban, Lebanon [106].
Phylogenetic Justification: The specimen was assigned to the extant genus *Pteris* based on strong similarities in pinnule shape and coenosori position [106]. Schneider *et al.* supported its position as a pteridoid fern, but thought that no further assignment was possible and it was used as a minimum age for the divergence of Pteridaceae and Polypodiinae [107].
Minimum age: 100.3 Ma
Maximum age: 451 Ma
Age justification: The Nammoura lagerstätte is estimated as latest middle Cenomanian in age based on the foraminiferan biostratigraphy and the characteristic species *Rhapydionina laurinensis* [106]. The upper units of the bed are marked by the presence of orbitolinids, indicating a middle Cenomanian age, which are absent in the lower units. As the unit from which the specimen was collected is not apparent, we took the upper boundary of the Cenomanian as the minimum age constraint, estimated at 100.5 ± 0.1, thus 100.3 Ma

C_39: Monilophta: Eupolypods I + Eupolypods II

Fossil Taxon and Specimen: *Woodwardia sp.* from the Jose Creek Member, McRae Formation, New Mexico, USA [108].
Phylogenetic Justification: Leaf impressions demonstrating diagnostic traits including a toothed margin, sickle-shaped pinnules, veins that anastomose at the midvein to form costal areoles, veins outside the costal areoles that run to the margin, and the absence of freely ending veinlets indicate a membership to the extant genus *Woodwardia*, a member of the Eupolypods II clade (Aspleniineae) [108].
Minimum age: 72 Ma
Maximum age: 451
Age justification: Leaf impression were collected from the lower Jose Creek Member of the McRae Formation. U-Pb age estimates from multiple horizons within the McRae formation have confirmed a Late Campanian age, rather than the previously suggested Maastrichtian [109]. We use the upper boundary of the Campanian, 72.2 ± 0.2 Ma, thus 72 Ma, as a minimum age constraint.
Discussion: *Holttumopteris burmensis*, a putative crown member of Eupolypods II was recently describe from Early Cretaceous amber deposits in Myanmar [110]. The authors decided that it was likely a member of the derived family Thelypteridaceae, but based on the lack of preservation of key characters could not confidently assign it to an extant family.

C_40: Spermatophyta: Acrogymnospermae – Angiospermae

Fossil taxon and specimen: *Cordaixylon iowensis*
Phylogenetic justification: Following Morris *et al.* [5]
Minimum age: 308.14 Ma
Maximum age: 365.63 Ma
Age justification: Following Morris *et al.* [5]

C_41: Acrogymnospermae: [Ginkgoopsida + Cycadopsida] – Pinopsida

Fossil taxon and specimen: *Cordaixylon iowensis*
Phylogenetic justification: Following Morris *et al.* [5]
Minimum age: 308.14 Ma
Maximum age: 365.63 Ma
Age justification: Following Morris *et al.* [5]

C_42: Acrogymnospermae: Ginkgoopsida – Cycadopsida

Fossil taxon and specimen: *Iratinia australis* [MCT1487-PB, Museu de Ciências da Terra, Rio de Janeiro, Brazil] from the Irati Formation of the Kungurian of the Paraná Basin, Brazil.
Phylogenetic justification: Though initially proposed as a lycopsid [111], based on multiple morpho-anatomical traits Spiekermann *et al.* [112] determined that *I. australis* belonged to the extant order Cycadales.
Minimum age: 274 Ma
Maximum age: 365.63 Ma
Age justification: Material was collected from the Irati Formation, the lowermost unit of the Passa Dois Group [ref]. The Assistência Member from the upper part of the Irati Formation was U-Pb zircon dated to 279.9 ± 4.8 to 280 ± 3 Ma [113], placing it within the Kungurian.

The Taquaral Member at the base of the formation was also placed in the Kungurian based on palynological evidence [114]. Thus, a Kungurian age is determined for the Irati Formation and a minimum age of 274.4 ± 0.5 Ma.

C_43: Pinopsida: Taxaceae - Cupressaceae

Fossil taxon and specimen: *Palaeotaxus rediviva* from the Rhaetian of southern Sweden.
Phylogenetic justification: *Palaeotaxus* forms the first credible record of the family Taxaceae and was placed within Taxaceae in a phylogenetic analysis of morphological characters [115, 116].
Minimum age: 201.2 Ma
Maximum age: 321.48 Ma
Age justification*:* Material was collected from the upper coal bed of the Skromberga Colliery in Scania, Sweden. The Skromberga locality has been assigned to the Bjuv member [117] and Pott & McLoughlin and McElwain et al indicate a Rhaetian age based on the correlation of the *Thaumatopteris-Lepidopteris* flora [118-120]. Thus, a minimum age is established based on the upper boundary of the Rhaetian 201.4 ± 0.2 Ma.

C_44: Pinopsida: *Cunninghamia – Juniperus*

Fossil taxon and specimen: *Chamaeocyparis corpulenta* from the Upper Cretaceous Comox Formation of Vancouver Island, British Columbia.
Phylogenetic justification: Based on morphological similarities of the seed cone structure and the cones on the axes, *C. corpulenta* is considered the oldest known representative of the Cupresseae [121, 122].
Minimum age: 83.2
Maximum age: 321.48 Ma
Age justification: Material was recovered from the carbonaceous shales from the No.2 coal seam within the Cumberland Member of the Comox Formation, Vancouver Island [123]. Marine fauna above and below the Comox formation indicates a Santonian age and thus a minimum of 83.7 ± 0.5 Ma.

C_45: Pinopsida: Pinales – Gnetales

Fossil taxon and specimen: *Eathiestrobus mackenziei*
[Specimen Pb483, Hunterian Museum, Glasgow, Scotland] from Eathie, Black Isle, Scotland.
Phylogenetic justification: Based on a combination of diagnostic seed, cone and scale morphologies, *Eathiestrobus* was considered a member of Pinaceae [124]. However, the wide bract is a feature that distinguishes *Eathiestrobus* from all extant genera.
Minimum age: 153.62 Ma
Maximum age: 321.48 Ma
Age justification: Coastal Jurassic exposures at Eathie are from the Kimmeridge Clay Formation [125]. Ammonite stratigraphy indicates belonging to the *Cymodoce* chronozone [126], yet dinoflagellate stratigraphy supports a placement in the *Baylei* zone (Lower Kimmerigian). The top of the *Cymodoce* zone correlates with the magnetostratigraphic zone M24, the top of which is dated to 153.62 Ma [127].

C_46: Pinopsida: *Pinus – Cedrus*

Fossil taxon and specimen: *Pinus yorkshirensis* [BU4737 at the Lapworth Museum, University of Birmingham, United Kingdom] from the early Cretaceous Wealden of Yorkshire, United Kingdom.
Phylogenetic justification: A cladistic analysis of morphological characters resolved *P. yorkshirensis* as a crown-group member of *Pinus* [128].
Minimum age: 129 Ma
Maximum age: 321.48 Ma
Age justification: Material comes from the Speeton Clay Formation, north of Speeton, Yorkshire. Palynological samples collected from the same material include the dinoflagellate species *Callaiosphaeridium asymmetricum, Odontochitina operculata* and *?Cribroperidinium confossum* [128] which constrains the sediment to Late Hauterivian to Early Barremian [129]. Based on the range bases of these taxa, Ryberg *et al.* conclude that the sediment was formed during the Hauterivian-Barremian transition (131-129 Ma) and thus, we propose a minimum age of 129 Ma.

C_47: Pinopsida: *Pinus* subg. *Pinus – Pinus* subg. *Strobus*

Fossil taxon and specimen: *Pinus triphylla* [E.C Jeffrey Collection, Harvard University, USA] from the Late Cretaceous Raritan Formation, Staten Island, USA.
Phylogenetic justification: *P. triphylla* has been considered a stem lineage member of the subgenus *Pinus* based on the synapomorphy of possessing two vascular bundles in the needles [130, 131].
Minimum age: 89.37 Ma
Maximum age: 321.48 Ma
Age justification: Material was collected from the Androvette Clay Pit, Staten Island, part of the Raritan Formation. Based on palynological evidence, a Turonian age is proposed for the formation [55, 132, 133]. Thus, we propose a minimum age based on the lower boundary of the Turonian of 89.75 ± 0.38.

C_48: Pinopsida: *Pinus* subsection *Australes*

Fossil taxon and specimen: *Pinus storeyana* [University of California, Museum of Paleontology, holotype 7234] from the Coal Valley Formation, Celetom Quarry, Nevada, USA
Phylogenetic justification: Based on seed cone morphology, Saladin *et al.* placed *P. storeyana* within the *Attenuatae* subclade [130, 134].
Minimum age: 9 Ma
Maximum age: 321.48 Ma
Age justification: K-Ar dating of the Coal Valley Formation, Wassuk Group, Nevada suggests it was deposited between 13 and 9 Ma [135]. Thus, a minimum age of 9 Ma is proposed

C_49: Angiospermae

Fossil taxon and specimen: Tricolpate pollen grain
Phylogenetic justification: Following Morris *et al.* [5]
Minimum age: 125 Ma
Maximum age: 247Ma
Age justification*:* Following Morris *et al.* [5]

C_50: Angiospermae: Nympheales

Fossil taxon and specimen: Tricolpate pollen grain
Phylogenetic justification: Following Morris *et al.* [5]
Minimum age: 125 Ma
Maximum age: 247Ma
Age justification: Following Morris *et al.* [5]

C_51: Angiospermae: Austrobaileyales

Fossil taxon and specimen: Tricolpate pollen grain
Phylogenetic justification: Following Morris *et al.* [5]
Minimum age: 125 Ma
Maximum age: 247Ma
Age justification: Following Morris *et al.* [5]

C_52: Mesangiospermae

Fossil taxon and specimen: Tricolpate pollen grain
Phylogenetic justification: Following Morris *et al.* [5]
Minimum age: 125 Ma
Maximum age: 247Ma
Age justification: Following Morris *et al.* [5]

C_53: Magnoliids: [Magnoliales + Laurales] + Piperales

Fossil taxon and specimen: *Endressinia brasiliana*
Phylogenetic justification: Following Morris *et al.* [5]
Minimum age: 110.87 Ma
Maximum age: 247Ma
Age justification: Following Morris *et al.* [5]

C_54: Piperales: *Houttuynia – Saruma*

Fossil taxon and specimen: *Saururus tuckerae*
Phylogenetic justification: Following Morris *et al.* [5]
Minimum age: 44.3 Ma
Maximum age: 247Ma
Age justification: Following Morris *et al.* [5]

C_55: Eudicotyledonae

Fossil taxon and specimen: *Hyrcantha decussata*
Phylogenetic justification: Following Morris *et al.* [5]
Minimum age: 119.6 Ma
Maximum age: 128.63 Ma
Age justification: Following Morris *et al.* [5]

C_56: Eudicotyledonae: Vitales

Fossil taxon and specimen: *Paleoclusia chevalieri*
Phylogenetic justification: Following Barba-Montoya *et al.* [136]

Minimum age: 85.84 Ma
Maximum age: 128.63 Ma
Age justification*:* Following Barba-Montoya *et al.* [136]

C_57: Eudicotyledonae: Rosids minus Vitales

Fossil taxon and specimen: *Paleoclusia chevalieri*
Phylogenetic justification: Following Barba-Montoya *et al.* [136]
Minimum age: 85.8 Ma
Maximum age: 128.63 Ma
Age justification*:* Following Barba-Montoya *et al.* [136]

C_58: Eudicotyledonae: Ericales

Fossil taxon and specimen: *Paleoenkianthus sayrevillensis*
Phylogenetic justification: Following Barba-Montoya *et al.* [136]
Minimum age: 85.8 Ma
Maximum age: 128.63 Ma
Age justification*:* Following Barba-Montoya *et al.* [136]

C_59: Eudicotyledonae: Myrtales

Fossil taxon and specimen: *Esqueiria futabensis*
Phylogenetic justification: Following Barba-Montoya *et al.* [136]
Minimum age: 83.3 Ma
Maximum age: 128.63 Ma
Age justification*:* Following Barba-Montoya *et al.* [136]

C_60: Eudicotyledonae: Asteraceae

Fossil taxon and specimen: *Tubulifloridites antipodica*
Phylogenetic justification: Following Barba-Montoya *et al.* [136]
Minimum age: 41.5 Ma
Maximum age: 128.63 Ma
Age justification*:* Following Barba-Montoya *et al.* [136]

C_61: Eudicotyledonae: Salicaceae

Fossil taxon and specimen: *Pseudosalix handleyi*
Phylogenetic justification: Following Barba-Montoya *et al.* [136]
Minimum age: 48.57 Ma
Maximum age: 128.63 Ma
Age justification*:* Following Barba-Montoya *et al.* [136]

C_62: Eudicotyledonae: Solanales

Fossil taxon and specimen: *Solanites crassus*
Phylogenetic justification: Following Barba-Montoya *et al.* [136]
Minimum age: 37.3 Ma
Maximum age: 128.63 Ma

Age justification: Following Barba-Montoya *et al.* [136]

C_63: Monocotyledonae

Fossil taxon and specimen: *Liliacidites sp.*
Phylogenetic justification: Following Barba-Montoya *et al.* [136]
Minimum age: 113 Ma
Maximum age: 128.63 Ma
Age justification: Following Barba-Montoya *et al.* [136]

C_64: Monocotyledonae: Dioscoreales + Core Monocots

Fossil taxon and specimen: *Cratolirion bognerianum* [MB.Pb. 1997/1233 (repository: Museum für Naturkunde, Berlin)] from the Crato Formation (Early Cretaceous), Santana do Cariri, Brazil [137].
Phylogenetic justification: Phylogenetic analyses indicated a membership within the Pandanales + Dioscoreales lineage (Petrosaviidae) [137].
Minimum age: 119.5 Ma
Maximum age: 128.63 Ma
Age justification: The age of the [5, 138]Crato Formation has been much debated but a 123 Ma ± 3.5 Myro been obtained for the overlying Ipubi Formation using Re-Os isotope dating [139], providing for a minimum constraint of 119.5 Ma [138].

C_65: Monocotyledonae: Riponogaceae (*Colcichum – Smilax*) ** NEW **

Fossil taxon and specimen: *Ripogonum tasmanicum* from the Macquarie Harbour Formation (Early Eocene) from the central west coast of Tasmania [140]
Phylogenetic justification: Based on a phylogenetic analysis of morphological characters, *Ripogonum tasmanicum* was placed within the extant genus *Ripogonum* (Ripogonaceae, Liliales). Phylogenetic analyses identify Riponogaceae and Smilaceae being sister lineages respective to Colchicaceae. ***Minimum age:*** 50.5 Ma
Maximum age: 128.63 Ma
Age justification: Fossil leaves were collected from mudstone deposits at the Lowana Road site, the Macquarie Harbour Formation, Tasmania. Spores, pollen and dinoflagellates from the site were attributed to the Planktonic Foraminiferal Zone P8-9 [141]corresponding to a Mid-Ypresian age. The minimum age of the deposit is determined based on correlation of the Upper *Malvacipollis diversus* spore-pollen zone with zones P7 to P8 of Partridge [142] (52.5-50.5 Ma). Thus, a minimum age of 50.5 Ma is provided.

C_66: Monocotyledonae: Arecales – Poales
Fossil taxon and specimen: *Sabalites carolinensis*
Phylogenetic justification: Following Barba-Montoya *et al.* [136]
Minimum age: 83.41 Ma
Maximum age: 128.63 Ma
Age justification: Following Barba-Montoya *et al.* [136]

C_67: Poales: BOP-PACMAD

Fossil taxon and specimen: *Changii indicum* [Slide 13160, Q-14-3, Birbal Sahni I. Palaeobotany, Lucknow, India] recovered from Coprolites from red clays in Lameta

Formation, associated with the Deccan Volcanics (infra- and inter-trappan) at Pisdura (East and South sections), Central India. [143].
Phylogenetic justification: Based on phylogenetic analyses, Prasad *et al.* [143] determined *C. indicum* as a member of Erhartoideae (Poaceae). More recent analyses have questioned this placement, and have suggested that it could belong to a number of tribes. As such, we follow Christin *et al.* [144] in a more conservative placement as a stem group member of the BOP clade.
Minimum age: 66.261 Ma
Maximum age: 128.63 Ma
Age justification: Prasad *et al.* [61] interpreted the minimum age of *Changii indicum* as 65.0 Ma, while Iles *et al.* [145] adopted the radiometric and magnetostrategraphic dating of the Deccan Traps by Courtillot and Ren [146], supportted by age constraints of associated dinosaur coprolites to infer a minimum constraint of 66.0 Ma, based on the dating of the Maastrichtian-Palaeocene Boundary; this would revise to 65.98 Ma following Ogg & Hinnov [138]. However, the dating of the Deccan Traps has been revised [147] and, given the fossil occurrences predate the volcanic sequences [61] we can establish a minimum constraint based on the age of the oldest phase of volcanism, dated to 66.288 Ma ± 0.027 Myr, thus 66.261 Ma.

C_68: Poales: *Brachypodium – Oryza* ** NEW **

Fossil taxon and specimen: *Stipa florissanti* [Syntypes: 34750, 34751 (USNM)] from Florissant and Florissant Fossil Beds National Monument, CO, USA [148]
Phylogenetic justification: Based on morphology this fossil was assigned the extant grass lineage *Stipa.* Extant *Stipa* has a strictly Old World distribution [149], so the generic assignment has been questioned [150]. We follow Iles *et al.* [145] and their assignment to the stem group Stipeae, itself nested within Pooideae [151].
Minimum age: 33.705 Ma
Maximum age: 128.63 Ma
Age justification: The age interpretation of the Florissant Formation is usually based on Evanoff et al. [152] who used $^{40}Ar/^{39}Ar$ dating of sanidine crystals in detrital pumice, yielding an age of 34.07 Ma ± 0.1 Myr. However, its detrital origin renders this a maximum constraint on the age of the fossil. Nevertheless, Florissant Formation falls fully within the C13r magnetozone [153] and the base of the succeeding C13n magnetozone is dated to 33.705 [22]

References for Calibrations

1. Tang Q., Pang K., Yuan X., Xiao S. 2020 A one-billion-year-old multicellular chlorophyte. *Nature Ecology & Evolution* **4**(4), 543-549. (doi:10.1038/s41559-020-1122-9).
2. Zhang S.-H., Zhao Y., Ye H., Hu G.-H. 2016 Early Neoproterozoic emplacement of the diabase sill swarms in the Liaodong Peninsula and pre-magmatic uplift of the southeastern North China Craton. *Precambrian Research* **272**, 203-225.
3. Yang D.-B., Xu W.-L., Xu Y.-G., Wang Q.-H., Pei F.-P., Wang F. 2012 U–Pb ages and Hf isotope data from detrital zircons in the Neoproterozoic sandstones of northern Jiangsu and southern Liaoning Provinces, China: implications for the Late Precambrian evolution of the southeastern North China Craton. *Precambrian Research* **216**, 162-176.
4. Zhao H., Zhang S., Ding J., Chang L., Ren Q., Li H., Yang T., Wu H. 2019 New geochronologic and paleomagnetic results from early Neoproterozoic mafic sills and late Mesoproterozoic to early Neoproterozoic successions in the eastern North China Craton, and implications for the reconstruction of Rodinia. *GSA Bulletin* **132**(3-4), 739-766. (doi:10.1130/b35198.1).

5. Morris J.L., Puttick M.N., Clark J.W., Edwards D., Kenrick P., Pressel S., Wellman C.H., Yang Z., Schneider H., Donoghue P.C.J. 2018 The timescale of early land plant evolution. *Proceedings of the National Academy of Sciences* **115**(10), E2274-E2283. (doi:10.1073/pnas.1719588115).
6. Loiseau O., Weigand A., Noben S., Rolland J., Silvestro D., Kessler M., Lehnert M., Salamin N. 2020 Slowly but surely: gradual diversification and phenotypic evolution in the hyper-diverse tree fern family Cyatheaceae. *Annals of Botany* **125**(1), 93-103.
7. Samuelsson J., Dawes P.R., Vidal G. 1999 Organic-walled microfossils from the Proterozoic Thule Supergroup, Northwest Greenland. *Precambrian Research* **96**(1), 1-23. (doi:https://doi.org/10.1016/S0301-9268(98)00123-5).
8. Tomescu A., Bomfleur B., Bippus A., Savoretti A. 2018 Why Are Bryophytes So Rare in the Fossil Record? A Spotlight on Taphonomy and Fossil Preservation. (pp. 375-416.
9. Labandeira C.C., Tremblay S.L., Bartowski K.E., VanAller Hernick L. 2014 Middle Devonian liverwort herbivory and antiherbivore defence. *New Phytologist* **202**(1), 247-258. (doi:10.1111/nph.12643).
10. he X., Sun Y., Zhu R.-L. 2013 The Oil Bodies of Liverworts: Unique and Important Organelles in Land Plants. *Critical Reviews in Plant Sciences* **32**. (doi:10.1080/07352689.2013.765765).
11. Hernick L.V., Landing E., Bartowski K.E. 2008 Earth's oldest liverworts—Metzgeriothallus sharonae sp. nov. from the Middle Devonian (Givetian) of eastern New York, USA. *Review of Palaeobotany and Palynology* **148**(2), 154-162. (doi:https://doi.org/10.1016/j.revpalbo.2007.09.002).
12. Flores J.R., Bippus A.C., Suárez G.M., Hyvönen J. 2020 Defying death: incorporating fossils into the phylogeny of the complex thalloid liverworts (Marchantiidae, Marchantiophyta) confirms high order clades but reveals discrepancies in family-level relationships. *Cladistics* **n/a**(n/a). (doi:https://doi.org/10.1111/cla.12442).
13. Carpenter D.K. 2017 Charcoal, forests, and Earth's palaeozoic geochemical oxygen cycle, University of Southampton.
14. Willis B., Bridge J. 1988 Evolution of Catskill river systems, New York state.
15. Richardson J.B. 1986 Silurian and Devonian spore zones of the Old Red Sandstone continent and adjacent regions. *Geol Surv Canada, Bulletin* **354**, pl. 1-21.
16. Becker R., Gradstein F., Hammer O. 2012 The devonian period. In *The geologic time scale* (pp. 559-601, Elsevier.
17. Villarreal J.C., Renner S.S. 2012 Hornwort pyrenoids, carbon-concentrating structures, evolved and were lost at least five times during the last 100 million years. *Proceedings of the National Academy of Sciences* **109**(46), 18873-18878. (doi:10.1073/pnas.1213498109).
18. Archangelsky S., Seoane L. 1996 Palynological studies of the baqueró formation (cretaceous), Santa Cruz Province, Argentina. VII. *Ameghiniana* **33**, 307-313.
19. Passalia M.G., Llorens M., CÈsari S.N., Limarino C.O., Limarino C.O., Loinaze V.S.P., Vera E.I., Vera E.I. 2016 Revised stratigraphic framework of the Cretaceous in the Bajo Grande area (Argentinean Patagonia) inferred from new u Pb ages and palynology. *Cretaceous Research* **60**, 152-166.
20. Chitaley S.D., Yawale N.R. 1980 On Notothylites nirulai gen. et sp. nov., a petrified Sporogonium from the Deccan Intertrappean beds of Mohgaonkalan, Madhya Pradesh. (
21. Thakre D., Samant B., Mohabey D., Sangode S.J., Srivastava P., Kapgate D., Mahajan R., Upreti N., Manchester S. 2017 A new insight into age and environments of intertrappean beds of Mohgaon Kalan, Chhindwara District, Madhya Pradesh using palynology, megaflora, magnetostratigraphy and clay mineralogy. *Current science* **VOL. 112**, 2193-2197.

22. Vandenberghe N., Hilgen F.J., Speijer R.P. 2012 The Paleogene Period. In *The geologic timescale 2012* (eds. Gradstein F.M., Ogg J.G., Schmitz M., Ogg G.), pp. 855-921. Ansterdam, Elsevier.
23. Graham A. 1987 Miocene communities and paleoenvironments of southern Costa Rica. *American Journal of Botany* **74**(10), 1501-1518.
24. Cassell D.T., Sen Gupta B.K. 1989 Foraminiferal stratigraphy and paleonvironments of the Tertiary Uscari Formation, Limon Basin, Costa Rica. *Journal of Foraminiferal Research* **19**(1), 52-71. (doi:10.2113/gsjfr.19.1.52).
25. Hilgen F., Lourens L., Dam J.A. 2012 The Neogene Period. *The Geologic Time Scale 2012* **2**, 923-978.
26. Seward A. 1904 The Jurassic Flora, 2: Liassic and Oolitic Floras of England. Catalogue of the Mesozoic Plants in the Department of Geology. *Br Mus Nat Hist London* **4**(16), 192.
27. Li R., Li X., Deng S., Sun B. 2020 Morphology and microstructure of Pellites hamiensis nov. sp., a Middle Jurassic liverwort from northwestern China and its evolutionary significance. *Geobios*. (doi:https://doi.org/10.1016/j.geobios.2020.07.003).
28. Hongfu L. Early-Middle Jurassic Palynological Assemblages of Tmlufan-Hami Basin.
29. Deng S., Lu Y., Fan R., Pan Y., Cheng X., Fu G., Wang Q., Pan H., Shen Y., Wang Y. 2010 The Jurassic System of Northern Xinjiang, China. In *Contributions to the 8th International Congress on the Jurassic System University of Science and Technology of China Press, Hefei* (
30. Ogg J.G., Ogg G.M., Gradstein F.M. 2016 *A concise geologic time scale: 2016*, Elsevier.
31. Moisan P., Voigt S., Schneider J.W., Kerp H. 2012 New fossil bryophytes from the Triassic Madygen Lagerstätte (SW Kyrgyzstan). *Review of Palaeobotany and Palynology* **187**, 29-37.
32. Schuster R.M. 1966 Hepaticae and Anthocerotae of North America east of the hundredth meridian.
33. Dobruskina I.A. 1995 *Keuper (Triassic) Flora from Middle Asia (Madygen, Southern Fergana): Bulletin 5*, New Mexico Museum of Natural History and Science.
34. Shcherbakov D. 2008 Madygen, Triassic Lagerstätte number one, before and after Sharov. *Alavesia* **2**, 113-124.
35. Krassilov V.A. 1973 Mesozoic bryophytes from the Bureja Basin, Far East of the USSR. *Palaeontographica Abteilung B*, 95-105.
36. Krassilov V. 1970 Leafy hepatics from the Jurassic of the Bureja basin. *Paleontol Zurn* **3**, 131-142.
37. Heinrichs J., Hentschel J., Wilson R., Feldberg K., Schneider H. 2007 Evolution of Leafy Liverworts (Jungermanniidae, Marchantiophyta): Estimating Divergence Times from Chloroplast DNA Sequences Using Penalized Likelihood with Integrated Fossil Evidence. *Taxon* **56**(1), 31-44. (doi:10.2307/25065733).
38. Markevich V.S., Bugdaeva E.V. 2009 Palynological evidence for dating Jurassic-Cretaceous boundary sediments in the Bureya basin, Russian Far East. *Russian Journal of Pacific Geology* **3**(3), 284-293. (doi:10.1134/S1819714009030087).
39. Markevich V., Bugdaeva E. 2014 Late jurassic-early cretaceous coal-forming plants of the Bureya Basin, Russian Far East. *Stratigraphy and Geological Correlation* **22**(3), 239-255.
40. Schuster R.M., Janssens J.A. 1989 On Diettertia, an isolated Mesozoic member of the Jungermanniales. *Review of palaeobotany and palynology* **57**(3-4), 277-287.
41. McGookey D., Haun J., Hale L., Goodell H., McCubbin D., Weimer R., Wulf G. 1972 Cretaceous system. *Geologic Atlas of the Rocky Mountain Region: Rocky Mountain Association of Geologists*, 190-228.

42. Decelles P.G. 1986 Sedimentation in a tectonically partitioned, nonmarine foreland basin: The Lower Cretaceous Kootenai Formation, southwestern Montana. *Geological Society of America Bulletin* **97**(8), 911-931.
43. Heinrichs J., Schäfer-Verwimp A., Feldberg K., Schmidt A.R. 2014 The extant liverwort Gackstroemia (Lepidolaenaceae, Porellales) in Cretaceous amber from Myanmar. *Review of Palaeobotany and Palynology* **203**, 48-52. (doi:https://doi.org/10.1016/j.revpalbo.2014.01.004).
44. Cruickshank R., Ko K. 2003 Geology of an amber locality in the Hukawng Valley, northern Myanmar. *Journal of asian earth Sciences* **21**(5), 441-455.
45. Yu T., Kelly R., Mu L., Ross A., Kennedy J., Broly P., Xia F., Zhang H., Wang B., Dilcher D. 2019 An ammonite trapped in Burmese amber. *Proceedings of the National Academy of Sciences* **116**(23), 11345-11350.
46. Shi G., Grimaldi D.A., Harlow G.E., Wang J., Wang J., Yang M., Lei W., Li Q., Li X. 2012 Age constraint on Burmese amber based on U–Pb dating of zircons. *Cretaceous research* **37**, 155-163.
47. Bechteler J., Schmidt A.R., Renner M.A.M., Wang B., Pérez-Escobar O.A., Schäfer-Verwimp A., Feldberg K., Heinrichs J. 2017 A Burmese amber fossil of Radula (Porellales, Jungermanniopsida) provides insights into the Cretaceous evolution of epiphytic lineages of leafy liverworts. *Foss Rec* **20**(2), 201-213. (doi:10.5194/fr-20-201-2017).
48. Heinrichs J., Reiner-Drehwald M.E., Feldberg K., von Konrat M., Hentschel J., Váňa J., Grimaldi D.A., Nascimbene P.C., Schmidt A.R. 2012 The leafy liverwort Frullania (Jungermanniopsida) in the Cretaceous amber forest of Myanmar. *Review of Palaeobotany and Palynology* **169**, 21-28.
49. Hentschel J., Schmidt A.R., Heinrichs J. 2009 Frullania cretacea sp. nov.(Porellales, Jungermanniopsida), a leafy liverwort preserved in Cretaceous amber from Myanmar. *Cryptogamie* **30**(3), 323.
50. Bippus A.C., Stockey R.A., Rothwell G.W., Tomescu A.M. 2017 Extending the fossil record of Polytrichaceae: Early Cretaceous Meantoinea alophosioides gen. et sp. nov., permineralized gametophytes with gemma cups from Vancouver Island. *American journal of botany* **104**(4), 584-597.
51. Bippus A.C., Escapa I.E., Tomescu A.M.F. 2018 Wanted dead or alive (probably dead): Stem group Polytrichaceae. *American Journal of Botany* **105**(8), 1243-1263. (doi:10.1002/ajb2.1096).
52. Sweet A.R. 2000 Applied research on two samples of Cretaceous age
from Vancouver Island, British Columbia as requested by J. Haggart
(GSC Pacifi c, Vancouver). In *Geological Survey of Canada, Natural Resources Canada/Ressources*
Naturelles Canada, Ottawa, Ontario, Canada (
53. Klymiuk A.A., Stockey R.A. 2012 A Lower Cretaceous (Valanginian) seed cone provides the earliest fossil record for Picea (Pinaceae). *American Journal of Botany* **99**(6), 1069-1082. (doi:10.3732/ajb.1100568).
54. Konopka A.S., Herendeen P.S., Merrill G.L.S., Crane P.R. 1997 Sporophytes and gametophytes of Polytrichaceae from the Campanian (Late Cretaceous) of Georgia, USA. *International Journal of Plant Sciences* **158**(4), 489-499.
55. Christopher R.A. 1979 Normapolles and triporate pollen assemblages from the Raritan and Magothy Formations (Upper Cretaceous) of New Jersey. *Palynology* **3**(1), 73-121. (doi:10.1080/01916122.1979.9989185).
56. Konopka A.S., Herendeen P.S., Crane P.R. 1998 Sporophytes and gametophytes of Dicranaceae from the Santonian (Late Cretaceous) of Georgia, USA. *American Journal of Botany* **85**(5), 714-723. (doi:10.2307/2446542).

57. Burnett J.A. 1996 Nannofossils and Upper Cretaceous stage boundaries. *Journal of Nannoplankton Research* **18**, 23-32.
58. Savoretti A., Bippus A.C., Stockey R.A., Rothwell G.W., Tomescu A.M.F. 2018 Grimmiaceae in the Early Cretaceous: Tricarinella crassiphylla gen. et sp. nov. and the value of anatomically preserved bryophytes. *Annals of Botany* **121**(7), 1275-1286. (doi:10.1093/aob/mcy015).
59. Shelton G., Stockey R., Rothwell G.W., Tomescu A. 2016 Krassiloviella Limbelloides GEN. ET SP. NOV.: Additional diversity in the hypnanaean moss family tricostaceae (Valanginian, Vancouver Island, British Columbia). *International Journal of Plant Sciences* **177**, 000-000. (doi:10.1086/688707).
60. Rubinstein C.V., Vajda V. 2019 Baltica cradle of early land plants? Oldest record of trilete spores and diverse cryptospore assemblages; evidence from Ordovician successions of Sweden. *GFF* **141**(3), 181-190. (doi:10.1080/11035897.2019.1636860).
61. Bergström S.M., Lehnert O., Calner M., Joachimski M.M. 2012 A new upper Middle Ordovician–Lower Silurian drillcore standard succession from Borenshult in Östergötland, southern Sweden: 2. Significance of δ13C chemostratigraphy. *Gff* **134**(1), 39-63.
62. Sell B., Ainsaar L., Leslie S. 2013 Precise timing of the Late Ordovician (Sandbian) super-eruptions and associated environmental, biological, and climatological events. *Journal of the Geological Society* **170**(5), 711-714.
63. Bauert H., Isozaki Y., Holmer L.E., Aoki K., Sakata S., Hirata T. 2014 New U–Pb zircon ages of the Sandbian (Upper Ordovician)"Big K-bentonite" in Baltoscandia (Estonia and Sweden) by LA-ICPMS. *GFF* **136**(1), 30-33.
64. Goldman D., Sadler P., Leslie S., Melchin M., Agterberg F., Gradstein F. 2020 The Ordovician Period. In *Geologic Time Scale 2020* (pp. 631-694, Elsevier.
65. Hao S., Xue J., Wang Q., Liu Z. 2007 Yuguangia ordinata gen. et sp. nov., a New Lycopsid from the Middle Devonian (Late Givetian) of Yunnan, China, and Its Phylogenetic Implications. *International Journal of Plant Sciences - INT J PLANT SCI* **168**, 1161-1175. (doi:10.1086/520727).
66. Marshall J.E., Zhu H., Wellman C.H., Berry C.M., Wang Y., Xu H., Breuer P. 2017 Provincial Devonian spores from South China, Saudi Arabia and Australia. *Revue de Micropaléontologie* **60**(3), 403-409.
67. Rowe N. 1988 A herbaceous Lycophyte from the Lower Carboniferous Drybrook Sandstone of the Forest of Dean, Gloucestershire. *Palaeontology* **31**, 69-83.
68. Klaus K., Schulz C., Bauer D., Stützel T. 2016 Historical biogeography of the ancient lycophyte genus Selaginella: Early adaptation to xeric habitats on Pangea. *Cladistics*. (doi:10.1111/cla.12184).
69. Weststrand S., Korall P. 2016 Phylogeny of Selaginellaceae: There is value in morphology after all! *Am J Bot* **103**(12), 2136-2159. (doi:10.3732/ajb.1600156).
70. Lele K., Walton J. 1962 *Fossil flora of the Drybrook Sandstone in the Forest of Dean, Gloucestershire*, British Museum (Natural History).
71. Rowe N. 1988 New observations on the Lower Carboniferous pteridosperm Diplopteridium Walton and an associated synangiate organ. *Botanical journal of the Linnean Society* **97**(2), 125-158.
72. Clayton G., Coquel R., Doubinger J., Gueinn K., Loboziak S., Owens B., Streel M. 1977 Carboniferous miospores of western Europe: illustration and zonation. *Mededelingen-Rijks Geologische Dienst* **29**, 1-71.
73. Poty E., Aretz M., Hance L. 2014 Belgian substages as a basis for an international chronostratigraphic division of the Tournaisian and Viséan. *Geological Magazine* **151**(2), 229-243.

74. Schmidt A.R., Regalado L., Weststrand S., Korall P., Sadowski E.-M., Schneider H., Jansen E., Bechteler J., Krings M., Müller P., et al. 2020 Selaginella was hyperdiverse already in the Cretaceous. *New Phytologist* **228**(4), 1176-1182. (doi:https://doi.org/10.1111/nph.16600).
75. Field A.R., Testo W., Bostock P.D., Holtum J.A., Waycott M. 2016 Molecular phylogenetics and the morphology of the Lycopodiaceae subfamily Huperzioideae supports three genera: Huperzia, Phlegmariurus and Phylloglossum. *Molecular Phylogenetics and Evolution* **94**, 635-657.
76. Bauret L., Field A.R., Gaudeul M., Selosse M.-A., Rouhan G. 2018 First insights on the biogeographical history of Phlegmariurus (Lycopodiaceae), with a focus on Madagascar. *Molecular Phylogenetics and Evolution* **127**, 488-501. (doi:https://doi.org/10.1016/j.ympev.2018.05.004).
77. Juhász M. 1975 Lycopodiaceae spores from Lower Cretaceous deposits of Hungary. *Acta Biological Szeged* **21**, 21-34.
78. Bomfleur B., Schöner R., Schneider J.W., Viereck L., Kerp H., McKellar J.L. 2014 From the Transantarctic Basin to the Ferrar Large Igneous Province—new palynostratigraphic age constraints for Triassic–Jurassic sedimentation and magmatism in East Antarctica. *Review of Palaeobotany and Palynology* **207**, 18-37.
79. Price P. 1997 Permian to Jurassic palynostratigraphic nomenclature of the Bowen and Surat Basins. *The Surat and Bowen basins, south-east Queensland*, 137-178.
80. Elsner M., Schöner R., Gerdes A., Gaupp R. 2013 Reconstruction of the early Mesozoic plate margin of Gondwana by U–Pb ages of detrital zircons from northern Victoria Land, Antarctica. *Geological Society, London, Special Publications* **383**(1), 211-232.
81. Hesselbo S., Ogg J., Ruhl M., Hinnov L., Huang C. 2020 The Jurassic Period. In *Geologic time scale 2020* (pp. 955-1021, Elsevier.
82. Toledo S., Bippus A.C., Atkinson B.A., Bronson A.W., Tomescu A.M.F. 2021 Taxon sampling and alternative hypotheses of relationships in the euphyllophyte plexus that gave rise to seed plants: insights from an Early Devonian radiatopsid. *New Phytologist* **232**(2), 914-927. (doi:https://doi.org/10.1111/nph.17511).
83. Cant D.J., Walker R.G. 1976 Development of a braided-fluvial facies model for the Devonian Battery Point Sandstone, Quebec. *Canadian Journal of Earth Sciences* **13**(1), 102-119.
84. Griffing D.H., Bridge J.S., Hotton C.L. 2000 Coastal-fluvial palaeoenvironments and plant palaeoecology of the Lower Devonian (Emsian), Gaspé Bay, Québec, Canada. *Geological Society, London, Special Publications* **180**(1), 61-84.
85. MCGREGOR D., DC M. 1977 LOWER AND MIDDLE DEVONIAN SPORES OF EASTERN GASPE. II. BIOSTRATIGRAPHY.
86. Becker R., Marshall J., Da Silva A.-C., Agterberg F., Gradstein F., Ogg J. 2020 The devonian period. In *Geologic Time Scale 2020* (pp. 733-810, Elsevier.
87. McIver E.E., Basinger J.F. 1989 The morphology and relationships of Equisetum fluviatoides sp.nov. from the Paleocene Ravenscrag Formation of Saskatchewan, Canada. *Canadian Journal of Botany* **67**(10), 2937-2943. (doi:10.1139/b89-376).
88. Elgorriaga A., Escapa I.H., Rothwell G.W., Tomescu A.M.F., Rubén Cúneo N. 2018 Origin of Equisetum: Evolution of horsetails (Equisetales) within the major euphyllophyte clade Sphenopsida. *American Journal of Botany* **105**(8), 1286-1303. (doi:10.1002/ajb2.1125).
89. Lerbekmo J. 1985 Magnetostratigraphic and biostratigraphic correlations of Maastrichtian to early Paleocene strata between south-central Alberta and southwestern Saskatchewan. *Bulletin of Canadian Petroleum Geology* **33**(2), 213-226.
90. Rothwell G.W., Stockey R. 1989 Fossil Ophioglossales in the Paleocene of Western North America. *American Journal of Botany - AMER J BOT* **76**. (doi:10.2307/2444411).

91. Chandrasekharam A. 1974 Megafossil flora from the Genesee locality, Alberta, Canada.
92. Stockey R.A., Hoffman G.L., Vavrek M.J. 2014 Paleobotany and paleoecology of the Munce's Hill fossil locality near Red Deer, Alberta, Canada. *Paleobotany and Biogeography: A Festschrift for Alan Graham in His 80th Year MSB* **128**, 367-388.
93. Lerbekmo J., Sweet A. 2007 Magnetobiostratigraphy of the continental Paleocene upper Coalspur and Paskapoo formations near Hinton, Alberta. *Bulletin of Canadian Petroleum Geology* **56**(2), 118-146.
94. Eberth D.A., Kamo S.L. 2019 First high-precision U–Pb CA–ID–TIMS age for the Battle Formation (Upper Cretaceous), Red Deer River valley, Alberta, Canada: implications for ages, correlations, and dinosaur biostratigraphy of the Scollard, Frenchman, and Hell Creek formations. *Canadian Journal of Earth Sciences* **56**(10), 1041-1051. (doi:10.1139/cjes-2018-0098).
95. Stevens L., Hilton J. 2009 Ontogeny and ecology of the filicalean fern Oligocarpia gothanii (Gleicheniaceae) from the Middle Permian of China. *American journal of botany* **96**, 475-486. (doi:10.3732/ajb.0800221).
96. Wang Y., Guignard G., Barale G. 1999 Morphological and Ultrastructural Studies on in situ Spores of Oligocarpia (Gleicheniaceae) from the Lower Permian of Xinjiang, China. *Int J Plant Sci* **160**(5), 1035-1045. (doi:10.1086/314178).
97. Glasspool I.J., Hilton J., Collinson M.E., Wang S.-J., Li Cheng S. 2004 Foliar physiognomy in Cathaysian gigantopterids and the potential to track Palaeozoic climates using an extinct plant group. *Palaeogeography, Palaeoclimatology, Palaeoecology* **205**(1), 69-110. (doi:https://doi.org/10.1016/j.palaeo.2003.12.002).
98. Hilton J., Cleal C.J. 2007 The relationship between Euramerican and Cathaysian tropical floras in the Late Palaeozoic: Palaeobiogeographical and palaeogeographical implications. *Earth-Science Reviews* **85**(3), 85-116. (doi:https://doi.org/10.1016/j.earscirev.2007.07.003).
99. Tidwell W.D., Nishida H., Webster N. 1989 Oguracaulis banksii gen. et sp. nov., a mid-Mesozoic tree-fern from Tasmania, Australia. In *Papers and Proceedings of the Royal Society of Tasmania* (pp. 15-25.
100. Lantz T.C., Rothwell G.W., Stockey R.A. 1999 Conantiopteris schuchmanii, gen. et sp. nov., and the Role of Fossils in Resolving the Phylogeny of Cyatheaceae sl. *Journal of Plant Research* **112**(3), 361-381.
101. Gould R.E. 1972 <I>Cibotium tasmanense</I> sp. nov., A fossil tree-fern from the Tertiary of Tasmania. *Australian Journal of Botany* **20**(1), 119-126.
102. Tidwell W.D. 1987 A new species of Osmundacaulis (O. jonesii sp. nov.) from Tasmania, Australia. *Review of Palaeobotany and Palynology* **52**(2-3), 205-216.
103. Hergt J., Chappell B., McCulloch M., McDougall I., Chivas A. 1989 Geochemical and isotopic constraints on the origin of the Jurassic dolerites of Tasmania. *Journal of Petrology* **30**(4), 841-883.
104. Bromfield K., Burrett C., Leslie R.A., Meffre S. 2007 Jurassic volcaniclastic-basaltic andesite-dolerite sequence in Tasmania: New age constraints for fossil plants from Lune River. *Australian Journal of Earth Sciences* **54**, 965-974. (doi:10.1080/08120090701488297).
105. Regalado L., Schmidt A.R., Müller P., Kobbert M.J., Schneider H., Heinrichs J. 2017 The first fossil of Lindsaeaceae (Polypodiales) from the Cretaceous amber forest of Myanmar. *Cretaceous Research* **72**, 8-12. (doi:https://doi.org/10.1016/j.cretres.2016.12.003).
106. Krassilov V., Bacchia F. 2000 Cenomanian florule of Nammoura, Lebanon. *Cretaceous Research* **21**(6), 785-799. (doi:https://doi.org/10.1006/cres.2000.0229).
107. Schneider H., Schuettpelz E., Pryer K., Cranfill R., Magallon S., Lupia Ii R. 2004 Ferns diversified in the shade of angiosperms. *Nature* **428**, 553-557. (doi:10.1038/nature02361).

108. Upchurch G., Mack G. 1998 Latest Cretaceous leaf megafloras from the Jose Creek Member, McRae Formation of New Mexico. *New Mexico Geological Society Guidebook* **49**, 209-222.
109. Amato J.M., Mack G.H., Jonell T.N., Seager W.R., Upchurch G.R. 2017 Onset of the Laramide orogeny and associated magmatism in southern New Mexico based on U-Pb geochronology. *GSA Bulletin* **129**(9-10), 1209-1226.
110. Regalado L., Schmidt A., Krings M., Bechteler J., Schneider H., Heinrichs J. 2017 Fossil evidence of eupolypod ferns in the mid-Cretaceous of Myanmar. *Plant Systematics and Evolution*. (doi:10.1007/s00606-017-1439-2).
111. Guerra-Sommer M. 1981 Plano-lenhoso vinculado a Sigillariaceae no Irati de S. Paulo. *SIMPÓSIO REGIONAL DE GEOLOGIA* **3**, 176-179.
112. Spiekermann R., Jasper A., Siegloch A.M., Guerra-Sommer M., Uhl D. 2021 Not a lycopsid but a cycad-like plant: Iratinia australis gen. nov. et sp. nov. from the Irati Formation, Kungurian of the Paraná Basin, Brazil. *Review of Palaeobotany and Palynology* **289**, 104415. (doi:https://doi.org/10.1016/j.revpalbo.2021.104415).
113. Rocha-Campos A., Basei M.A.S., Nutman A.P., Santos P., Passarelli C.R., Canile F., Rosa O., Fernandes M., Santa Ana H., Veroslavsky G. 2019 U-Pb zircon dating of ash fall deposits from the paleozoic Paraná basin of Brazil and Uruguay: a reevaluation of the stratigraphic correlations. *The Journal of Geology* **127**(2), 167-182.
114. Rocha H., Mendes M., Pereira Z., Rodrigues C., Fernandes P., Lopes G., Sant'Anna L., Tassinari C., de Sousa M.L. 2020 New palynostratigraphic data of the Irati (Assistência Member) and the Corumbataí formations, Paraná Basin, Brazil, and correlation with other south American basins. *Journal of South American Earth Sciences* **102**, 102631.
115. Florin R. 1948 On the Morphology and Relationships on the Taxaceae. *Botanical Gazette* **110**(1), 31-39. (doi:10.1086/335515).
116. Dong C., Shi G., Herrera F., Wang Y., Herendeen P.S., Crane P.R. 2020 Middle–Late Jurassic fossils from northeastern China reveal morphological stasis in the catkin-yew. *National Science Review* **7**(11), 1765-1767. (doi:10.1093/nsr/nwaa138).
117. Sivhed U. 1984 *Litho-and biostratigraphy of the Upper Triassic-Middle Jurassic in Scania, southern Sweden*, Sveriges geologiska undersökning.
118. Pott C., Mcloughlin S. 2011 The Rhaetian flora of Rögla, northern Scania, Sweden. *Palaeontology* **54**(5), 1025-1051.
119. McElwain J., Beerling D., Woodward F. 1999 Fossil plants and global warming at the Triassic-Jurassic boundary. *Science* **285**(5432), 1386-1390.
120. Harris T. 1931 Rhaetic floras. *Biological reviews* **6**(2), 133-162.
121. McIver E. 1994 An early Chamaecyparis (Cupressaceae) from the Late Cretaceous of Vancouver Island, British Columbia, Canada. *Canadian journal of botany* **72**(12), 1787-1796.
122. Stockey R.A., Kvacek J., Hill R.S., Rothwell G.W., Kvacek Z. 2005 The fossil record of Cupressaceae s. lat. *A monograph of Cupressaceae and Sciadopitys* **54**, 68.
123. Bell W.A. 1957 Flora of the Upper Cretaceous Nanaimo Group of Vancouver Island, British Columbia. *Geological Survey of Canada, Memoir* (293), 1-84.
124. Rothwell G.W., Mapes G., Stockey R.A., Hilton J. 2012 The seed cone Eathiestrobus gen. nov.: fossil evidence for a Jurassic origin of Pinaceae. *Am J Bot* **99**(4), 708-720. (doi:10.3732/ajb.1100595).
125. Riding J.B. 2005 Middle and Upper Jurassic (Callovian to Kimmeridgian) palynology of the onshore Moray Firth Basin, northeast Scotland. *Palynology* **29**(1), 87-142.
126. Cope J.C.W. 1980 *A Correlation of Jurassic rocks in the British Isles: part two: Middle and Upper Jurassic*, Blackwell Scientific Publications.
127. Gradstein F.M., Ogg J.G., Schmitz M., Ogg G. 2012 *The geologic time scale 2012*, elsevier.

128. Ryberg P.E., Rothwell G.W., Stockey R.A., Hilton J., Mapes G., Riding J.B. 2012 Reconsidering Relationships among Stem and Crown Group Pinaceae: Oldest Record of the Genus Pinus from the Early Cretaceous of Yorkshire, United Kingdom. *International Journal of Plant Sciences* **173**(8), 917-932. (doi:10.1086/667228).
129. Duxbury S. 1977 A palynostratigraphy of the Berriasian to Barremian of the Speeton Clay of Speeton, England.
130. Saladin B., Leslie A.B., Wüest R.O., Litsios G., Conti E., Salamin N., Zimmermann N.E. 2017 Fossils matter: improved estimates of divergence times in Pinus reveal older diversification. *BMC evolutionary biology* **17**(1), 95.
131. Robison C.R. 1977 Pinus triphylla and Pinus quinquefolia from the Upper Cretaceous of Massachusetts. *American Journal of Botany* **64**(6), 726-732.
132. Groot J.J., Penny J.S., Groot C.R. 1961 Plant microfossils and age of the Raritan, Tuscaloosa and Magothy formations of the eastern United States. *Palaeontographica Abteilung B*, 121-140.
133. Doyle J.A., Robbins E.I. 1977 Angiosperm pollen zonation of the continental Cretaceous of the Atlantic Coastal Plain and its application to deep wells in the Salisbury Embayment.
134. Axelrod D.I. 1986 Cenozoic History of Some Western American Pines. *Annals of the Missouri Botanical Garden* **73**(3), 565-641. (doi:10.2307/2399194).
135. Evernden J.F., Savage D.E., Curtis G.H., James G.T. 1964 Potassium-argon dates and the Cenozoic mammalian chronology of North America. *American Journal of Science* **262**(2), 145-198. (doi:10.2475/ajs.262.2.145).
136. Barba-Montoya J., dos Reis M., Schneider H., Donoghue P.C., Yang Z. 2018 Constraining uncertainty in the timescale of angiosperm evolution and the veracity of a Cretaceous Terrestrial Revolution. *New Phytologist* **218**(2), 819-834.
137. Coiffard C., Kardjilov N., Manke I., Bernardes-de-Oliveira M.E.C. 2019 Fossil evidence of core monocots in the Early Cretaceous. *Nature Plants* **5**(7), 691-696. (doi:10.1038/s41477-019-0468-y).
138. Ogg J., Hinnov L., Huang C. 2012 Cretaceous. In *The geologic time scale* (pp. 793-853, Elsevier.
139. Lúcio T., Souza Neto J.A., Selby D. 2020 Late Barremian / Early Aptian Re–Os age of the Ipubi Formation black shales: Stratigraphic and paleoenvironmental implications for Araripe Basin, northeastern Brazil. *Journal of South American Earth Sciences* **102**, 102699. (doi:https://doi.org/10.1016/j.jsames.2020.102699).
140. Conran J.G., Carpenter R.J., Jordan G.J. 2009 Early eocene Ripogonum (Liliales: Ripogonaceae) leaf macrofossils from southern Australia. *Australian Systematic Botany* **22**(3), 219-228.
141. Macphail M. 2005 Palynostratigraphic analysis of plant microfossils preserved in Early Eocene mudstones at Lowana Road, Strahan, west coast of Tasmania. *Report© prepared for RSH & RJC) Consultant Palynological Services, Aranda, ACT.*
142. Partridge A. 2006 Late Cretaceous–Cenozoic palynology zonations Gippsland Basin. In 'Australian Mesozoic and Cenozoic palynology zonations'.(Coord. E Monteil) Geoscience Australia Record 2006/23. *Chart* **4**.
143. Prasad V., Strömberg C., Leaché A., Samant B., Patnaik R., Tang L., Mohabey D., Ge S., Sahni A. 2011 Late Cretaceous origin of the rice tribe provides evidence for early diversification in Poaceae. *Nature communications* **2**(1), 1-9.
144. Christin P.-A., Spriggs E., Osborne C.P., Strömberg C.A., Salamin N., Edwards E.J. 2014 Molecular dating, evolutionary rates, and the age of the grasses. *Systematic biology* **63**(2), 153-165.

145. Iles W.J., Smith S.Y., Gandolfo M.A., Graham S.W. 2015 Monocot fossils suitable for molecular dating analyses. *Botanical Journal of the Linnean Society* **178**(3), 346-374.
146. Courtillot V.E., Renne P.R. 2003 On the ages of flood basalt events. *Comptes Rendus Geoscience* **335**(1), 113-140.
147. Schoene B., Eddy M.P., Samperton K.M., Keller C.B., Keller G., Adatte T., Khadri S.F.R. 2019 U-Pb constraints on pulsed eruption of the Deccan Traps across the end-Cretaceous mass extinction. *Science* **363**(6429), 862-866. (doi:doi:10.1126/science.aau2422).
148. MacGinitie H.D. 1953 *Fossil plants of the Florissant beds, Colorado*, Carnegie institution of Washington.
149. Romaschenko K., Peterson P., Soreng R. 2010 Phylogenetics of Stipeae (Poaceae: Pooideae) based on plastid and nuclear DNA sequences/Ed. O. Seberg, G. Petersen, AS Barfod and JI Davis. Diversity, phylogeny and evolution in the Monocotyledons. (Aarhus, Denmark: Aarhus University Press.
150. Manchester S. 2001 Update on the megafossil flora of Florissant, Colorado. *ser* **4**, 137-161.
151. II G.P.W.G. 2012 New grass phylogeny resolves deep evolutionary relationships and discovers C4 origins. *New Phytologist* **193**(2), 304-312.
152. Evanoff E., Gregory-Wodzicki K.M., Johnson K. 2001 Fossil flora and stratigraphy of the Florissant Formation, Colorado.
153. PROTHERO D.R., UPTON E. 2016 MAGNETIC STRATIGRAPHY OF THE EOCENE-OLIGOCENE TRANSITION FLORAS AND FAUNAS FROM THE WARNER MOUNTAINS, NORTHEASTERN CALIFORNIA. *Fossil Record 4: Bulletin 67* **67**, 265.

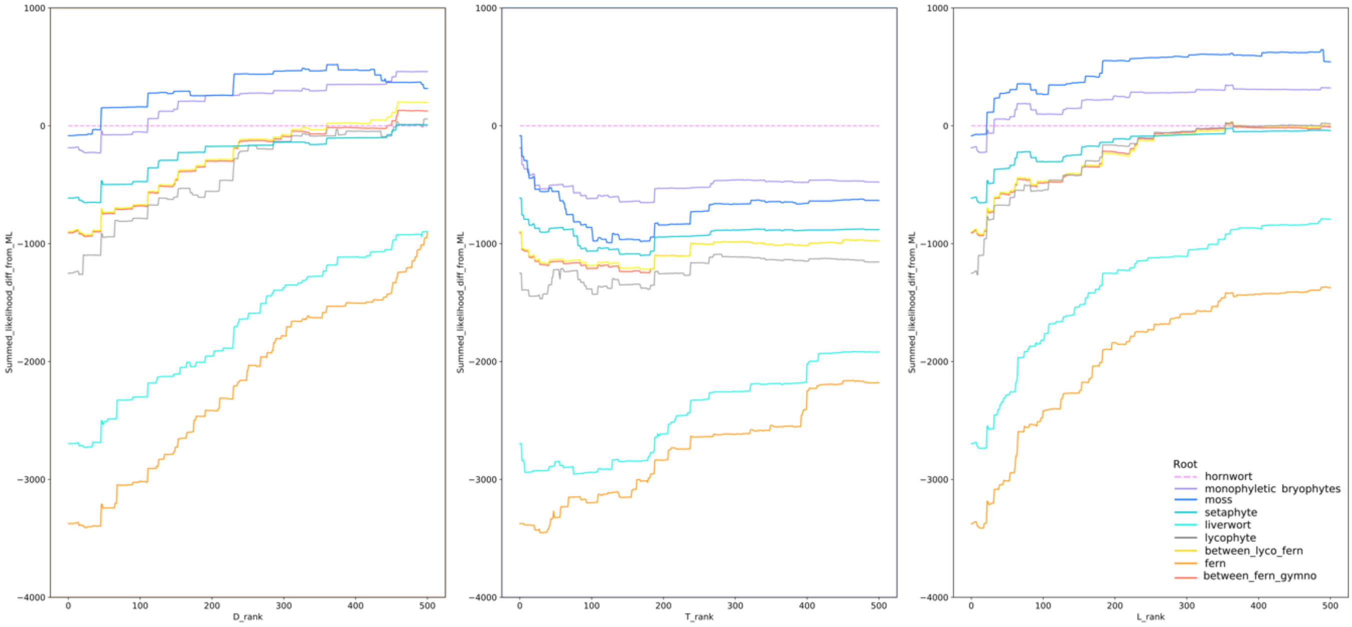

Supplementary Fig. 1 | Evaluating the root signal inferred by ALE. The impact of high duplication, transfer or loss rate families on inferred root order was investigated by iteratively removing top-ranked families and recalculating the summed likelihood on remaining families. While the roots of the credible set remained the top three by summed likelihood, their order varied as high D, T or L-rate families were removed. Removal of high-D and high-L families reduced support for the hornwort root (the ML root on all data), while removal of high-T families increased support for the hornwort root relative to the second- and third-ranked roots. As D, T and L rates were calculated based on the ML root, the preference of high-T families for alternative roots might reflect the difficulty of placing the sparsely-sampled hornworts accurately in individual gene trees.

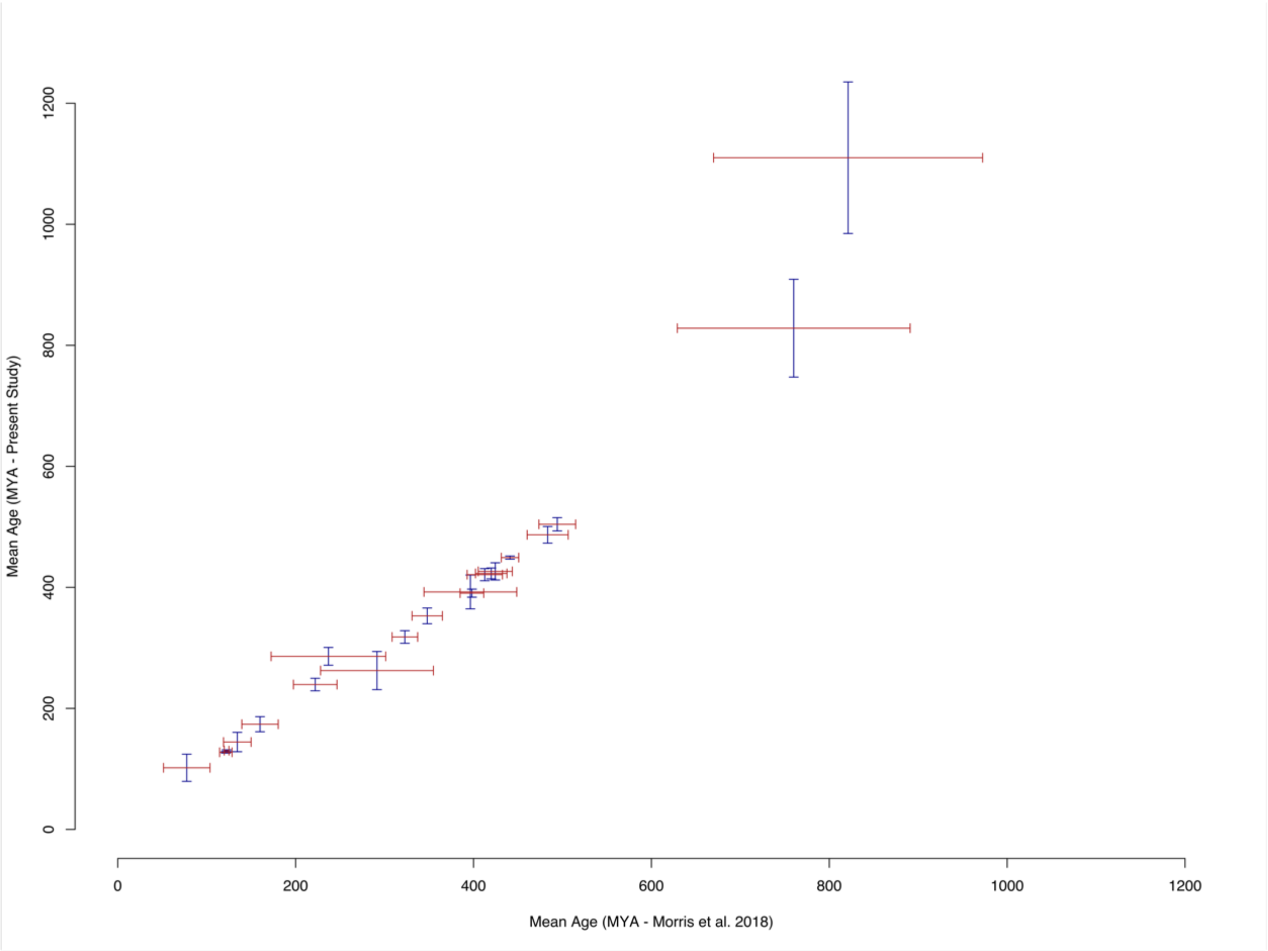

Supplementary Fig. 2 | A comparison of divergence time estimates between the current study and the closest benchmark, that of Morris et al 2018. Points on each axis are centred on the mean age, with the 95% highest posterior density represented by the width of the bars. 95% HPDs were calculated from 2,000 post-burnin samples over 2,000,000 MCMC generations.

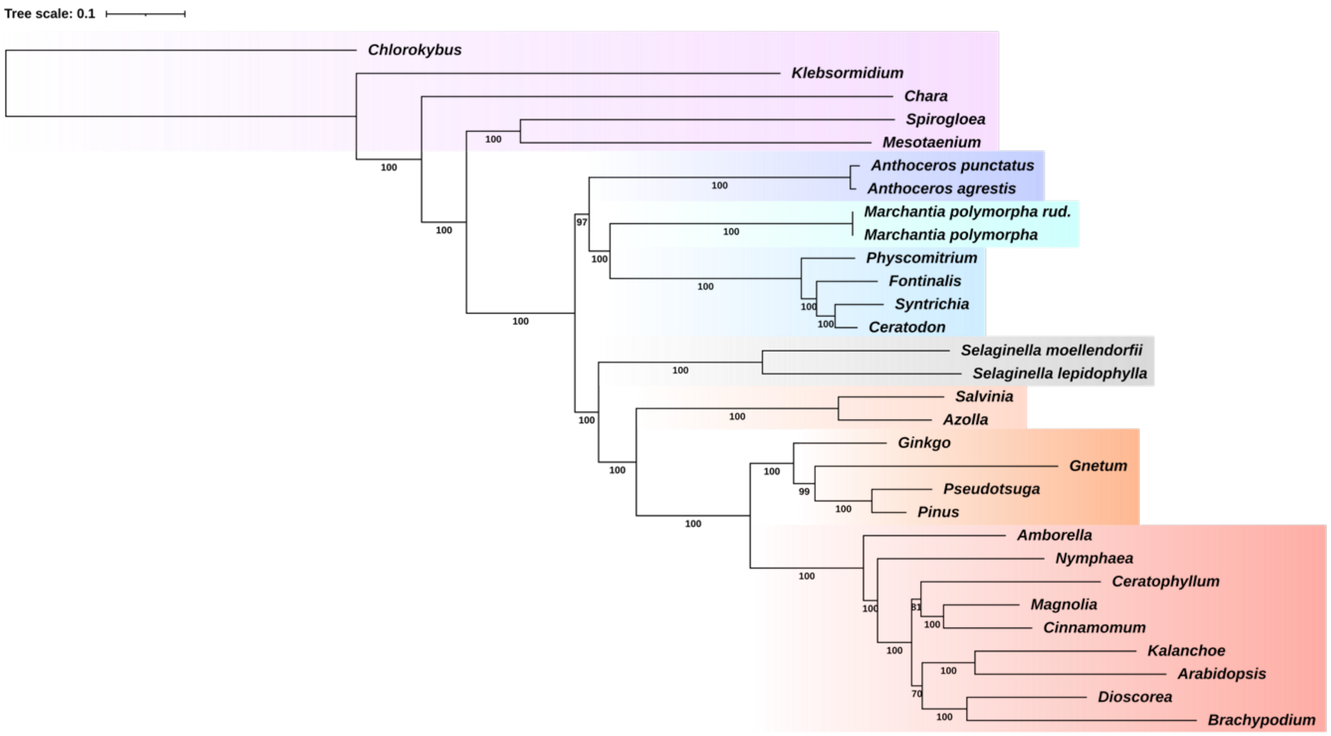

Supplementary Fig. 3 | Phylogenetic tree of embryophytes used for ancestral reconstruction. The maximum likelihood tree was inferred from an alignment of 30 species, 185 single copy orthologs and 71855 sites under the LG+C60+G4+F model in IQ-Tree (Nguyen *et al.,* 2015). Bootstrap support values are placed underneath the corresponding branches. The branch lengths are proportional to the average number of substitutions.

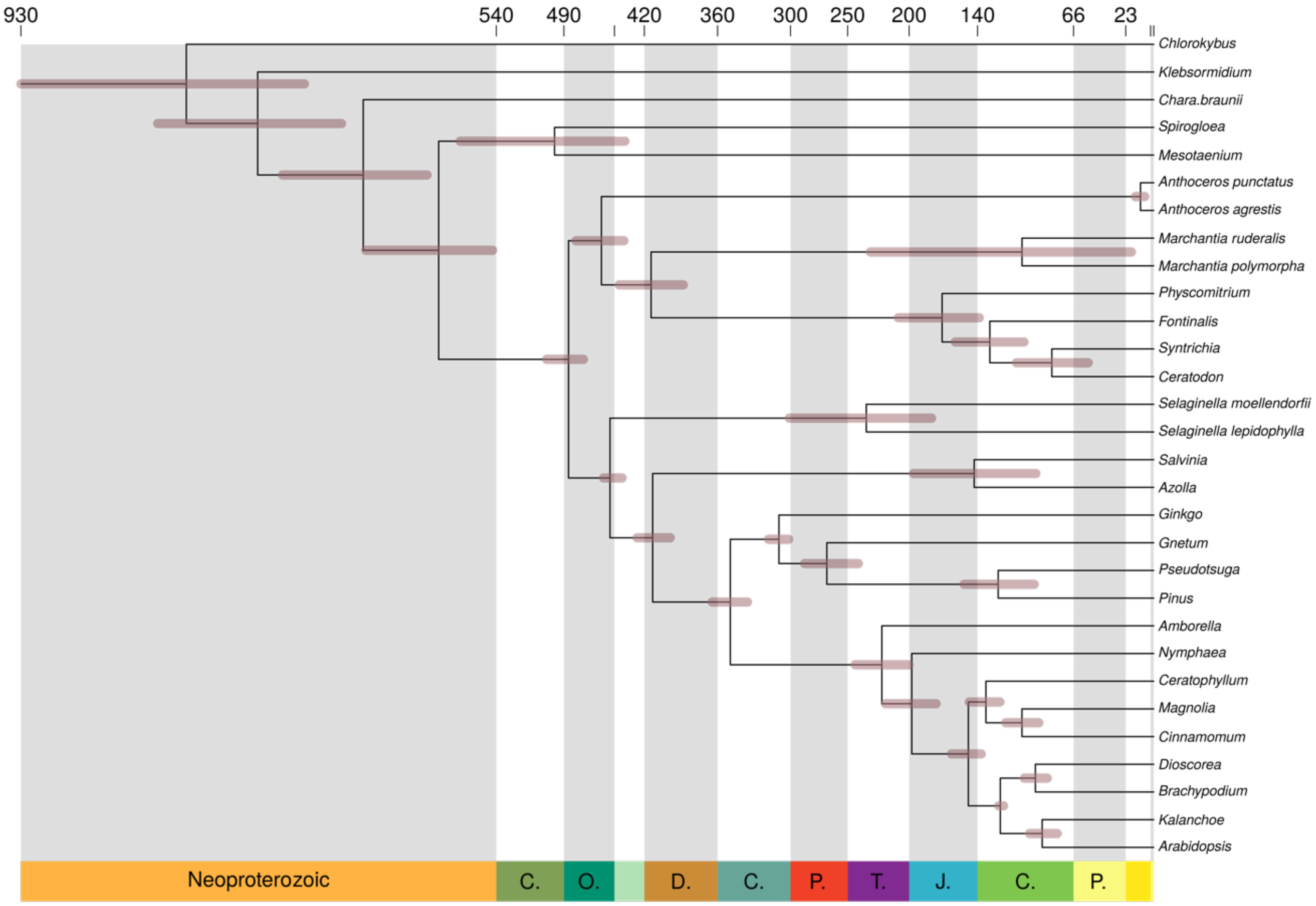

Supplementary Fig 4 | Time scale of embryophyte tree used for ancestral reconstruction. The amino acid sequence alignment was combined with 18 fossil calibrations subsampled from the list of calibrations from the transcriptomic analysis. The normal approximation method was used in MCMCTree, with branch lengths estimated under the LG+G4 model. Calibrations were modelled as a uniform distribution between a minimum and soft maximum. 95% highest posterior density (HPD) width is shown as bars on nodes.

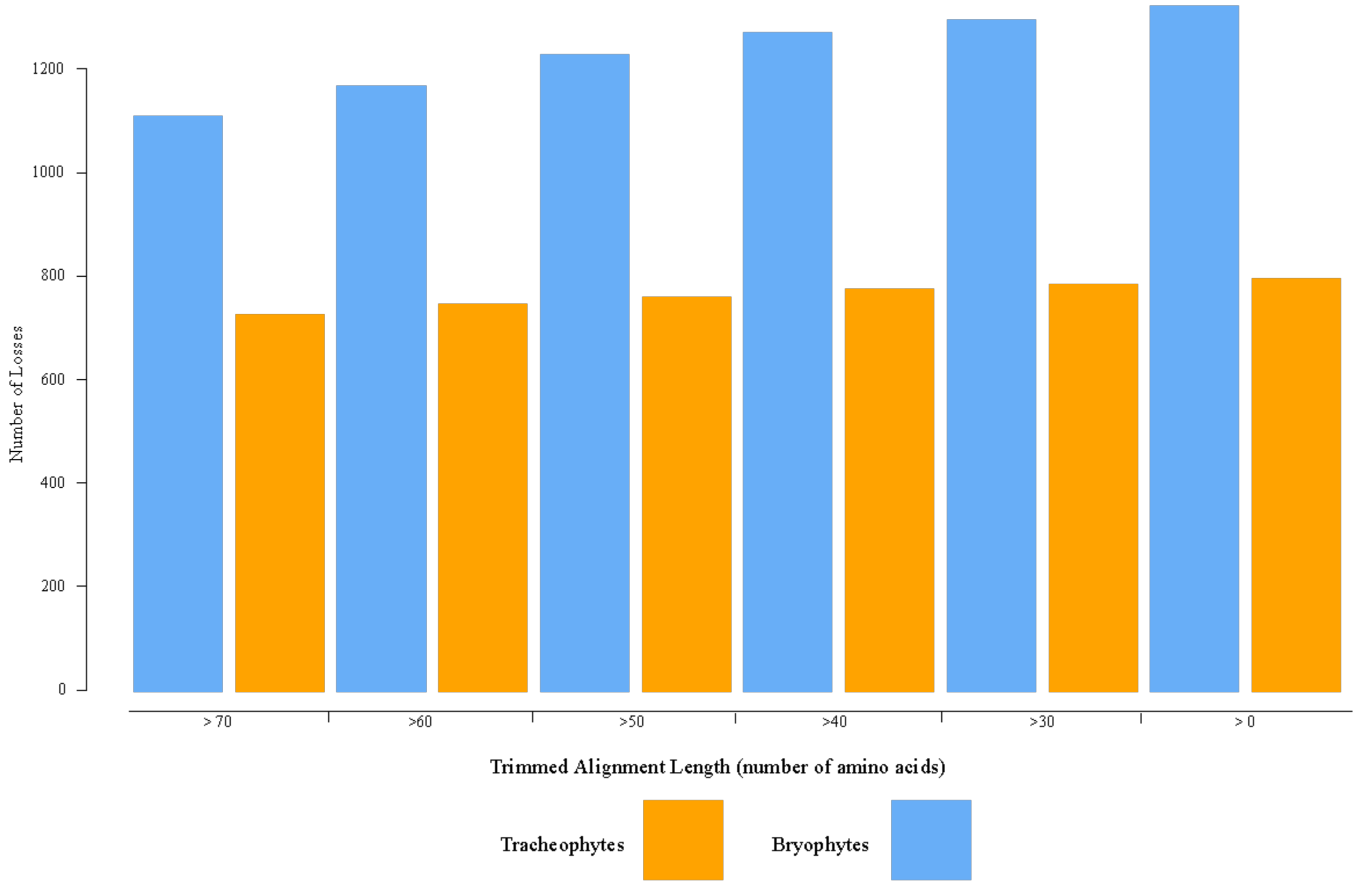

Supplementary Fig. 5 | Filtering gene families by alignment length. The inferred number of losses on the bryophyte (blue) and tracheophyte (orange) stem lineage after filtering the trimmed gene families by their alignment length - that is, discarding gene families with trimmed alignments shorter than a given threshold.

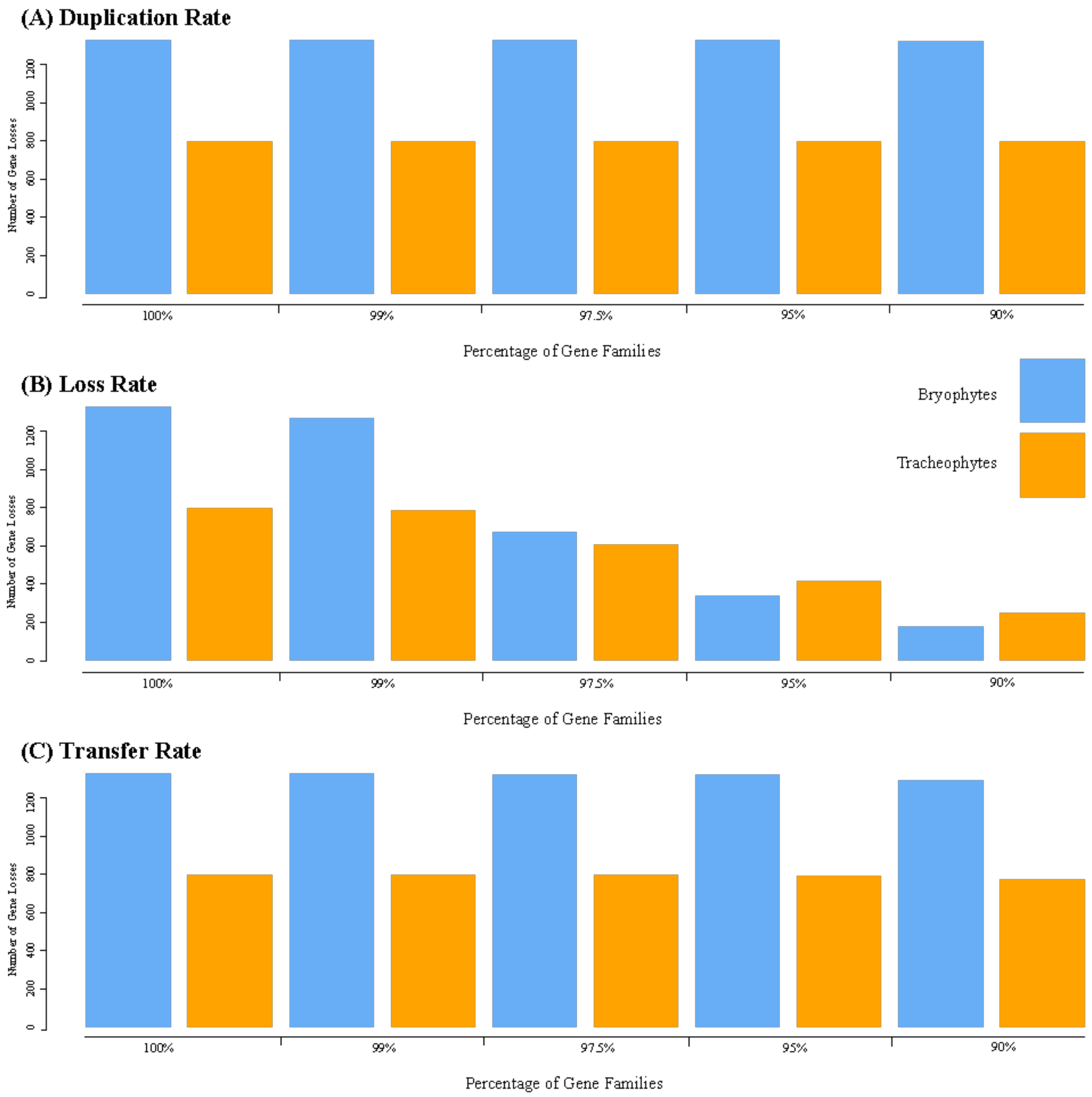

Supplementary Fig. 6 | Filtering gene families by rates of duplication, loss and transfer. The inferred number of losses on the bryophyte (blue) and tracheophyte (orange) stem lineage after filtering the trimmed gene families by their (A) duplication rate (B) loss rate and (C) transfer rate. In each case, families were filtered to remove the top 1, 2.5, 5 and 10% of families. Filtering by duplication or transfer rate had no substantial impact on bryophyte or tracheophyte stem losses. As expected, filtering families by loss rate (B) reduced the number of inferred losses in the remaining families on both the bryophyte and tracheophyte stems. The reduction in losses was greater for the bryophyte stem, suggesting that the greatest number of losses are concentrated in a minority of gene families (see main text for discussion of some particularly notable losses).

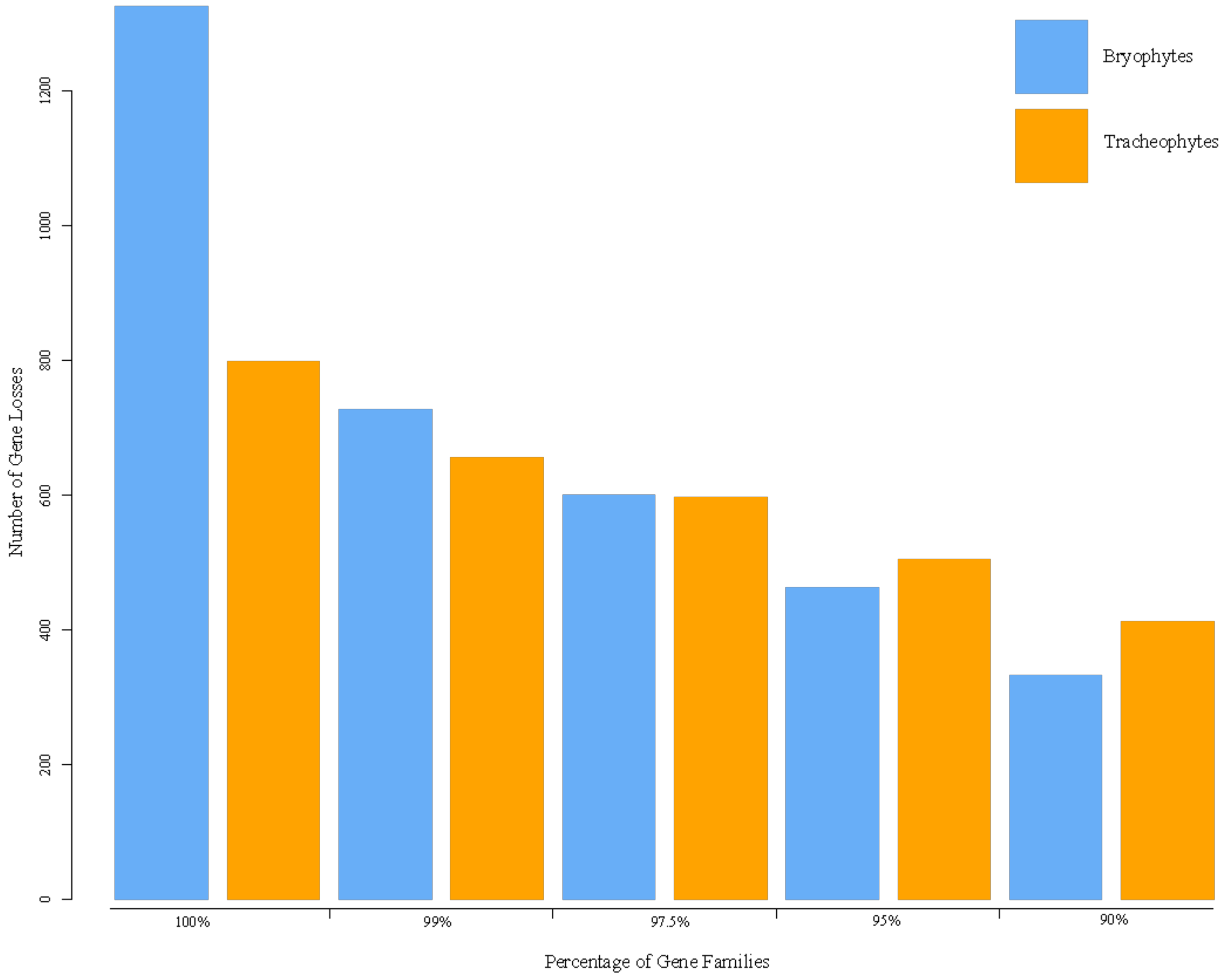

Supplementary Fig. 7 | Filtering gene families by size (number of genes). The inferred number of losses on the bryophyte (blue) and tracheophyte (orange) stem lineage after filtering the trimmed gene families by their size (number of genes). Families were filtered to remove the top 1, 2.5, 5 and 10% of families.

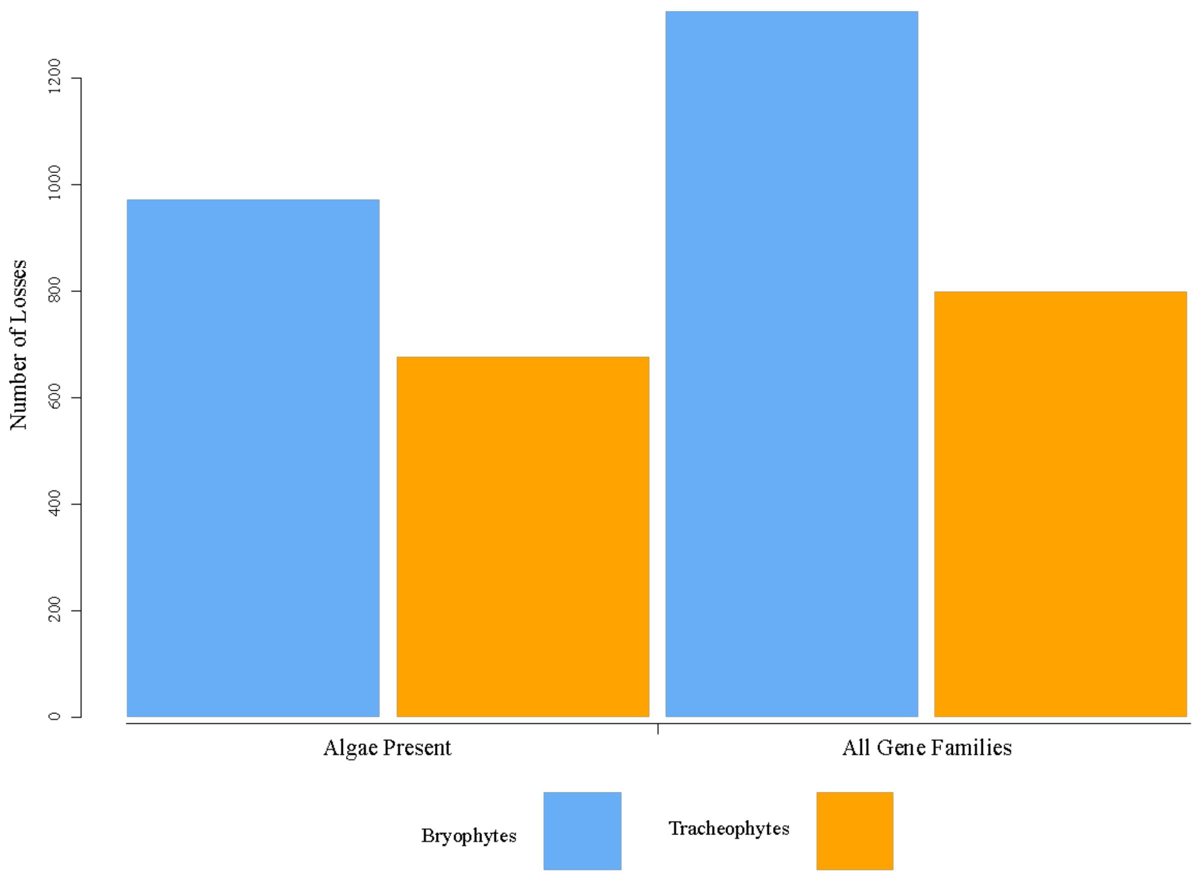

Supplementary Fig. 8 | Filtering gene families by algal copies. The inferred number of losses on the bryophyte (blue) and tracheophyte (orange) stem lineage after filtering the trimmed gene families such that they must include at least 1 copy in 1 of the algal lineages (Algae Present).

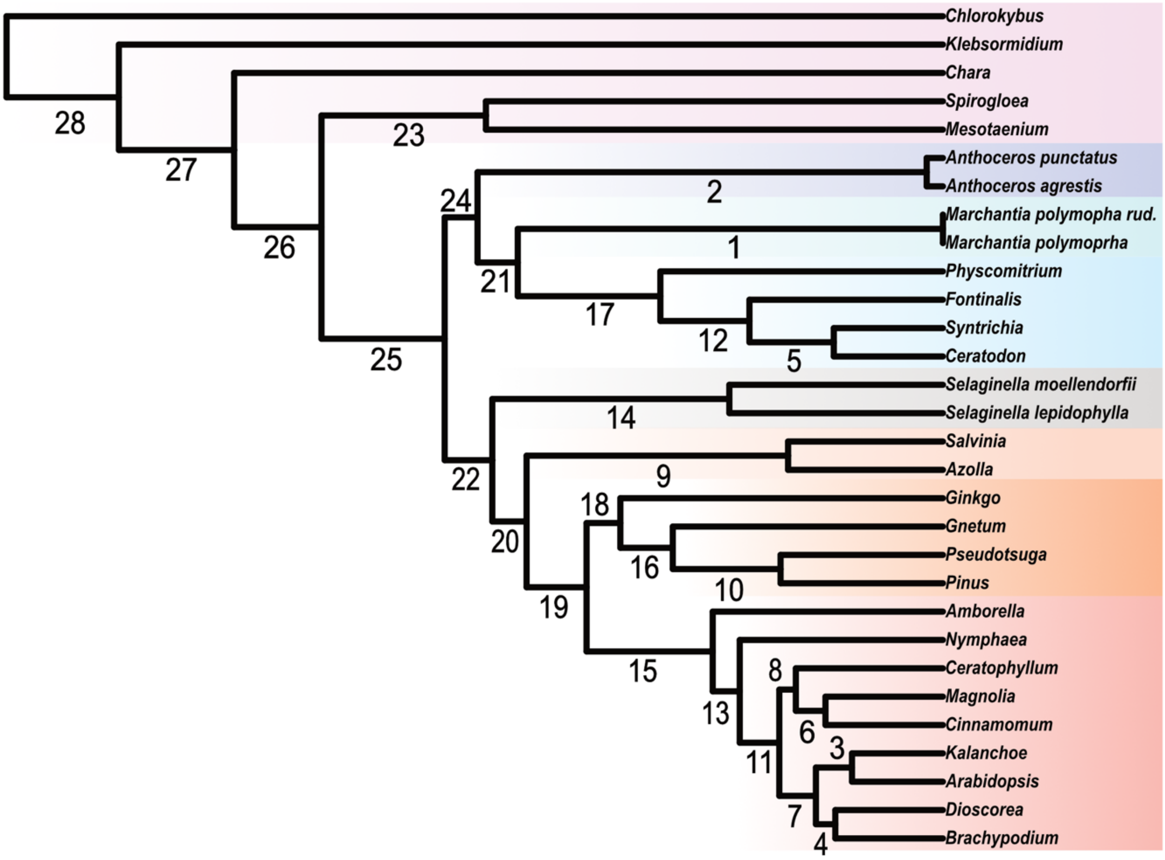

Supplementary Fig. 9 | Gain and loss of orthologous gene family clusters across the embryophyte tree (reference species tree). A maximum likelihood time- calibrated tree, with labels assigned to each branch. See Supplementary Fig. 10 for gains and losses of orthologous gene family clusters mapped to each branch of this reference tree.

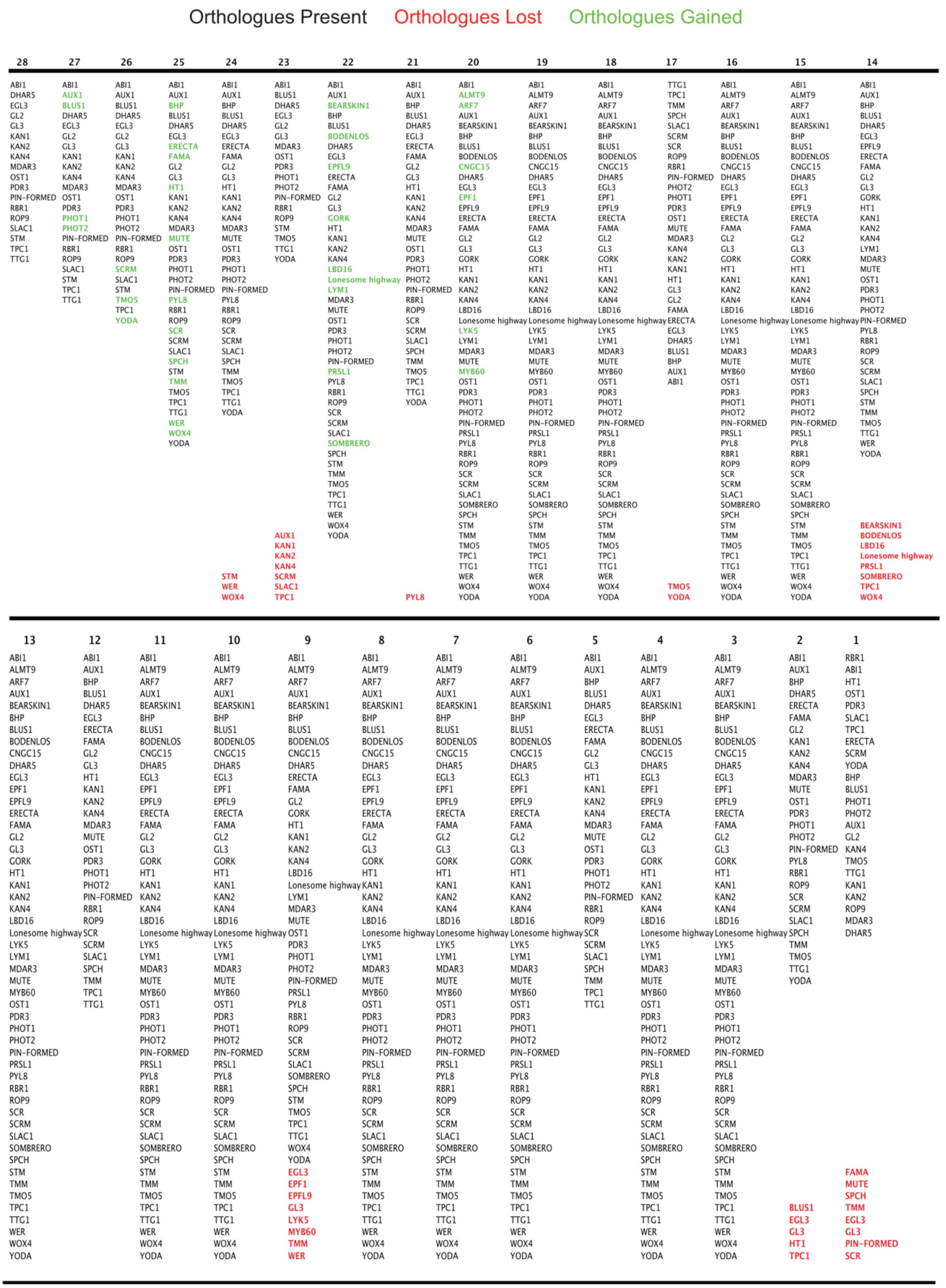

Supplementary Fig. 10 | Gain and loss of orthologous gene family clusters across the embryophyte tree. The labels correspond to a list of the key orthologous gene family clusters present at each branch. Fifty critical genes for Arabidopsis, stomata, vasculature and symbiosis related function/development were used to illustrate the gain and loss of orthologous gene family clusters across the embryophyte tree. Gene families are labelled according to their characterised representatives; for example, the “SLAC1” gene family is the family that contains Arabidopsis SLAC1. Gene family clusters that originated on the branch are coloured in green, whilst ones that are lost are coloured red and all genes present on the branch are in black.

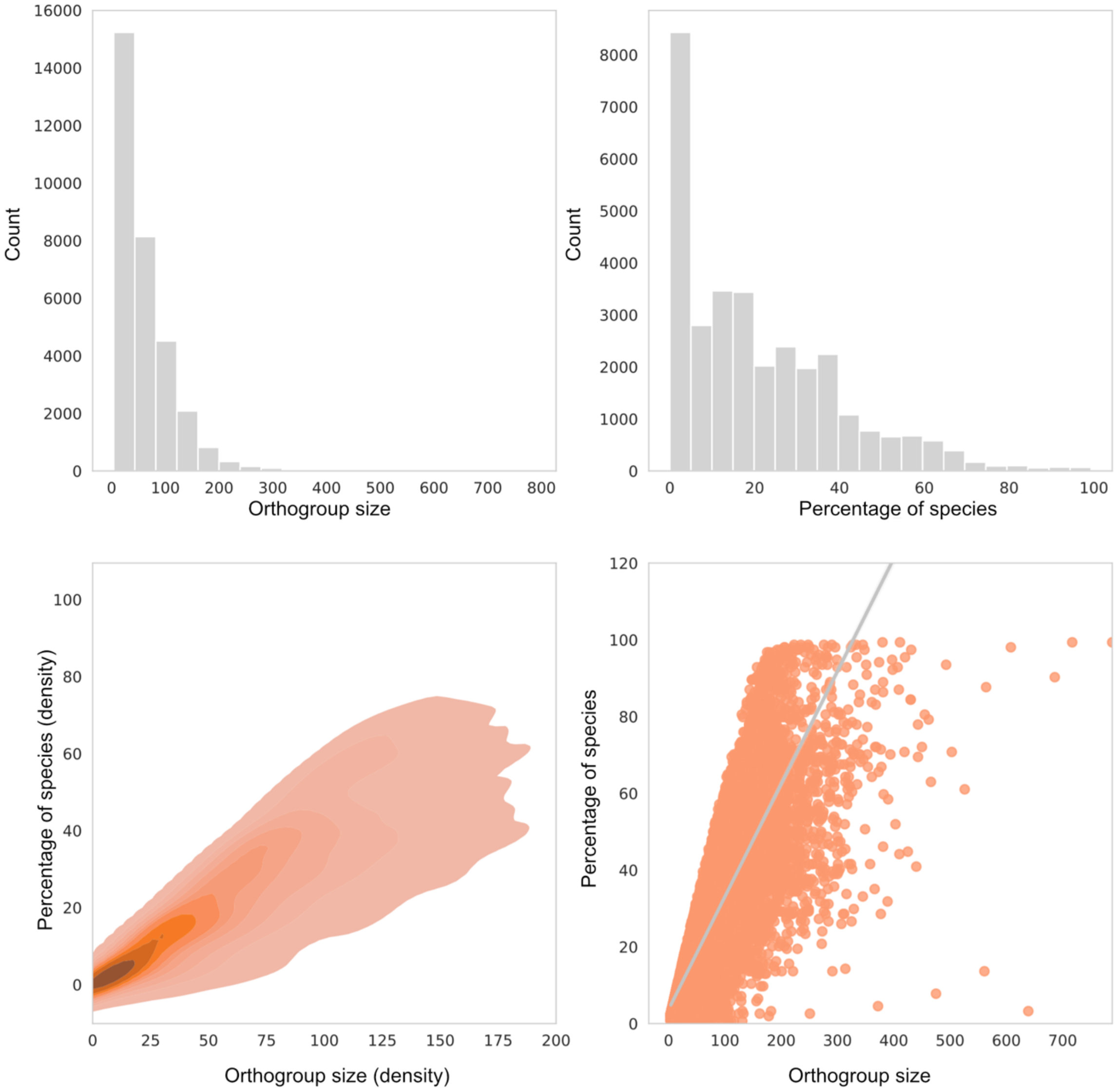

Supplementary Fig. 11 | Gene family clusters for genome and transcriptome dataset – 154 species. Top left, distribution of gene family size. Top right, distribution of gene family species representation. Bottom left, density of gene family species representation and size. Bottom right, regression of gene family species representation and size.

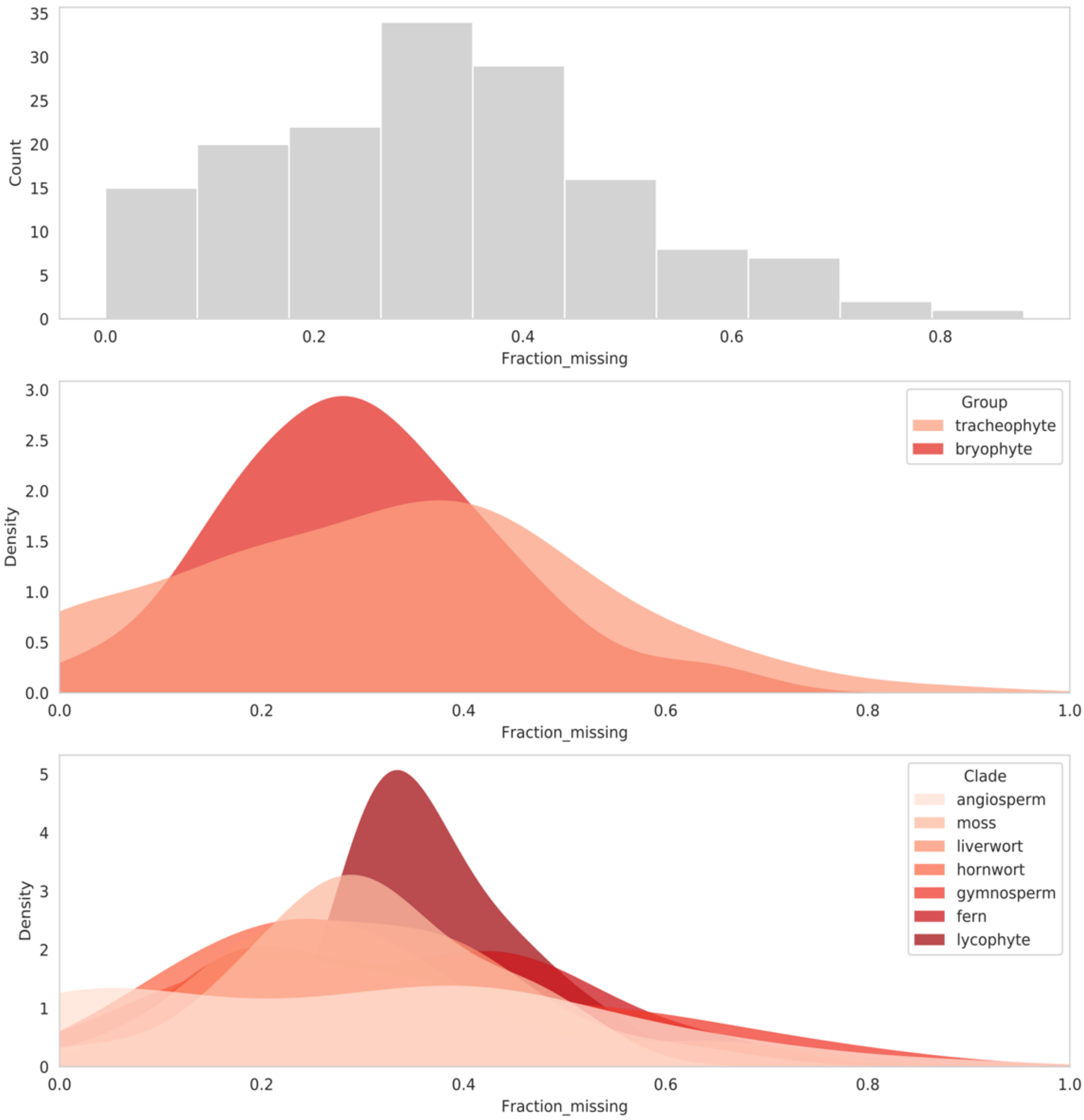

Supplementary Fig. 12 | BUSCO completion of genome and transcriptome dataset – 154 species. Top, histogram of BUSCO completeness. Middle, distribution of BUSCO completeness separated into the two main plant groups. Bryophytes, on average, have more complete BUSCOs than tracheophytes. Bottom, distribution of BUSCO completeness separated by lineage. Angiosperms had the most varied completeness values. As the dataset comprised of both genomes and transcriptomes, the underlying variation per lineage may be ascribed to the more incomplete transcriptomes.

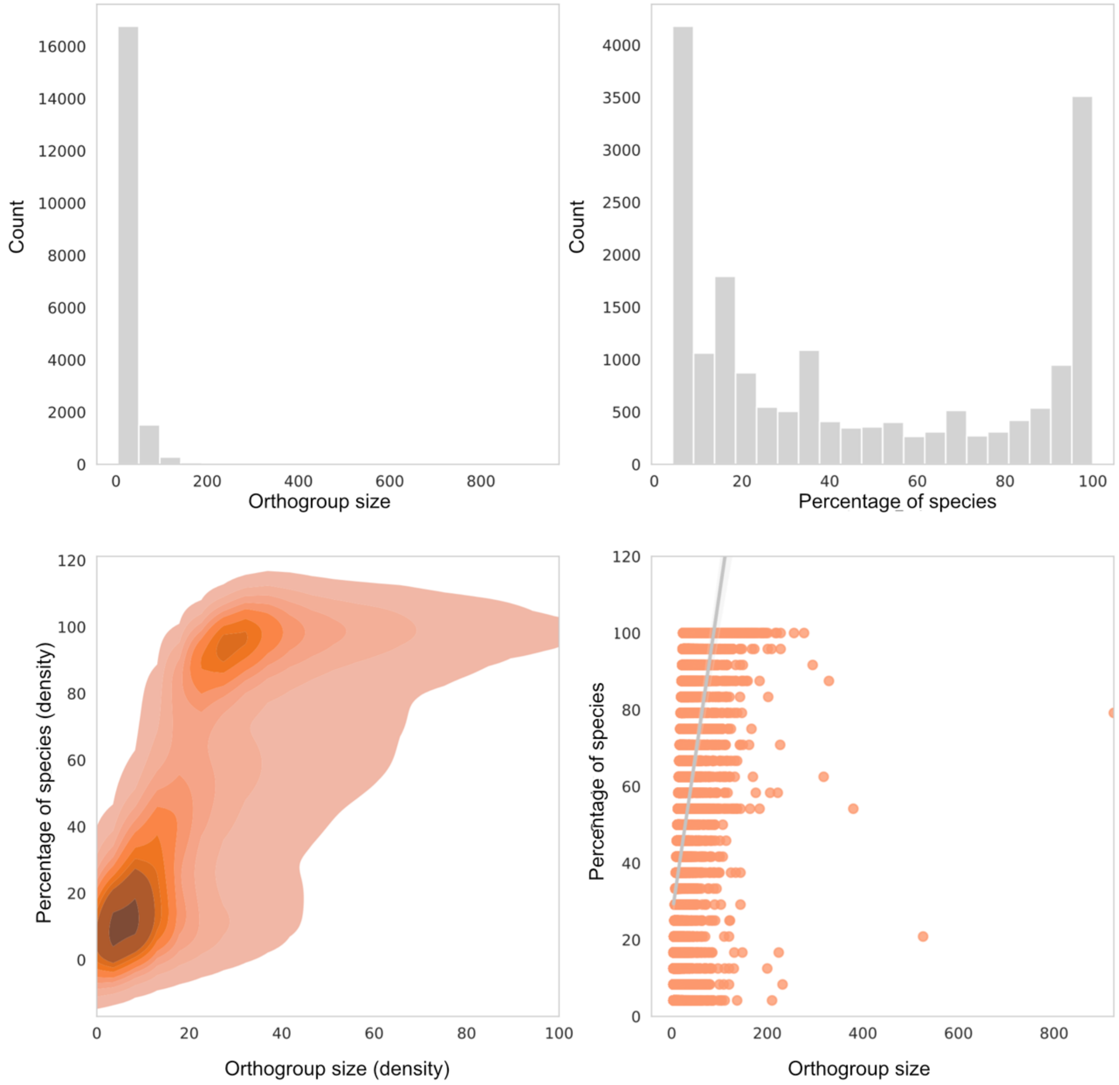

Supplementary Fig. 13 | Gene family clusters for genome dataset - 24 species. Top left, distribution of gene family size. Top right, distribution of gene family species representation. Bottom left, density of gene family species representation and size. Bottom right, regression of gene family species representation and size.

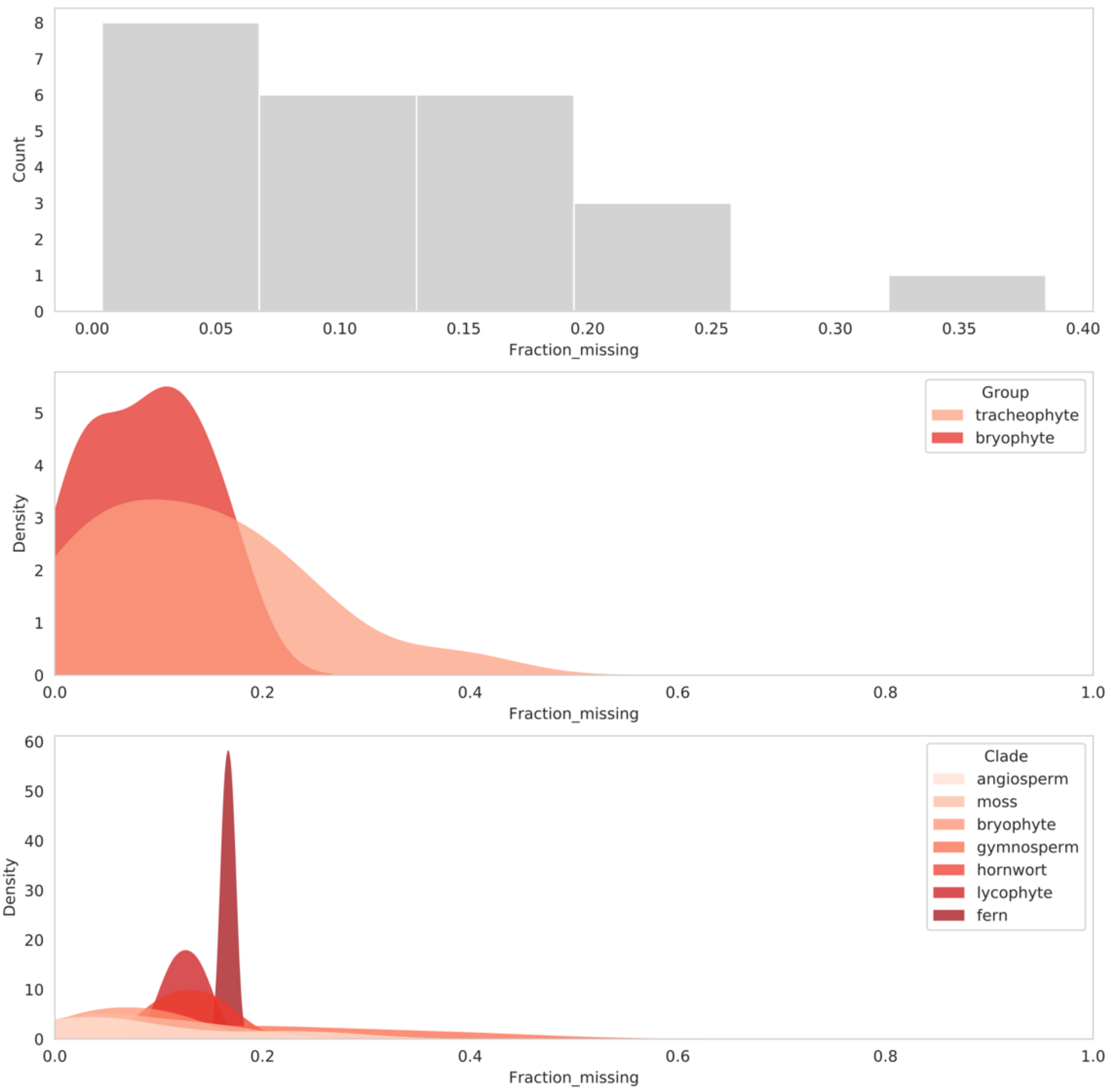

Supplementary Fig. 14 | BUSCO completion of genome only dataset – 24 species. Top, histogram of BUSCO completeness. Middle, distribution of BUSCO completeness separated into the two main plant groups. Bryophytes, on average having more complete BUSCOs than tracheophytes. Bottom, distribution of genome completeness separated by lineage. BUSCO completeness is significantly higher for the genome only dataset.

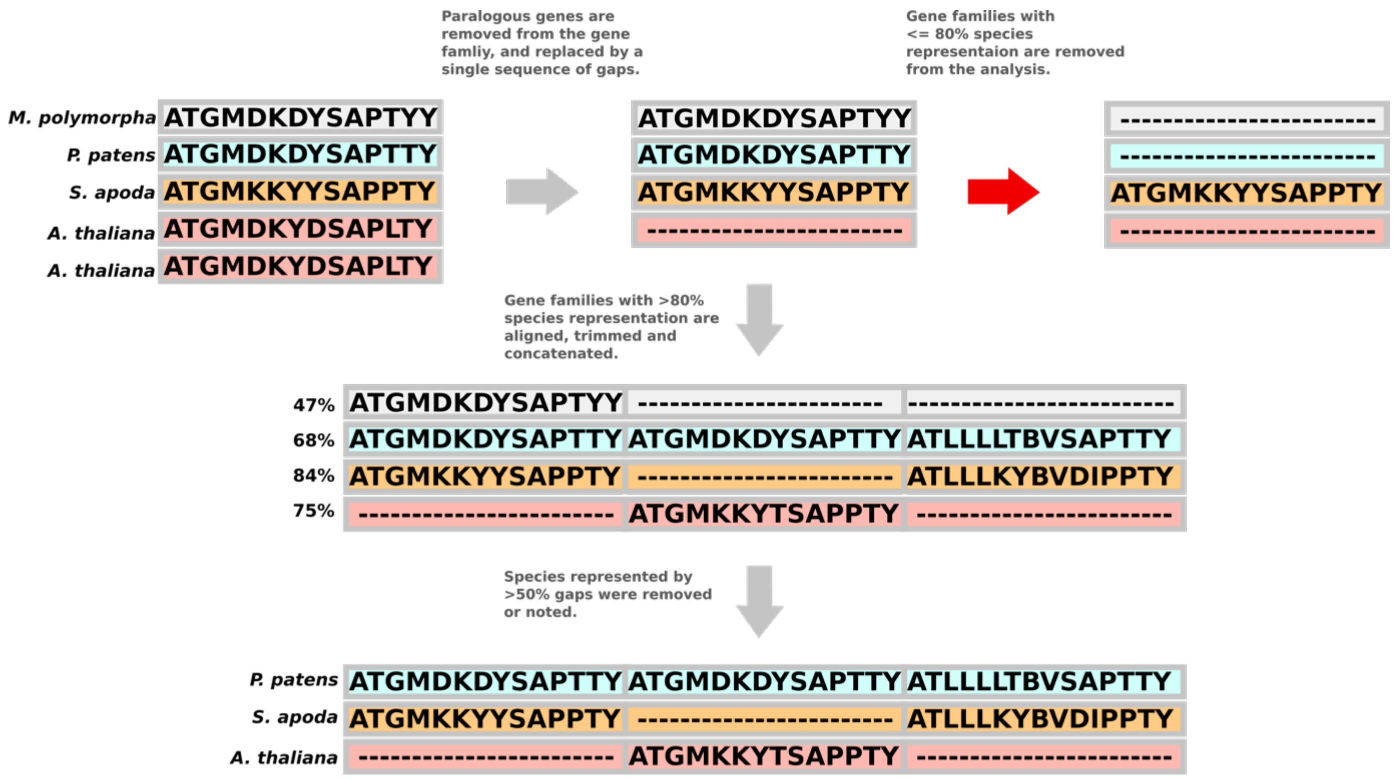

Supplementary Fig. 15 | Graphical representation of orthology inference approach. Species represented by more than one gene copy are removed from the gene family alignment and replaced by a single sequence of gaps. Gene families with more than 80% of the original species present are retained. Gene families are then aligned, trimmed and concatenated together to form a super matrix. Species with less than 50% gaps in the alignment are removed or noted if within 10% error – this step prevents single species that are continually removed from gene families to be in the final alignment.
