# Supplementary material for: Divergent evolutionary trajectories of bryophytes and tracheophytes from a complex common ancestor of land plants
Source: Nat Ecol Evol. 2022 Sep 29;6(11):1634–43. doi: 10.1038/s41559-022-01885-x (PMC9630106; doi:10.1038/s41559-022-01885-x)
Supplement: Supplementary file 2 — Reporting Summary [file 41559_2022_1885_MOESM2_ESM.pdf]

nature portfolio

Corresponding author(s): Tom A. Williams

Last updated by author(s): Aug 2, 2022

Reporting Summary

Nature Portfolio wishes to improve the reproducibility of the work that we publish. This form provides structure for consistency and transparency in reporting. For further information on Nature Portfolio policies, see our Editorial Policies and the Editorial Policy Checklist.

Statistics

For all statistical analyses, confirm that the following items are present in the figure legend, table legend, main text, or Methods section.

n/a	Confirmed	
☐	☒	The exact sample size (*n*) for each experimental group/condition, given as a discrete number and unit of measurement
☐	☒	A statement on whether measurements were taken from distinct samples or whether the same sample was measured repeatedly
☐	☒	The statistical test(s) used AND whether they are one- or two-sided<br>*Only common tests should be described solely by name; describe more complex techniques in the Methods section.*
☒	☐	A description of all covariates tested
☐	☒	A description of any assumptions or corrections, such as tests of normality and adjustment for multiple comparisons
☐	☒	A full description of the statistical parameters including central tendency (e.g. means) or other basic estimates (e.g. regression coefficient) AND variation (e.g. standard deviation) or associated estimates of uncertainty (e.g. confidence intervals)
☐	☒	For null hypothesis testing, the test statistic (e.g. *F*, *t*, *r*) with confidence intervals, effect sizes, degrees of freedom and *P* value noted<br>*Give P values as exact values whenever suitable.*
☐	☒	For Bayesian analysis, information on the choice of priors and Markov chain Monte Carlo settings
☒	☐	For hierarchical and complex designs, identification of the appropriate level for tests and full reporting of outcomes
☒	☐	Estimates of effect sizes (e.g. Cohen's *d*, Pearson's *r*), indicating how they were calculated

Our web collection on statistics for biologists contains articles on many of the points above.

Software and code

Policy information about availability of computer code

Data collection	All of the custom code used for both data collection and analysis is available in a Github repository (https://github.com/ak-andromeda/ALE_methods/). Data collection involved downloading published genomic and transcriptomic data from online repositories; the provenance of all datasets used is provided in Supplementary Table 12.
Data analysis	All of the custom code used for both data collection and analysis is available in a Github repository (https://github.com/ak-andromeda/ALE_methods/). The molecular dating code used to implement the gene transfer calibration is available at https://github.com/dschrempf/mcmc-date. Data were analysed using BUSCO V4, OrthoFinder 2.0, IQ-TREE 1.6.12, PhyloBayes 2.3, ASTRAL 5.7.6, ALEml_undated 0.5, ALEml 0.5, Tracer 1.7.1, MCMCtree 4.8j]J, Trinity 2.11.0, Trimmomatic 0.39, HMMER 3.3.1, eggNOG-mapper 2, MAFFT 7.4.07, BMGE 1.12, and DIAMOND 2.0.13. The rationale for each analysis, the input data and the parameters used are described in the Methods section of the manuscript.

For manuscripts utilizing custom algorithms or software that are central to the research but not yet described in published literature, software must be made available to editors and reviewers. We strongly encourage code deposition in a community repository (e.g. GitHub). See the Nature Portfolio guidelines for submitting code & software for further information.

Data

Policy information about availability of data

All manuscripts must include a data availability statement. This statement should provide the following information, where applicable:

- Accession codes, unique identifiers, or web links for publicly available datasets
- A description of any restrictions on data availability
- For clinical datasets or third party data, please ensure that the statement adheres to our policy

All data are available on FigShare at https://doi.org/10.6084/m9.figshare.c.5682706.

Human research participants

Policy information about studies involving human research participants and Sex and Gender in Research.

Reporting on sex and gender	N/A
Population characteristics	N/A
Recruitment	N/A
Ethics oversight	N/A

Note that full information on the approval of the study protocol must also be provided in the manuscript.

Field-specific reporting

Please select the one below that is the best fit for your research. If you are not sure, read the appropriate sections before making your selection.

☐ Life sciences ☐ Behavioural & social sciences ☒ Ecological, evolutionary & environmental sciences

For a reference copy of the document with all sections, see nature.com/documents/nr-reporting-summary-flat.pdf

Ecological, evolutionary & environmental sciences study design

All studies must disclose on these points even when the disclosure is negative.

Study description	We performed a range of phylogenetic, molecular clock, and comparative genomic analyses to infer a dated phylogenetic tree of land plants, estimate the timescale of land plant evolution, and reconstruct the gene content of ancestral plants.
Research sample	Our study made use of published genome and transcriptome datasets (the data sources are listed in Supplementary Table 12).
Sampling strategy	Much more genome-scale data now exists for plants than we could include in our analyses, for reasons of computational tractability. We therefore sampled representative genomes from across the known diversity of land plants, using metrics of genome quality (BUSCO) to choose the most appropriate representatives for each group.
Data collection	Data were downloaded from public repositories (NCBI and a range of species- and lineage-specific repositories) by co-first author Brogan Harris.
Timing and spatial scale	Genomes were downloaded between March and une 2020 with the exception of Syntrichia (December 2020).
Data exclusions	Our analysis did not use all published data, for the reasons described above. Data were selected according to quality criteria and phylogenetic position (that is, with the aim of sampling across the known diversity of land plants). Beyond these criteria, we did not deliberately exclude data.
Reproducibility	All of the data analysed in the study are provided in the associated FigShare repository (https://doi.org/10.6084/m9.figshare.c.5682706). As a computational study, the individual analyses can be re-run (or built upon) by the community as needed.
Randomization	As a phylogenetic analysis, the data were not randomized. This is standard community practice in phylogenetics, motivated by the evidence that the best available phylogenetic estimates usually are obtained from representative and broadly-sampled datasets.
Blinding	Blinding was not relevant to our phylogenetic, comparative genomic and molecular dating analyses.

Did the study involve field work? ☐ Yes ☒ No

Reporting for specific materials, systems and methods

We require information from authors about some types of materials, experimental systems and methods used in many studies. Here, indicate whether each material, system or method listed is relevant to your study. If you are not sure if a list item applies to your research, read the appropriate section before selecting a response.

Materials & experimental systems

n/a	Involved in the study
☒	☐ Antibodies
☒	☐ Eukaryotic cell lines
☒	☐ Palaeontology and archaeology
☒	☐ Animals and other organisms
☒	☐ Clinical data
☒	☐ Dual use research of concern

Methods

n/a	Involved in the study
☒	☐ ChIP-seq
☒	☐ Flow cytometry
☒	☐ MRI-based neuroimaging
